# ﻿Africa and Arabia encompass a much greater species diversity in the *Achyranthesaspera* aggregate (Amaranthaceae, achyranthoid clade): Evidence from morphological and chorological data

**DOI:** 10.3897/phytokeys.250.136139

**Published:** 2024-12-20

**Authors:** Alexander P. Sukhorukov, Maria Kushunina, Maya V. Nilova, Cláudia Baider, Alexander N. Sennikov

**Affiliations:** 1 Department of Higher Plants, Biological Faculty, Lomonosov Moscow State University, 119234, Moscow, Russia; 2 Laboratory Herbarium (TK), Tomsk State University, Lenin Ave. 36, 634050, Tomsk, Russia; 3 Department of Plant Physiology, Biological Faculty, Lomonosov Moscow State University, 119234, Moscow, Russia; 4 The Mauritius Herbarium, RE Vaughan Building, Agricultural Services, Ministry of Agro-Industry and Food Security, Réduit, 80835, Mauritius; 5 Botanical Museum, Finnish Museum of Natural History, P.O. Box 7, 00014 University of Helsinki, Helsinki, Finland

**Keywords:** *Achyranthes*, Africa, Arabian Peninsula, Old World, taxonomy

## Abstract

*Achyranthes* in its traditional sense (excluding *Achyropsis* that phylogenetically falls into *Achyranthes*) has been considered to contain a restricted (three to four) number of species in Africa and one or two species in the Arabian Peninsula. The morphology of the type species of the genus, *A.aspera*, has been treated as highly polymorphic, with several varieties recognised by various authors. Not surprisingly, a recent extended phylogeny revealed a non-monophyly of *A.aspera*. We present a deeper insight into morphological characters of the *A.aspera* aggregate together with taxonomic, nomenclatural, ecological, and chorological data based on field investigations and herbarium studies. Instead of one polymorphic species, we accept *A.aspera* s.str., *A.abyssinica*, *A.acuminata*, *A.annua*, *A.mauritiana*, *A.porphyrostachya*, *A.sicula*, and *A.seychellensis***sp. nov.**, all being native to different parts of Africa. In most herbaria, the vast majority of African specimens labelled as *A.aspera* belong to other species, which are being reinstated here. In addition, two well-recognized species, *A.fasciculata* and *A.talbotii* from Tropical East and West Africa, respectively, are also discussed. Moreover, we found that the type of A.asperavar.pubescens as listed in the African and Arabian floras and checklists belongs in fact to an American species *A.fruticosa*, which is absent in the Old World. In place of the misapplied A.asperavar.pubescens, we accept *A.porphyrostachya*, a species described from Myanmar, as a correct name for the populations growing in Africa and Arabia. According to our results, at least 10 native species of *Achyranthes* occur in Africa (or 16 species if *Achyropsis* is merged with *Achyranthes*), which is a major diversity center of the genus. Four species are recorded from the Arabian Peninsula (*A.abyssinica*, *A.annua*, *A.aspera* s.str., *A.porphyrostachya*), and two of them (*A.abyssinica* and *A.annua*) reach their easternmost range limit in this region. As a result, the distribution as well as ecological conditions of each species is now clarified or circumscribed for the first time.

## ﻿Introduction

*Achyranthes* L. is a genus widely distributed in the tropical and warm temperate regions of the world. Since the morphology-based generic circumscription of *Achyranthes* had been settled, the total number of species varied from three to five (Cavaco 1953; Stoffers 1980), six to eight (Cavaco 1962; Townsend 1985, 1993a, 1993b) or even 8 to 12–15 (Robertson 1981; Iamonico 2014). Recently, a phylogenetic analysis of the entire achyranthoid clade (Di Vincenzo et al. 2018) showed that the genus is paraphyletic to *Achyropsis* Benth. & Hook.f. (with six species in Africa: Townsend 1980a) and *Nototrichium* Hillebr. (with three species in the Hawaiian Islands: Lorence 1996).

Concomitantly, *Achyranthesaspera* L., the type species of *Achyranthes* s.str., also appeared non-monophyletic (Di Vincenzo et al. 2018), apparently because of its historically broad taxonomic concept. The heterogeneous circumscription of this species was laid down already in its original description by Linnaeus (1753), who established two varieties in *A.aspera*: α [var.] *sicula* L. and β [var.] *indica* L. The latter variety was determined as a synonym of *A.aspera* s.str. (Cavaco 1962; Townsend 1985), while the former was sometimes elevated to species rank as *A.argentea* Lam. (Schweinfurth 1867) or *A.sicula* (L.) All. (e.g., Tutin 1964; Podlech 1966; Lebrun 1973; Hassan et al. 2023).

To complicate matters, some species described from different continents were synonymized with *A.aspera*, with others being forgotten and unresolved, e.g. *A.acuminata* E.Mey. ex Sonder, *A.annua* Dinter, *A.frumentacea* Burm.f., *A.fruticosa* Lam., *A.pedicellata* Lopr., *A.totonaca* Sessé & Moc., *A.virgata* Poir., and *A.viridis* Lopr. Infraspecific taxa of *A.aspera* were usually distinguished on the basis of life form, leaf shape and color, and perianth length (Moquin-Tandon 1849; Boerlage 1891; Suessenguth 1952; Cavaco 1962; Townsend 1985; Townsend and Darbyshire 2004; Brundu and Camarda 2013; Hassan et al. 2023).

As expected in such a broad circumscription, *A.aspera* exhibits large morphological variability, occurs in a vast geographical range across the tropics and subtropics, and shows a wide ecological amplitude by growing in deserts, evergreen forests, riverine habitats, and agricultural and urban environments. Worryingly, under this taxonomic uncertainty, plants collectively called *A.aspera* have been widely used in traditional healing with numerous applications (Maiden 1889; Hauman 1951; Burkill 1985; Naidu et al. 2006; Ndhlala et al. 2015; Yadav et al. 2016; He et al. 2017), and are being studied for conventional medicine (Zanzabil et al. 2023; Lin et al. 2024).

Currently, based on the literature, the number of *Achyranthes* species in Africa is estimated at 4 to 5. Traditionally, two species were reported as widely distributed: *A.aspera* (Hooker 1849; Lebrun and Stork 2003; Klopper et al. 2006; APD 2024) including var.pubescens auct. (Townsend 1985, 2000; Germishuizen and Meyer 2003; Gibreel and Darbyshire 2015; Odorico et al. 2022), and *A.sicula* (Lebrun 1973; Townsend 2000). The latter was named *A.argentea* in early taxonomic works (Hooker 1849) or accepted occasionally at varietal level as A.asperavar.sicula L. (Maire 1962; Townsend 1985, 2000; Germishuizen and Meyer 2003; Gibreel and Darbyshire 2015; Odorico et al. 2022; Gosline et al. 2023) or A.asperavar.argentea Eggers (Baker and Clarke 1909; Hauman 1951), or even treated under both varietal names simultaneously (Sunding 1973). Two other accepted species have a limited distribution: *A.talbotii* Hutch. & Dalziel in Tropical West Africa (Hutchinson and Dalziel 1927; Keay 1954; Townsend and Darbyshire 2004), and *A.fasciculata* (Suess.) C.C.Towns. in Tropical East Africa (Townsend 1980a, 1985). Some authors also reported the presence of the Asian *A.bidentata* Blume in tropical Africa (Baker and Clarke 1909; Hutchinson and Dalziel 1927; Keay 1954; Lebrun and Stork 1991; Phiri 2005; Klopper et al. 2006).

Even within a single floristic area, as in the ‘Flora Zambesiaca’ (Malawi, Mozambique, Zambia, Zimbabwe, and Caprivi Strip [northeastern corner of Namibia]), taxonomic concepts in *Achyranthes* are fairly diverse (Podlech 1966; Townsend 1988; Mapaura et al. 2004; Phiri 2005; Setshogo 2005; Odorico et al. 2022); see also Table 1 for comparison. In the Arabian Peninsula, Miller (1996) accepted a single species, *A.aspera* with three varieties: var.aspera, var.pubescens, and var.sicula. Lately, Hassan et al. (2023) accepted *A.aspera* (incl. var.aspera and var.pubescens) and treated var.sicula at the species rank, as *A.sicula*.

**Table 1.** Comparisons of the taxonomic composition of the *Achyranthesaspera* aggregate in various regions and countries.

Country or floristic region	Previously accepted taxonomy (with references)	New taxonomic circumscription (present paper)
**Africa**		
Angola	*Achyranthesaspera* s.str., A.asperavar.porphyrostachya, A.asperavar.sicula, *A.bidentata* (Figueiredo and Smith 2008)	*Achyranthesabyssinica*, *A.acuminata*, *A.annua*, *A.porphyrostachya*
Burkina Faso	*Achyranthesaspera* s.str., A.asperavar.sicula (Lebrun et al. 1991); *A.aspera* s.str. (Thiombiano et al. 2012)	*Achyranthesannua*
Cape Verde	*Achyranthesaspera* (var.argentea and var.sicula) (Sunding 1973); *Achyranthesaspera* (Arechavaleta et al. 2005)	*Achyranthesannua*, *A.aspera* s.str., *A.porphyrostachya*, *A.sicula*
Central African Republic	*Achyranthesaspera*, A.asperaf.argentea (Boulvert 1977)	*Achyranthesacuminata*, *A.annua*
Chad	*Achyranthesaspera* s.l. (Lebrun et al. 1972); *Achyranthesaspera* s.str., A.asperavar.sicula (Brundu and Camarda 2013; César and Chatelain 2019)	*Achyranthesannua*, *A.aspera* s.str., *A.porphyrostachya*
Comoros and Madagascar	Achyranthesasperavar.indica, A.asperavar.argentea, *A.mauritiana* (Cavaco 1953)	*Achyranthesacuminata* (Madagascar), *A.annua* (Madagascar), *A.aspera* s.str., *Achyranthes* sp. (“velutina”) (Comoros)
Djibouti, Eritrea, Ethiopia	A.asperavar.pubescens, A.asperavar.sicula (Audru et al. 1994; Townsend 2000)	*Achyranthesabyssinica*, *A.acuminata* (Ethiopia), *A.annua*, *A.aspera* s.str. (Ethiopia), *A.porphyrostachya*
Flora Zambesiaca area (Botswana, Malawi, Mozambique, Zambia, Zimbabwe, and northeastern corner of Namibia)	*Achyranthesaspera* s.str., A.asperavar.pubescens, A.asperavar.sicula (Townsend 1988); *Achyranthesaspera* s.str., A.asperavar.indicaf.excelsa, A.asperavar.porphyrostachya (Da Silva et al. 2004); *A.aspera* s.str., A.asperavar.pubescens, A.asperavar.sicula (Mapaura et al. 2004); *Achyranthesaspera* s.str., A.asperavar.pubescens, A.asperavar.sicula (Setshogo 2005); *Achyranthesaspera*, *A.bidentata* (Phiri 2005); Achyranthesasperavar.porphyrostachya, A.asperavar.pubescens, A.asperavar.sicula (Odorico et al. 2022)	*Achyranthesabyssinica* (Malawi), *A.acuminata*, *A.annua*, *A.aspera* s.str. (Zimbabwe), *A.porphyrostachya*
Gabon	*Achyranthesaspera* (Sosef et al. 2006)	*Achyranthesacuminata*
Guinea	*Achyranthesaspera* s.str., A.asperavar.sicula (Gosline et al. 2023)	*Achyranthesacuminata*, *A.annua*
Guinea-Bisau	*Achyranthesaspera* (Catarino et al. 2006)	*Achyranthesannua*
Mascarenes (Mauritius, Réunion, Rodrigues)	*Achyranthesaspera* (Townsend 1994)	*Achyranthesannua* (Réunion), *A.aspera* s.str., *A.mauritiana*
Mali	*Achyranthesaspera* s.str., A.asperavar.sicula (Boudet and Lebrun 1986)	*Achyranthesannua*, *A.porphyrostachya*
Mauritania	*Achyranthesaspera* s.str., A.asperavar.sicula (Barry and Celles 1991)	*Achyranthesannua*
Mayotte	*Achyranthesaspera* (Barthelat 2019)	*Achyranthesacuminata*, *A.aspera* s.str.
Namibia	*Achyranthesaspera* s.str., *A.sicula* (Podlech 1966); *Achyranthesaspera* s.str., A.asperavar.sicula (Klaassen and Kwembeya 2013)	*Achyranthesacuminata*, *A.annua*, *A.aspera* s.str., *A.porphyrostachya*
Niger	*Achyranthessicula* (Peyre de Fabregues and Lebrun 1976)	*Achyranthesannua*, possible records of *A.porphyrostachya*
North Africa (Algeria, Egypt, Libya, Morocco, Tunisia)	Achyranthesasperavar.sicula (Maire 1962); *A.aspera* s.str., A.asperavar.pubescens (Boulos 1995)	*Achyranthesannua* (rare: Egypt), *A.sicula* (Mediterranean part), *A.aspera* s.str. (an old record for Egypt), *A.porphyrostachya* (Egypt)
São Tomé and Príncipe	*Achyranthesaspera* s.str. (Figueiredo et al. 2011)	*Achyranthesacuminata*, *A.aspera* s.str.
Senegal	*Achyranthesargentea*, *A.aspera* (Berhaut 1971); *A.aspera*, *A.sicula*, and probably *A.porphyrostachya* (Lebrun 1973)	*Achyranthesannua*, *A.aspera* s.str., *A.porphyrostachya*
Seychelles	*Achyranthesaspera* s.str., A.asperavar.fruticosa, A.asperavar.velutina (Friedmann 1994)	*Achyranthesaspera*, *A.seychellensis*, *A.* sp. (“*velutina*”).
Somalia	*Achyranthesaspera* s.str., A.asperavar.pubescens, A.asperavar.sicula (Townsend 1993b)	*Achyranthesabyssinica*, *A.annua*, *A.aspera* s.str., *A.porphyrostachya*
South Sudan and Sudan	*Achyranthesaspera* s.str., A.asperavar.pubescens, A.asperavar.sicula (Gibreel and Darbyshire 2015)	*Achyranthesabyssinica*, *A.annua*, *A.aspera* s.str. (South Sudan), *A.porphyrostachya*
South Africa	*Achyranthesaspera* s.str.; A.asperavar.pubescens, A.asperavar.sicula (Germishuizen and Meyer 2003)	*Achyranthesacuminata*, *A.annua*, *A.aspera* s.str., *A.porphyrostachya*
Tropical East africa (Kenya, Tanzania, Uganda)	*Achyranthesaspera* s.str., A.asperavar.pubescens, A.asperavar.sicula (Townsend 1985)	*Achyranthesabyssinica*, *A.acuminata*, *A.annua*, *A.aspera* s.str., *A.porphyrostachya*, *A.reptans*
Tropical West-Central Africa (Burundi, DR Congo, Rwanda)	*Achyranthesaspera* s.str., A.asperavar.argentea (Hauman 1951); *Achyranthesaspera* s.str., A.asperavar.sicula (Lejoly et al. 2010)	*Achyranthesabyssinica*, *A.acuminata*, *A.annua*, *A.aspera* s.str., *A.porphyrostachya*
West Tropical Africa (Benin, Cameroon, Ghana, Ivory Coast, Liberia, Nigeria, Sierra Leone, Togo)	*Achyranthesaspera* s.str., *A.bidentata* (Keay 1954)	*Achyranthesabyssinica* (Cameroon, Nigeria), *A.acuminata*, *A.annua* (no data from Ivory Coast), *A.aspera* s.str. (Benin, Cameroon, Ghana), *A.porphyrostachya* (Benin, Far North Cameroon)
**Arabian Peninsula** (the genus is present in Oman, Saudi Arabia, UAE, and Yemen)	*Achyranthesaspera* (Ghazanfar 1992); *Achyranthesaspera* s.str., A.asperavar.pubescens, A.asperavar.sicula (Miller 1996; Wood 1997); *Achyranthesaspera* s.str., A.asperavar.pubescens, *A.sicula* (Hassan et al. 2023)	*Achyranthesabyssinica*, *A.annua*, *A.aspera* s.str., *A.porphyrostachya*

From this brief overview, it is clear that the taxonomy of the *A.aspera* group is far from stable. Field observations of populations called *A.aspera* in Africa by the first author (APS) revealed striking differences, prompting us to undertake an in-depth study of this aggregate. Based on the phylogenetic paraphyly of *A.aspera*, and the previously noted variability, a morphological examination was chosen. Here, we look into the African and Arabian specimens housed in different herbaria and cited in the literature under the name *A.aspera*, including its varieties (A.asperavar.sicula, A.asperavar.pubescens), and neglected taxa described, e.g. by Moquin-Tandon (1849), Suessenguth (1938, 1952) and Suessenguth and Merxmüller (1951). In general, this aggregate includes plants with an upright stem; broad (ovate, obovate, rhombic, or elliptical) leaves; elongated spikes; glabrous, non-hooked bracteoles; and a glabrous, narrowly conical, indistinctly veined perianth that is strongly deflexed at fruiting.

Our aim is to study the African and Arabian *A.aspera* group in order to (1) provide clear morphological characters to distinct taxa, (2) resolve nomenclatural problems, and, if necessary, propose taxonomic changes, and (3) clarify the ecology and distribution of the accepted species. Although in Africa *A.fasciculata* and *A.talbotii* are morphologically distinct and do not belong to the *A.aspera* group, they will also be discussed here because of the former ambiguities.

## ﻿Material and methods

The field investigations were carried out by the first author (APS) in Zambia (2020) and Tanzania including Zanzibar (Unguja Island) and the continental part of the country (Arusha, Kilimanjaro, and Manyara Regions; 2020–2022). Ripe fruits were harvested from selected taxa for further indoor cultivation to obtain more information about the morphological variability and life form.

Taxonomic and nomenclatural synonyms were comprehensively recorded and nomenclaturally evaluated. When an infraspecific name was assigned to a higher-level infraspecific taxon in the original publication, a name of that taxon is indicated in square brackets in the nomenclatural citation. Type designations follow the requirements and procedures established in Turland et al. (2018).

Herbarium specimens were checked and determined in BM, BOL, BR, FI (incl. FI-WEBB), FT, K, LE, M, MW, MHA, MSB, RAB, W, and WU. The virtual herbaria of AMD, B, DR, CHAMB, ECWP, F, FR, G, GZU, JSB, L, MA, MAU, MPU, NY, P, S-LINN, TOG, U, UCJ, US, WAG, and Z were also checked and included in the taxonomic part with citations of the relevant specimens when the images allowed identification. We also contacted the staff of BIF and G in search for the type material of *A.frumentacea* Burm.f. Commercially distributed herbarium specimens are cited with references (in square brackets) to the published exsiccata. Herbaria codes are as per Thiers (2024).

Reproductive diaspores were photographed with a Nikon DS-Vi1 camera at the Department of Higher Plants (Moscow State University). Fruits and leaf portions were observed using scanning electron microscopy (JSM-6380LA, JEOL Ltd., Japan) at 20 kV. Prior to SEM, to restore the soft tissues, the material was dehydrated in aqueous ethyl alcohol solutions of increasing concentrations, followed by alcohol-acetone solutions and pure acetone. After critical point drying, the samples were sputtercoated with gold-palladium. Cross-sections of leaves were prepared with a sliding microtome (Microm HM 355S, Thermo Fisher Scientific, Whaltham, Massachusetts, USA) after soaking of the material in a mixture of water–alcohol–glycerin (1:1:1). After sectioning, the tissues were stained using toluidine blue.

Distribution maps were prepared using SimpleMappr online tool (http://www.simplemappr.net). For a better understanding of the distribution patterns of each species across the studied area, we provided a background map based on the major vegetation zones. The zonal patterns were compiled from different sources (Ghazanfar and Fisher 1998; Steele 2013; Dinerstein et al. 2017; Nzabarinda et al. 2021; Loidi and Vynokurov 2024), not always agreeing with each other in details, although relatively similar to the scale used here. The resulting scheme includes the following subdivisions: (1) Mediterranean zone, (2) Deserts, (3) Sahel zone, (4) Grassland and savanna, (5) Rainforest zone, and (6) South Arabian woodlands, shrublands and dunes.

The remote Chagos Archipelago is not included in the treatment due to the very limited collections available, but some specimens from this territory were revised when accessible.

## ﻿Results

**Figure 1.**
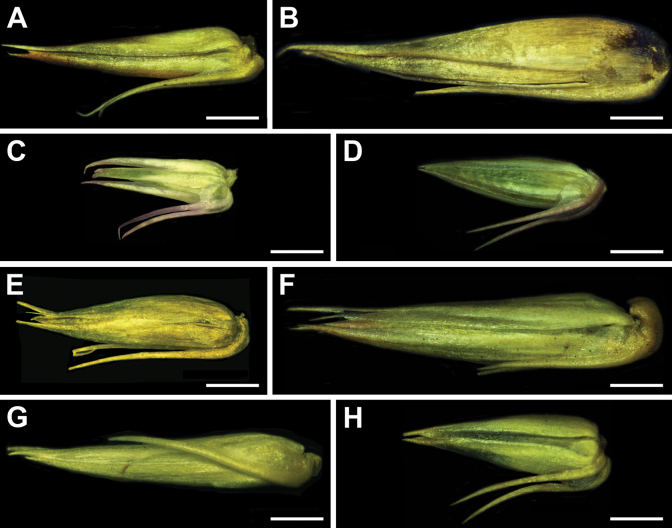
Reproductive diaspore units (perianth and two bracteoles): **A***Achyranthesabyssinica***B***Achyranthesacuminata***C***Achyranthesannua***D***Achyranthesaspera***E***Achyranthesmauritiana***F***Achyranthesporphyrostachya***G***Achyranthesseychellensis***H***Achyranthessicula*. Scale bar: 1 mm.

**Figure 2.**
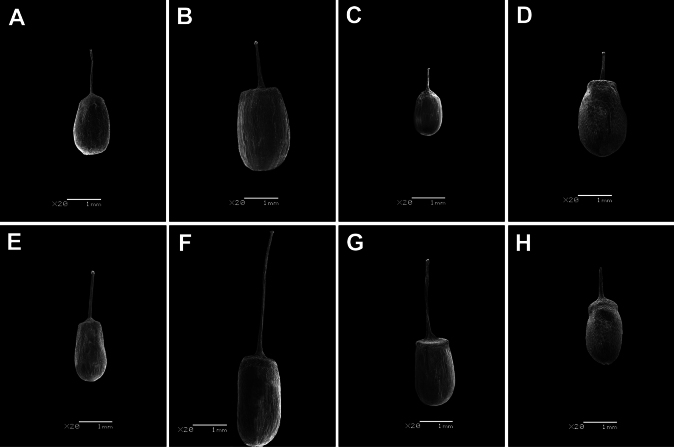
SEM images of a fruit with style and stigma: **A***Achyranthesabyssinica***B***Achyranthesacuminata***C***Achyranthesannua***D***Achyranthesaspera***E***Achyranthesmauritiana***F***Achyranthesporphyrostachya***G***Achyranthesseychellensis***H***Achyranthessicula*. Scale bar: 1 mm.

**Figure 3.**
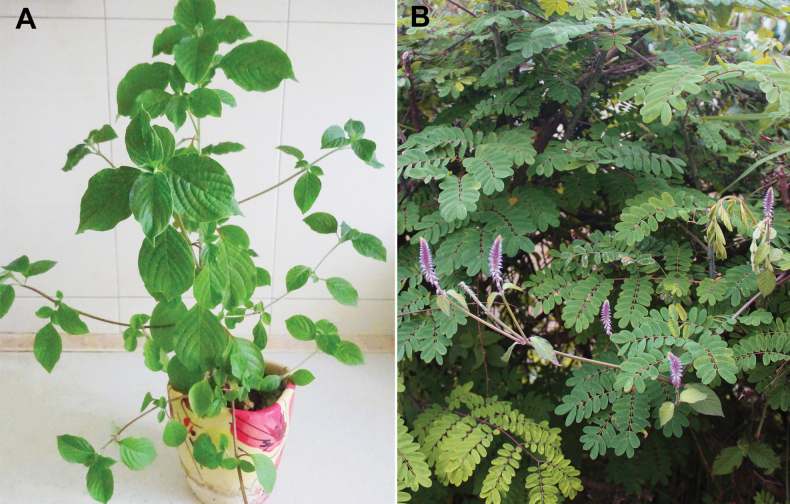
*Achyranthesabyssinica***A** an indoor cultivated plant in vegetative stage (grown from seeds from Arusha Region, Tanzania) **B** flowering twig in bushy vegetation in Meru area, Arusha Region, Tanzania. Photographer: A. Sukhorukov.

In agreement with the molecular data (Di Vincenzo et al. 2018), our morphological revision confirmed a non-uniformity of *A.aspera*, revealing eight species growing in Africa and Arabia, instead of a single variable one. Surprisingly, as a result of this study, only a small number of the African and Arabian specimens in the herbaria belong to *Achyranthesaspera* s.str. as circumscribed here, although it is commonly or frequently found in some parts of the continent, e.g. in Tropical East Africa. The majority of specimens were assigned to other taxa with different morphology, ecological preferences and geographic ranges (Table 1). Within the *A.aspera* aggregate, morphometric parameters of the reproductive structures are fundamental for identification, especially because in many cases only the upper part of a plant was collected as a herbarium voucher. The best characters to differentiate these taxa are: (1) life form, (2) perianth length (Fig. 1), (3) anther length, and (4) style length (Fig. 2). These are the characters used in our identification key. The revised circumscription gives a clearer understanding of the ecology and distributions, allowing for sustainable long-term conservation and potential use of the species.

### ﻿Key to accepted species of the *Achyranthesaspera* group occurring in Africa and Arabia

**Table:** 

1	Perianth usually 6.0–7.5(8.0) mm long	**2**
–	Perianth up to 5.0(5.5) mm long	**3**
2(1)	Annual or perennial herbs not rooting or rooting at lower nodes; leaves often nigrescent when dry; anthers 0.35–0.6 mm long; style (1.3)1.6–2.1 mm	***A.acuminata***
–	Subshrubs, not rooting; leaves not nigrescent when dry; anthers ~ 1 mm long; style (2.5)3.0–4.0 mm long	***A.porphyrostachya***
3(1)	Perianth 3.0–3.5(3.6–4.1) mm long; anthers minute, 0.15–0.25 mm long	***A.annua***
–	Perianth (3.5)4.0–5.5 mm long; anthers 0.35–1.0 mm long	**4**
4(3)	Leaf blades obovate, ovate, rhombic or elliptic, ± concolored	**5**
–	Leaf blades oblong to ovate, bicolored (green above and white or grey below)	**7**
5(4)	Anthers 0.75–1.0 mm long	***A.seychellensis***
–	Anthers up to 0.6(0.75) mm long	**6**
6(5)	Leaf blades obovate or ovate, ± hirsute on both sides, green or greyish	***A.aspera* s.str.**
–	Leaf blades rhombic or elliptic, almost glabrous, green or nigrescent	***A.mauritiana***
7(4)	Perianth (3.5)4.0–4.5(5.0) mm long; style (1.3)1.8–2.5(3.0) mm long; Tropical Africa and South Arabia	***A.abyssinica***
–	Perianth 3.5–4.0 mm long; style (1.2)1.5–1.8 mm long; North Africa	***A.sicula***

### ﻿Taxonomic treatment

#### ﻿ Achyranthes abyssinica

Taxon classificationPlantaeCaryophyllalesAmaranthaceae

Nees, Del. Sem. Hort. Vratisl. 1850: 3 (1851).

2ADB2E24-00FF-5EDD-B950-3A40C7694E01

= Achyranthesasperaf.rubella Suess., Bull. Jard. Bot. État Bruxelles 15: 56 (1938). Lectotype (designated here): DR Congo, [South Kivu Prov.,] Kizozi, 1935, *Lejeune 190* (BR0000008819512!, isolectotype BR0000008819192!).
= Achyranthesaspera [var. sicula] f. latifolia Suess., Mitt. Bot. Staatssamml. München 1: 70 (1951). Holotype: Kenya, pr. Forest Station in silva montana, 2350 m, 5 January 1922, *R.E. Fries & Th.C.E. Fries 781* (K000243724!, isotypes BR0000008819922!, S07-12310 – image!).
= Achyranthesargenteavar.albissima Suess., Mitt. Bot. Staatssamml. München 2: 70 (1955). Lectotype (designated here): Kenya, Northern Prov., Furroli [Mount], Mt. top: granite with Olea & Juniperus, 6600 ft, occasional, 20 September 1952, *J.B. Gillett 13957* (K000243722!, isolectotype K000243723!).
– Achyranthessicula auct. div.

##### Neotype (designated here).

Ethiopia, [Tigray Region,] Scholoda [Soloda Mt. near Adwa Town], 12 November 1838, *Schimper 1144* [Schimper, Iter Abyssinicum, ser. 2 (1842)] (BM!, isoneotypes G00688998, BR00000083356949!, FI!, K005771057! – central twig, LE!, M0241524!, WU!).

##### Description.

(Fig. 3). Annual, stout or rarely delicate herb up to 1.0(1.5–2.0) m, not rooting at nodes, very rarely creeping at lower nodes; stem four-angled, sparsely pubescent, sometimes scrambling; lateral branches long and ± horizontally spreading; leaf petioles 10–25 mm long, blades 20–100(120) × 10–40(50) mm, broadly cuneate or truncate, ovate, entire, tip acuminate, bicolored: ± green, slightly to moderately pubescent above, whitish or grey below (sparsely pubescent and green when cultivated indoor); inflorescence 50–250 mm long, sometimes pendulous; bract ~2.0 mm long; bracteoles 2.5–3.0 mm long, reflexed; perianth (3.5)4.0–4.5(5.0) mm long, green or purplish inside; pseudostaminodes 0.6–1.0 mm long, short, white or pink, fimbriate or not; stamens 5, with pink filaments, anthers 0.4–0.7 mm long, pink or magenta; style (1.3)1.8–2.5(3.0) mm long, pink; fruit (without style) (1.3)1.8–2.5(3.0) mm long.

##### Taxonomic notes.

Morphologically, *A.abyssinica* is similar to *A.sicula* but differs from the latter by a slightly longer perianth and style, although a few specimens from tropical Africa have the same style length as in *A.sicula*. Although these two species are very similar in morphology, as also shown in our identification key, they take different positions in the molecular phylogeny (unpublished). Both species have clearly different and non-overlapping distributions, with *A.abyssinica* growing in mountainous areas of East and Central Africa and South Arabia, whereas *A.sicula* is a subtropical species found in areas with a Mediterranean climate.

##### Nomenclatural notes.

Unfortunately, the whereabouts of the type specimens of *Achyranthesabyssinica* in the Nees herbarium are mostly unknown because this collection had been split into numerous parts, which were sold to institutions or private individuals (Stafleu and Cowan 1981). The contents of these parts are not always certain; besides, the collection was heavily affected by extensive losses in German herbaria during the Second World War. Our request to Bonn, where these type specimens were possibly kept, was unsuccessful because that Herbarium was completely destroyed during the war (Thomas Joßberger, pers. comm.). There are also no authentic specimens in STR (Gisele Haan-Archipoff, pers. comm.) and LE (Sukhorukov, pers. obs.), where large parts of Nees collections are housed.

In the absence of any original material, we designate Schimper’s collection from Ethiopia, which was prepared in numerous duplicates and commercially distributed to many Herbaria under the name *A.argentea*, as the neotype of *A.abyssinica*.

The label of the isolectotype of A.asperaf.rubella Suess. gives no exact location information, but both specimens belong to a single gathering (*Lejeune 190*). No other specimens of the original material are known, and the type choice is therefore rather formal.

Suessenguth in Suessenguth and Merxmüller (1951) cited the type of A.asperaf.latifolia Suess. as kept at Kew. The duplicates in BR and S are, therefore, isotypes.

Achyranthesargenteavar.albissima Suess. comprises mountainous plants with small white leaves. Suessenguth in Suessenguth and Merxmüller (1951) designated two specimens of a single gathering at Kew as “typus”. These specimens are syntypes; a lectotype is designated here to restrict the typification to a single specimen.

##### Habitat.

Open or slightly shady grassy slopes, bushy vegetation, calcareous outcrops, sometimes as a weed, at elevations of 1000–3500 m a.s.l. in arid or seasonally moist areas.

##### Distribution.

**Figure 4.**
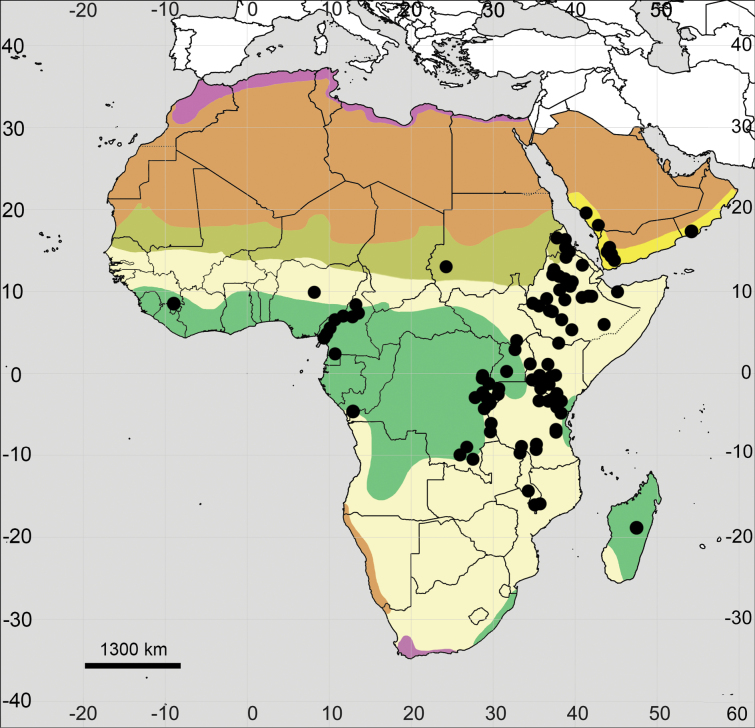
Distribution map of *Achyranthesabyssinica*.

(Fig. 4; see also Appendix 1). **Africa**: Angola, Burundi, Cameroon, DR Congo, Eritrea, Ethiopia, Guinea, Kenya, Madagascar, Malawi, Nigeria, Rwanda, Somalia, South Sudan, Sudan, Tanzania, Uganda.

**Arabian Peninsula**: Oman, Saudi Arabia, Yemen.

##### General distribution.

The species is only known from tropical Africa and Arabia.

#### ﻿ Achyranthes acuminata

Taxon classificationPlantaeCaryophyllalesAmaranthaceae

E.Mey. ex Sonder, Linnaea 23: 96 (1850).

FCDE0DE6-BCD3-53D4-A285-C4E7776D3297

= Achyranthesasperaf.nigrescens Suess., Bull. Jard. Bot. État Bruxelles 15: 56 (1938). Lectotype (designated here): DR Congo, [Équateur Prov.,] Environs d’Eala, July 1930, *J. Lebrun 1230* (BR0000008819543!, isolectotypes BR000000881923!, K!).
– Achyranthesasperavar.rubrofusca auct. in herb. BM, K.
– Achyranthesbidentatavar.africana Cavaco, nom. nud. in herb. BM, K.
– Achyranthesbidentata auct.: Baker and Clarke (1909), Hutchinson and Dalziel (1927), Keay (1954), Lebrun and Stork (1991), Figueiredo and Smith (2008), Phiri (2005), Klopper et al. (2006).
– Achyranthesafricana Sukhor., nom. nud. in herb. BM, BR, K.

##### Lectotype (designated here).

South Africa, Port Natal [Durban], schattige Waldplätze, 400 ft, 9 April 1832, *Drège 4679* (P01029520 – image!).

##### Description.

**Figure 5.**
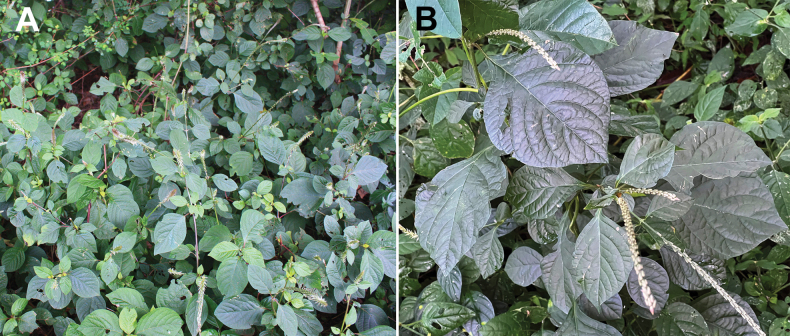
*Achyranthesacuminata***A** general view of the plants (Meru area, Arusha Region, Tanzania. Photographer: A. Sukhorukov) **B** close-up of the leaves and inflorescences (KwaZulu-Natal Province, near Durban, South Africa. Photographer: Errol Douwes).

**Figure 6.**
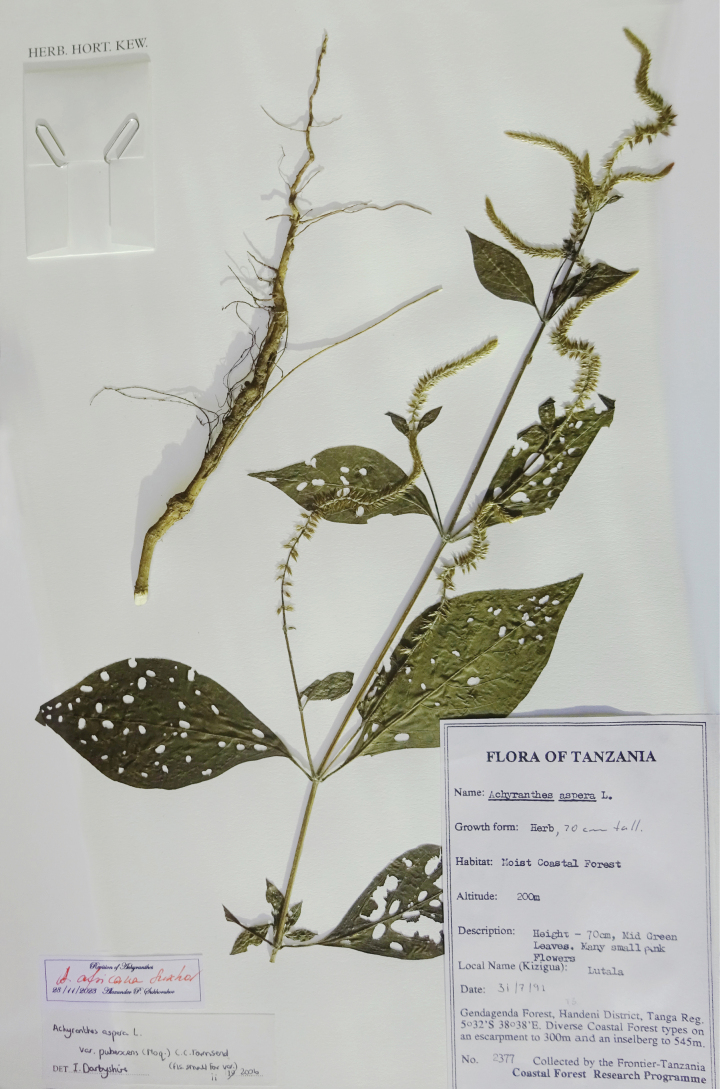
A herbarium specimen of *Achyranthesacuminata* (Tanzania, Tanga Region, Gendagenda Forest, 200 m a.s.l., 31 Jul 1991, *Frontier Tanzania team* 2377, K).

(Figs 5, 6). Annual or short-lived perennial herb up to 1.5(2.0) m tall occasionally creeping and rooting at lower nodes; stem sparsely pubescent, obscurely quadrangular turning angular in the inflorescence; leaf petioles 10–40(60) mm, blades 30–260 × 20–110 mm, cuneate, ovate or rhombic or even obovate, entire, tip short or long acuminate, deep green or olivascent and almost glabrous above, pale green or purplish and slightly to moderately pubescent below, usually turning blackish when dry; inflorescence 80–270 mm long, dense, paracladies always present, in early stages thin and delicate similar to inflorescences of some willows (e.g. *Salixtriandra*); bract 3.0–4.0 mm long; bracteoles (3.0)4.0–5.0 mm long, reflexed; perianth segments slightly unequal, (4.5–5.0)6.0–7.0(8.0) mm long (two segments longer than three others), green outside and purple-red inside at least at the tip, turning dark brown when dry, three inner segments keeled; pseudostaminodes brownish (when dry), 0.8–1.1 mm, not or slightly fimbriate; stamens 5, filaments purple, anthers 0.35–0.6 mm long, magenta; style (1.3)1.6–2.1 mm long, pink or mauve; fruit (without style) 1.7–2.5 mm long.

##### Taxonomic note.

For a long time, specimens of *A.acuminata* were identified as *A.aspera* or its varieties, e.g. A.asperavar.rubrofusca in the DR Congo, or as A.asperavar.nigrescens (Suessenguth in Suessenguth and Merxmüller 1951), and, in some cases in tropical Africa, as *A.bidentata* (e.g., Baker and Clarke 1909; Hutchinson and Dalziel 1927; Townsend 1973; Lebrun and Stork 1991; Klopper et al. 2006). Nonetheless, Townsend (1985) claimed that the Asian *A.bidentata* did not occur in East Tropical Africa and referred specimens of *A.acuminata* to A.asperavar.pubescens (Moq.) C.C.Towns. Lebrun and Stork (2003) vaguely discussed some morphological differences between *A.aspera* s.l. and *A.bidentata* from Asia and Africa, concluding that there are differences between both species as well as differences between Asian and African populations of *A.bidentata*.

*Achyranthesacuminata* differs from the Asian *A.bidentata* and *A.japonica* (Miq.) Nakai by its obovate leaves turning blackish when dry (facultative character) and longer (1.6–2.1 mm) styles. The two Asian species are rhizomatous perennial plants with ovate leaves, which do not turn black when dry, and have shorter styles (1.0–1.5 mm long). To date, the presence of any species of the Asian *A.bidentata* group is not confirmed in Africa, and the native distribution of this group of species is restricted to tropical and warm temperate Asia (South and East China, Japan, South-East Asia, and countries located in the Himalayas). *Achyranthesjaponica* is reported as an alien and naturalized plant in temperate North America (Medley et al. 1985).

Achyranthesasperavar.rubrofusca was erroneously reported from Africa (Suessenguth in Suessenguth and Merxmüller 1951). Its basionym *A.rubrofusca* (Wight 1852) was described from India and belongs to the *A.bidentata* group.

##### Nomenclatural notes.

The name *A.acuminata* was only mentioned (as *nomen nudum*) in Drège (1843), who cited a number of plant species (incl. *A.acuminata*) growing in forests and woodlands (‘Wälder und Holzungen’), and it was later validated by Sonder (1850). Cavaco (1962: 127) considered *A.acuminata* as a putative synonym of the subshrubby A.asperavar.porphyrostachya (≡ *A.porphyrostachya*) probably based on similar leaf shape. Authentic specimens of *A.acuminata* have obovate leaves, different from *A.porphyrostachya*, which has oblong or ovate leaves. Moreover, *A.acuminata* and *A.porphyrostachya* can be separated based on their ecology.

An authentic specimen of *A.acuminata* kept at W ([without exact location and date] E. M.[eyer] (W18393!) has a perianth shorter (4.5–5.0 mm long) than usual (6.0–7.0 mm). A shorter perianth length was also observed in specimens from dryer areas (e.g., growing in Ethiopia and Benin). On the other hand, the length of anthers (±0.5 mm long) and style (±1.8 mm) long is constant in the species.

Suessenguth (1938) cited two specimens of A.asperaf.nigrescens Suess., which were collected in the Belgian Congo [now DR Congo]: Matadi [Kongo Central Prov.] and Eala [Équateur Prov.]. Both specimens represent the same species. Suessenguth was probably the first to mention its remarkable character: leaves turning black when dry.

##### Habitat.

Riverine and primary rain forests, forest margins and other wet shady places at elevations of 0–2000 m a.s.l.

##### Distribution.

**Figure 7.**
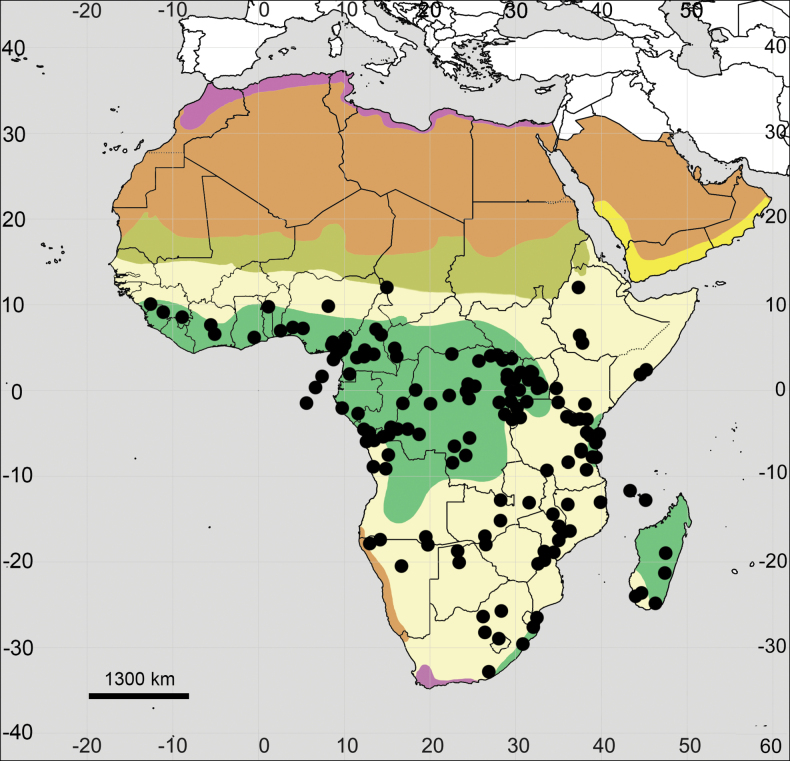
Distribution map of *Achyranthesacuminata*.

(Fig. 7; see also Appendix 1). **Africa**: Angola, Benin, Botswana, Burundi, Cameroon, Central African Republic, Comoros, Congo Republic, DR Congo, Equatorial Guinea, Ethiopia, Gabon, Guinea, Ivory Coast, Kenya, Lesotho, Madagascar, Malawi, Mayotte, Mozambique, Namibia, Nigeria, Rwanda, São Tomé and Príncipe, Sierra Leone, Somalia, South Africa, Tanzania, Togo, Uganda, Zambia, Zimbabwe.

##### General distribution.

Tropical Africa.

#### ﻿ Achyranthes annua

Taxon classificationPlantaeCaryophyllalesAmaranthaceae

Dinter, Repert. Spec. Nov. Regni Veg. 15: 82 (1917).

65E022BD-1675-578C-9436-153FC9955055

≡ Achyranthesargenteavar.annua (Dinter) Suess., Mitt. Bot. Staatssamml. München 1: 152 (1952).
≡ Achyranthesaspera [var.sicula] f.annua (Dinter) Cavaco, Mém. Mus. Nat. Hist. Nat. 13, ser. B (Botanique): 122 (1962).
= Achyranthesargenteavar.viridescens Moq. in DC., Prodr. 13(2): 315 (1849). Lectotype (designated here): [Egypt] Cairo, Rudach [Roda] Island, [1818], *F.W. Sieber s.n*. (G-DC [image!]; isolectotypes M0241528!, M0241529!, K! [left-hand specimen, mounted together with A.sicula from Tanger, Morocco]).
= Achyranthesasperoides Pires de Lima, Brotéria. Sér. Bot. 19: 116 (1921). Lectotype (designated here): Mozambique. Na planície inculta junto de Palma, 26 February 1917, A. Pires de Lima 118 (PO69282 – image!; isolectotype PO69283 – image!).
= Achyranthesasperaf.annulosa Suess., Mitt. Bot. Staatssamml. München 1: 69 (1951). Holotype: Uganda, Ishasha, 4000 ft, November 1946, *J.W. Purseglove 2284* (K!).
= Achyranthesaspera [f.annulosa] subf.angustifolia Suess., Mitt. Bot. Staatssamml. München 1: 69 (1951). Holotype: Sierra Leone, [Southern Prov.,] Njala, 28 December 1932, *F.C. Deighton 2584* (K000243721!).
– Achyranthesargentea auct.: Berhaut (1971).
– Achyranthesasperavar.argentea auct.: Hauman (1951), Cavaco (1953), Sunding (1973).
– Achyranthesaspera auct. in herb. div.
– Achyranthesasperavar.sicula auct.: Boudet and Lebrun (1986), Barry and Celles (1991), Lebrun et al. (1991), Audru et al. (1994), Townsend (1985, 1988, 1993b, 2000), Miller (1996), Wood (1997), Germishuizen and Meyer (2003), Mapaura et al. (2004), Setshogo (2005), Figueiredo and Smith (2008), Brundu and Camarda (2013), Klaassen and Kwembeya (2013), César and Chatelain (2019), Odorico et al. (2022), Gosline et al. (2023).
– Achyranthessicula auct.: Podlech (1966), Lebrun (1973), Peyre de Fabregues and Lebrun (1976), Hassan et al. (2023).

##### Type.

**Figure 8.**
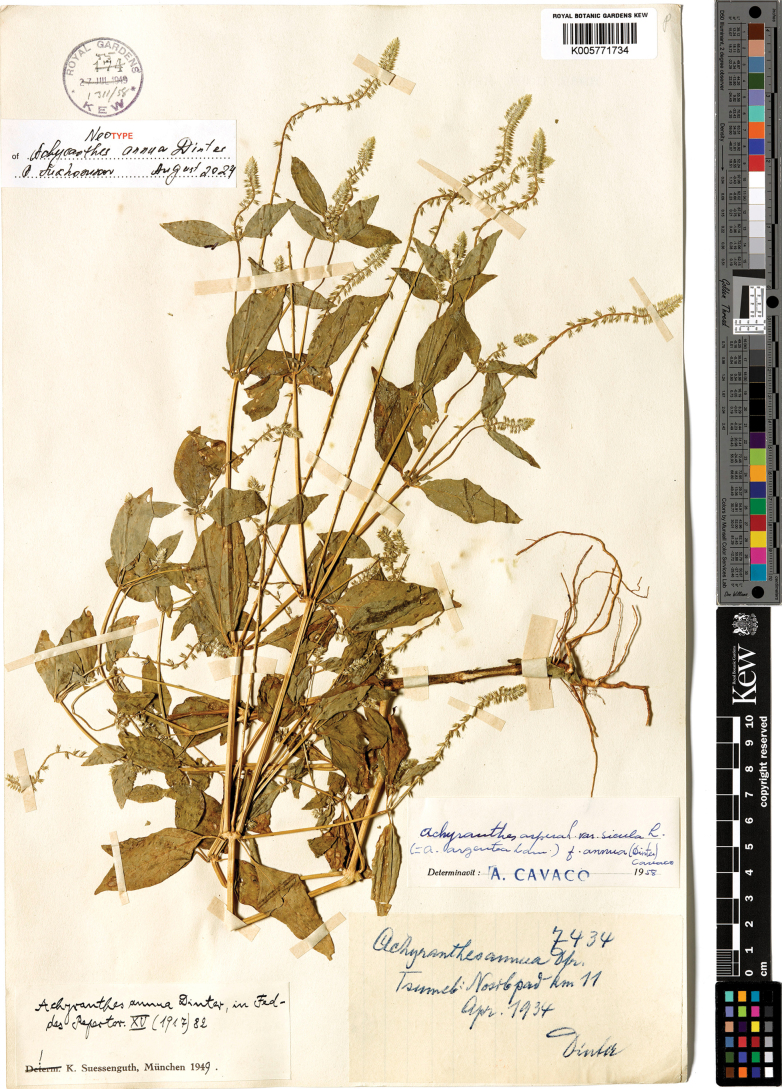
Neotype of *Achyranthesannua* (Namibia, Oshikoto Region, Tsumeb, April 1934, *Dinter 7434*, K005771734). The image was provided by RBG Kew (available at http://specimens.kew.org/herbarium/K005771734).

Namibia, [Otjozondjupa Region] Otjihua b.[y] Okandja Dtr. [District] Eahero [Farm], *Dinter 3303* (B, destroyed). ***Neotype*** (selected here): Namibia, [Oshikoto Region] Tsumeb, April 1934, *Dinter 7434* (K005771734!). Fig. 8.

##### Description.

**Figure 9.**
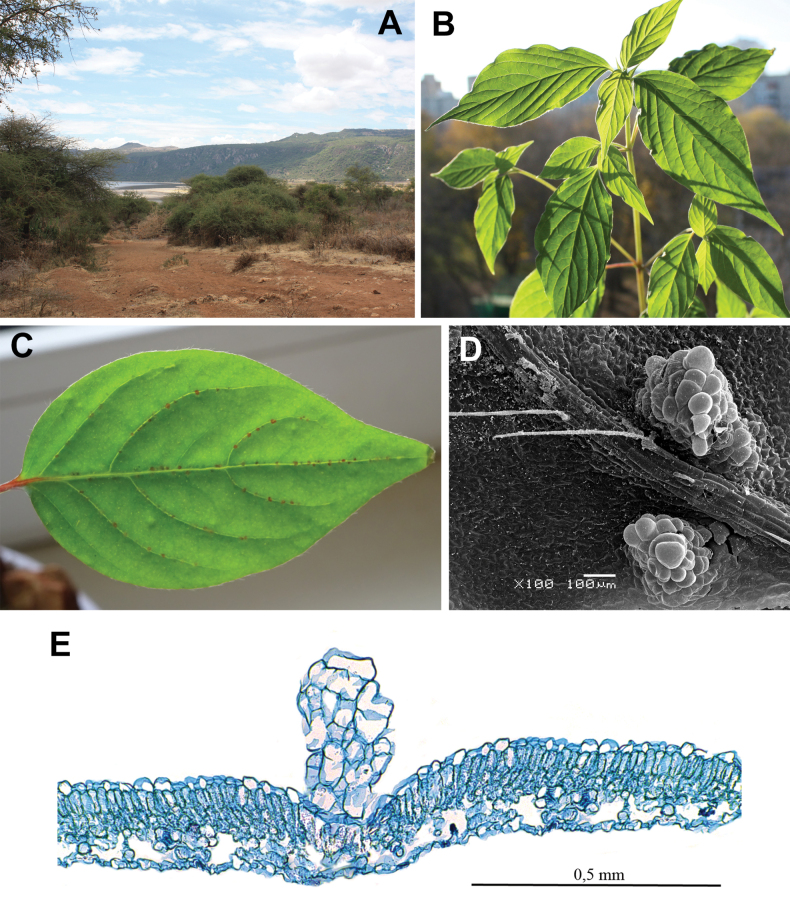
*Achyranthesannua***A** typical habitat with *Vachellia* trees, Manyara Region, Tanzania **B** an indoor cultivated plant in vegetative stage (grown from seeds from Manyara Region, Tanzania) **C** adaxial side of the leaf with red gland-like outgrowths on the veins (indoor cultivated plant) **D** close-up view of gland-like outgrowths and simple hairs **E** cross-section of a leaf showing bifacial anatomy and structure of an outgrowth. Scale bars: 100 µm (**D**); 0.5 mm (**E**). Photographers: **A–D**: A. Sukhorukov, **E** M. Nilova.

(Fig. 9). Annual, rather delicate herb, 20–100(150) cm tall, not rooting; stems four-angled; lateral branches at acute angle with the main axis; leaf petioles 10–30 mm long, blades 20.0–80.0 × 10.0–60.0 mm, ovate, base truncate, tip ± attenuate, green, often turning red later, sometimes gray or pale green abaxially because of abundant silvery and appressed hairs, pendulous at night (at least in cultivation indoors); young leaves often with small red gland-like outgrowths on the veins adaxially (Fig. 9C–E) turning yellow and then pale white (indoor cultivated plants); inflorescence with ± equal main and lateral branches, with fruits rather distant at least in lower part; bract 1.5–2.0 mm long, bracteoles 2.0–3.0 mm long, pinkish, reflexed; perianth 3.0–3.5(3.6–4.1) mm long, segments ± equal, one-veined with indistinct two lateral veins; stamens 5 with white or mauve filaments, anthers 0.15–0.25 mm long, yellow or pink, pseudostaminodes 0.5–1.2 mm long, apically ± fimbriate, white; style with stigma 0.5–0.8(1.0) mm long; fruit (without style) 1.0–1.5(1.8) mm long.

##### Taxonomic note.

Dinter (1917) described the species from Namibia and correctly noted that there are no intermediates between *A.aspera* and *A.annua*. However, the characters mentioned in the protologue – life form and inflorescence length – cannot be considered as delimiting traits. Both *A.annua* and *A.aspera* are annuals, although the latter species often resembles a perennial herb reaching 2 m tall and having long (up to 70 cm) inflorescences under favorable conditions (e.g., in a humid climate with enough precipitation). Habitually, *A.annua* is similar to *A.abyssinica* and *A.sicula* but differs by the minute anthers and much shorter styles. In addition, *A.annua* has leaves usually green on both sides, but sometimes their abaxial surface may be pale green or gray due to abundant simple hairs, especially in the populations growing in the Desert and Sahel zones. The name *Achyranthesannua* was not retained after its description (Dinter 1917: 82). Podlech (1966) synonymized it with *A.sicula*, mentioning that *A.sicula* in Namibia is an annual plant. The recent checklist of Namibian plants (Klaassen and Kwembeya 2013) did not include *A.annua*, with only a single species with two varieties listed (A.asperavar.asperaandvar.sicula). Our study reveals that *A.annua* is not only present in Namibia but is widely distributed across semi-arid regions of Africa reaching up to the Arabian Peninsula.

The gland-like outgrowths on the leaves (Fig. 9C) were detected only in some individuals of *A.annua* cultivated indoors; they were not observed in other species of the genus.

##### Nomenclatural notes.

The type of *Achyranthesannua*, previously kept at B, was lost during the Second World War, and no other original material has been traced in other herbaria visited or digitized. There are no duplicates of *A.annua* in the HBG herbarium, which contains many duplicates of K. Dinter (pers. comm. M. Schultz). Nevertheless, the first author (APS) has found a specimen collected and labelled by K. Dinter in K, which is selected here as a neotype.

Moquin-Tandon (1849) cited two gatherings of A.argenteavar.viridescens Moq. (“prope Catanam”, Heldreich; “in Aegypto”, Sieber) in personal collections of Poiret and Candolle, which are, therefore, syntypes. A specimen collected by Sieber and kept at G-DC is designated as lectotype here. This specimen was collected in 1818, when Sieber travelled across Crete, Egypt and Palestine (Sieber 1823), and commercially distributed within a set of dried specimens of 230 species in 1819 (Sieber 1821).

Pires de Lima (1921) cited a single gathering in the protologue of *A.asperoides*, *Pires de Lima 118*, but the original material also comprises an uncited gathering, *Pires de Lima 306*, which was collected on another date. The cited collection is therefore not the holotype but a syntype, and lectotypification is warranted in this case. The plants collected are rooting at the lower nodes, with long lateral inflorescence’s branches. Such specimens are sometimes present in *A.annua*, but the exact measurements of reproductive characters are not known yet. In any case, the species cannot be synonymized with *A.acuminata*, *A.aspera*, or *A.porphyrostachya*.

##### Habitat.

A typical shade-loving plant usually collected in *Vachellia*–*Commiphora* bushlands (in East Africa), sometimes in rocky, sandy, volcanic habitats or in degraded plant communities in drier regions at elevations of 0–2400 m a.s.l. Dinter (1917) also indicated that *A.annua* is a common plant in under the canopy of *Vachellia* bushlands in SW Africa [Namibia]. N.M. Otto (in labels at herb. M) noted that *A.annua* (as *A.aspera*) is also common in KwaZulu-Natal (South Africa) as a weed in cultivated areas, where the farmers called it “wild buckwheat”. Reports of the common presence of *A.aspera* in subcanopy communities in the tropical part of South Africa (Leistner 1996) should probably belong to *A.annua*. Plants of this species are eaten by livestock.

##### Distribution.

**Figure 10.**
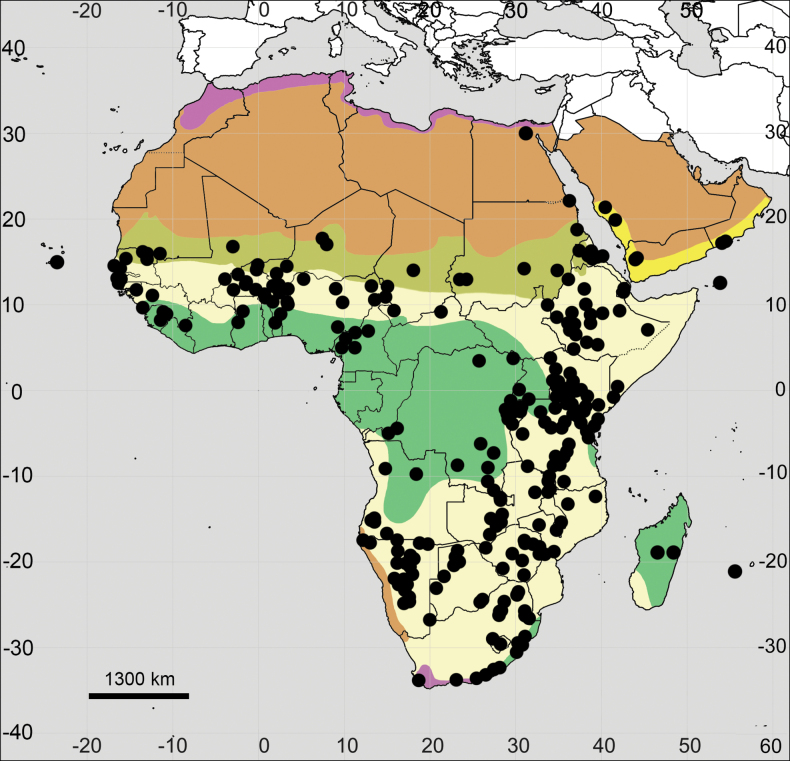
Distribution map of *Achyranthesannua*.

(Fig. 10; see also Appendix 1). **Africa**: Angola, Benin, Botswana, Burkina Faso, Burundi, Cameroon, Cape Verde, Central African Republic, Chad, Djibouti, DR Congo, Egypt, Eritrea, Eswatini, Ethiopia, Gambia, Ghana, Guinea, Guinea-Bisau, Kenya, Lesotho, Liberia, Madagascar, Malawi, Mali, Mauritania, Mozambique, Namibia, Niger, Nigeria, Réunion, Rwanda, Senegal, Sierra Leone, Somalia, South Africa, South Sudan, Sudan, Tanzania, Togo, Uganda, Zambia, Zimbabwe.

**Arabian Peninsula**: Oman, Saudi Arabia, Yemen.

##### General distribution.

Africa and Arabia.

#### ﻿ Achyranthes aspera

Taxon classificationPlantaeCaryophyllalesAmaranthaceae

L., Sp. Pl. 1: 204 (1753) nom. cons.

FF1200EC-C3F9-52A8-A4EE-2C7DC0A7C2F0

≡ Achyranthesasperavar.indica L. Sp. Pl. 1: 204 (1753).
≡ Achyranthesindica (L.) Mill., Gard. Dict., ed. 8: [unpaged] Achyranthes no. 2 (1768).
≡ Cadelariaindica (L.) Raf., Fl. Tellur. 3: 45 (1837).
≡ Stachyarpagophoraaspera (L.) M.Gómez, Anales Inst. Segunda Enseñ. 2: 312 (1896).
≡ Centrostachysaspera (L.) Standl., J. Wash. Acad. Sci. 5: 75 (1915).
≡ Centrostachysindica (L.) Standl., J. Wash. Acad. Sci. 5: 75 (1915).
= Achyranthesobtusifolia Lam., Encycl. 1(2): 545 (1785).
≡ Achyranthesasperavar.obtusifolia (Lam.) Suess., Mitt. Bot. Staatssamml. München 1(5): 152 (1952). Lectotype (designated here): [India?] leg. *Sonnerat s.n*. (P-LA00380993, image!).
= Achyranthesargenteavar.obovata Moq. in DC., Prodr. 13(2): 316 (1849). Holotype: [Saudi Arabia] “Locis cultis ad pagum Madara vallis Fatme prope Meccam”, 24 November 1833, *W. Schimper 944* [Unio itineraria (1837)] (G00688924!, image available at http://www.ville-ge.ch/musinfo/bd/cjb/chg/adetail.php?id=668392&base=img&lang=en); isotypes HBG503188, M0241524!).
= Achyranthesasperavar.simplex Millsp., Publ. Field Columb. Mus., Bot. ser. 2: 36 (1900). Holotype: [US Virgin Islands] St. Thomas, Charlotte Amalie, 17–18 January 1899, *C.F. Millspaugh 484* (F – image!, isotype NY658322 – image!).
= Achyranthesobovata Peter, Repert. Spec. Nov. Regni Veg. Beih. 40(2, Anhang): 25 (1932) nom. illeg., non (M.Mart. & Gal.) Standl. (1915). Holotype: Deutsch-Ostafrika [Tanzania, Tanga Region] West-Usambara, Mashewa, Gegend Tunya und Kwatangu, 400 m, 8–9 September 1915, A. Peter 13869 (B – image!).

##### Lectotype (designated by Townsend 1974).

[Sri Lanka] Herb. Hermann 2: 69, no. 105 (BM000621744!).

##### Description.

**Figure 11.**
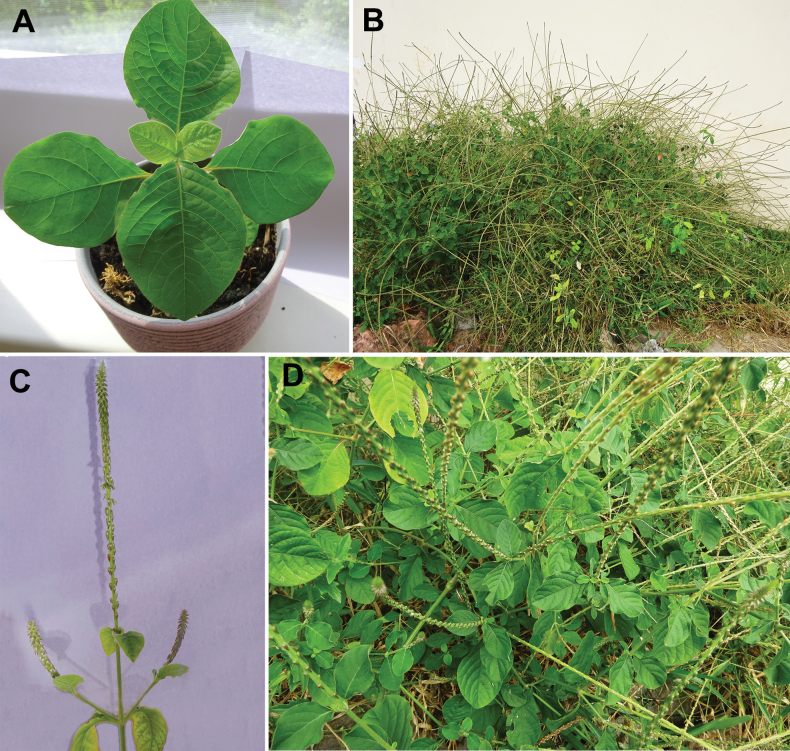
*Achyranthesaspera***A** an indoor cultivated plant in vegetative stage (grown from seeds from Unguja Island, Tanzania) **B** two bushy plants in fruiting stage (disturbed habitat on Unguja Island, Tanzania) **C** inflorescence of an indoor cultivated plant at fruiting **D** close-up view of a fruiting plant (disturbed habitat on Unguja Island, Tanzania). Photographer: A. Sukhorukov.

(Fig. 11). Annual herb, 30–200 cm tall, stout, not rooting; young stem round, angled at the inflorescence; leaf petioles 10–30 mm, blades 15–110 × 20–80 mm, obovate or ovate, base truncate, pubescent throughout, tip shortly acuminate; inflorescence up to 70 cm long; bract 2.0–2.5 mm long; bracteoles 2.5–3.5 mm long, green, not or slightly reflexed; perianth 4.0–4.5 mm long, segments ± equal, one-veined with two indistinct lateral veins; stamens 5 with white or mauve filaments, anthers 0.4–0.6 mm long; pseudostaminodes 0.7–1.1 mm long, white; style with stigma 1.0–1.1 mm long; fruit (without style) 1.8–2.2 mm long.

##### Taxonomic note.

Many authors (Podlech 1966; Berhaut 1971; Townsend 1980b; Ghazanfar 1992; Germishuizen and Meyer 2003; De Lange et al. 2004; Gibreel and Darbyshire 2015) indicated that *A.aspera* is a perennial herb occasionally flowering in the first year, but our field observations revealed that the species could be mistaken for a perennial based on its height and much branched habit in the moist regions (e.g., Zanzibar, Tanzania; Haryana, India: pers. obs. APS), but is a robust annual.

##### Nomenclatural notes.

The lectotype of *Achyranthesaspera* designated by Townsend (1974) and proposed for conservation by Jarvis (1992), although only represented by the upper part of a plant, clearly shows the characters typical of the taxon (obovate leaves, short bracteoles and flowers). Fawcett and Rendle (1914: 136) indicated that the type of A.asperavar.indica is kept at the Natural History Museum (BM) and referred to Hermann’s Flora Zeylanica. This choice was formalised by Townsend (1985), who, therefore, made this varietal name a homotypic synonym of the type variety. Jarvis (2007) disagreed with Townsend (1985) and noted that Burman (1737) is the nomenclatural source of this Linneaen variety and should provide a type. Iamonico (2014) selected a lectotype of A.asperavar.indica in the herbarium S-LINN (IDC 100.19) that is annonated by Linnaeus as “β *aspera*”. This sheet contains a fragment of the plant with three obovate leaf pairs and an inflorescence in early blooming stage. We have doubts whether this plant is the annual *A.aspera*, and we assume that the specimen in S-LINN rather belongs to *A.porphyrostachya* (although its leaf shape is not characteristic for the latter species). Despite these doubts, the protologue of A.asperavar.indica (Linnaeus 1753) contains a separate diagnosis plus the indication that the variety occurs in “India” (“Zeylona” = Sri Lanka). This information links the Hermann’s specimen with the protologue and makes it part of the original material, thus eligible for lectotypification. For this reason, the type designation made by Townsend (1985) is formally correct and has priority over Iamonico’s choice; it stabilises the nomenclature of *A.aspera* as established by Townsend (1974, 1985) and universally accepted.

The protologue of *A.obtusifolia* (Lamarck 1785) includes a number of cited illustrations which constitute its original material together with the specimens in the personal collection of Lamarck. A lectotype is therefore designated here.

Hassan et al. (2023) synonymized A.argenteavar.obovata Moq. with *A.sicula*. Nevertheless, the holotype represents a branched specimen of *A.aspera* s.str. This lectotype is the only specimen used for and cited in the protologue (Moquin-Tandon 1849), which is thus a holotype by definition; the lectotypification with the same specimen made by Hassan et al. (2023) was therefore superfluous.

The holotype of A.asperavar.simplex Millsp. is an unbranched specimen with a terminal inflorescence; its leaves are very typical of *A.aspera*.

##### Habitat.

Waste, mostly sunny places; frequent in the regions with humid climate (e.g., Unguja Island, Tanzania) at elevations of 0–1500 m a.s.l.

##### Distribution.

**Figure 12.**
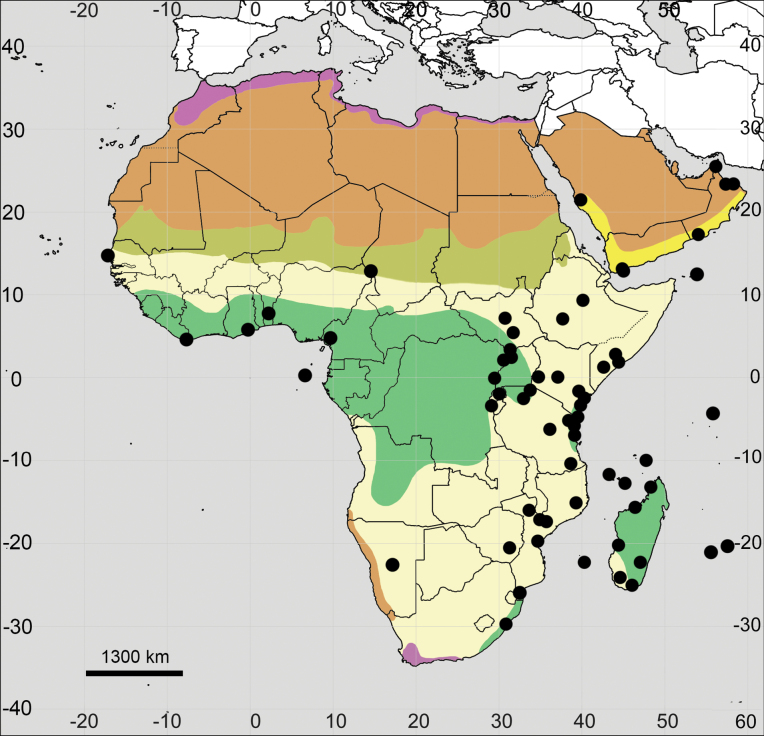
Distribution map of *Achyranthesaspera*.

(Fig. 12; see also Appendix 1). Surprisingly, herbarium collections from the western part of Africa are scarce, indicating that it might be a scattered alien in the West Tropical Africa. The majority of collections are from the eastern tropical part of Africa and the islands in the western Indian Ocean, where it is usually considered as alien.

**Africa**: Benin, Cameroon, Chad, Comoros, DR Congo, Egypt (old record), Europa Island, Ethiopia, Ghana, Kenya, Liberia, Madagascar, Mauritius, Mayotte, Mozambique, Namibia, Réunion, Rwanda, São Tomé and Príncipe, Senegal, Seychelles, Somalia, South Africa, South Sudan, Tanzania, Uganda, Zimbabwe.

**Arabian Peninsula**: see Appendix 1.

##### General distribution.

The exact origin of the species is unknown, but it seems to be native in tropical South and South-West Asia. Because of the confusion of *A.aspera* s.str. with other species and varieties, we have detailed its distribution across the World based on the specimens seen in the herbaria visited and online collections.

**Africa**: see Appendix 1.

**Asia**: Bangladesh, Bhutan, Cambodia, tropical China including Hong Kong and Taiwan, India, Indonesia, Laos, Malaysia, Maldives, Myanmar, Nepal, Oman, Pakistan, Philippines, Saudi Arabia, Singapore, Sri Lanka, Thailand, UAE, Vietnam, Yemen (incl. Socotra).

**Australasian Realm**: tropical Australia, Papua New Guinea.

**Melanesia**: New Caledonia (probably other islands).

**Polynesia**: Pitcairn Island, Samoa. Also reported for many other islands of Polynesia (Florence 2004).

**America**: Antigua and Barbuda, Bahamas, Barbados, Bolivia, British Virgin Islands, Brazil (tropical regions, e.g. States of Bahia, Espírito Santo, Minas Gerais, Pará), Colombia, Costa Rica, Cuba, Cayman Islands, Dominica, Dominican Rep., French Guiana, Grenada, Guadeloupe, Guatemala, Guyana, Haiti, Jamaica, Kingdom of the Netherlands (Aruba, Bonaire, Curaçao, St.-Eustatius, St.-Martin), Martinique, Mexico, Montserrat (British Overseas Territory), Nicaragua, Panama, Puerto Rico, Salvador, St. Barthélemy (French Overseas Territory), St. Kitts and Nevis, St. Lucia, St. Vincent and the Grenadines, Suriname, Trinidad and Tobago, USA (Alabama, Florida, Louisiana, Texas, and Hawaii), US Virgin Islands.

It is common or frequent in disturbed places at lower altitudes in tropical America (e.g., Millspaugh 1900, as *A.asperaobtusifolia*; Robertson 1981; Acevedo-Rodríguez and Strong 2012; pers. obs. of APS in Grenada in 2016).

#### ﻿ Achyranthes mauritiana

Taxon classificationPlantaeCaryophyllalesAmaranthaceae

Moq. in DC., Prodr. 13(2): 313 (1849).

7DA71355-D55D-5B14-B346-A5210CE0F8C1

##### Lectotype (designated here).

Mauritius, [1768–1773], [*Commerson*] *s.n*. (P00487020 [image!]; isolectotypes FI-WEBB154894!, G-DC G00688999 [image!]).

##### Description.

**Figure 13.**
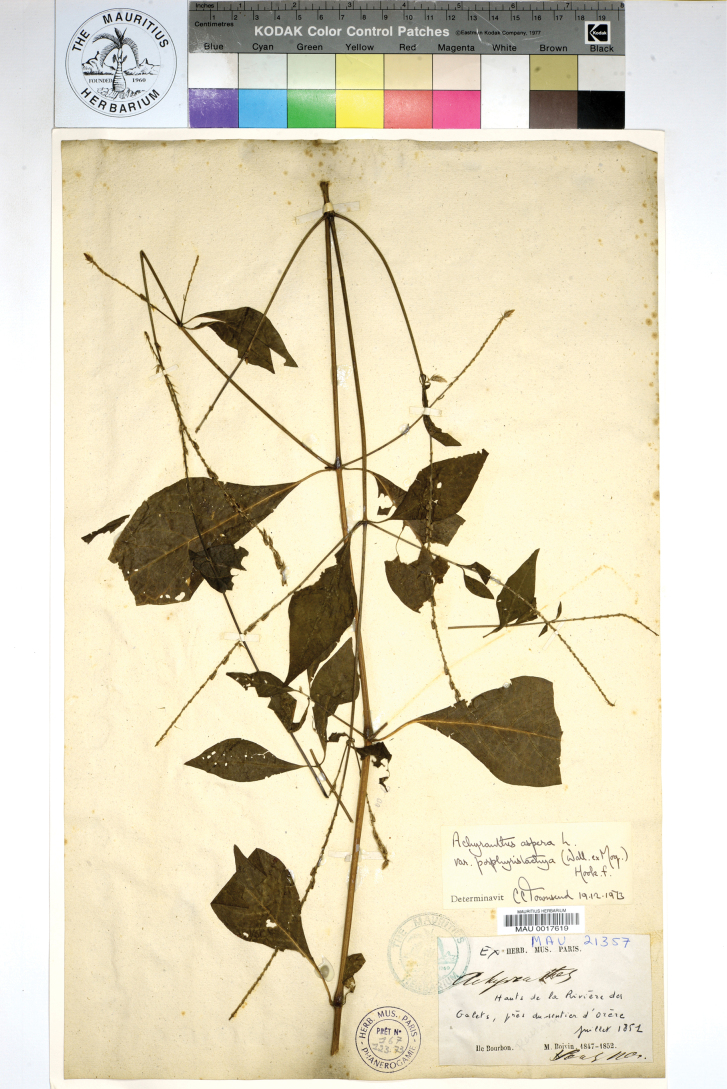
A herbarium specimen of *Achyranthesmauritiana* (Réunion, Rivière des Galets, Jul 1851, *Boivin s.n.*, MAU0017619).

(Fig. 13). Annual (?); stem upright, with spreading lateral branches; leaf petioles 20–40 mm long, blades 30–100 × 20–40 mm, rhombic or elliptic, tip attenuate, almost glabrous or sparsely pubescent at the veins, green, sometimes turning black when dry; inflorescence up to 30 cm long; bract 2.5–3.0 mm long, bracteoles 3.0–3.5 mm; perianth 4.0–5.0 mm long; stamens 5, anthers ~ 0.4 mm long; style 1.0–1.2 mm long; fruit 1.7–2.1 mm long.

##### Taxonomic notes.

The specimens seen are represented by upper twigs only, and our present description is based on the scarce material available in the collections FI-WEBB and MAU. A description provided by Cavaco (1954) is insufficient for any comparison of the material from the Mascarenes, Madagascar and Comoros, because the morphometry of the reproductive characters was not indicated. So far, *A.mauritiana* is still poorly known. Townsend (1994) synonymized *A.mauritiana* with A.asperavar.porphyrostachya [≡ *A.porphyrostachya*], but this synonymy cannot be accepted due to their different leaf shape and morphometry of the reproductive characters. Only a few old collections cited below were from the Mascarenes, with a more recent collection from Agaléga (Mauritian territory). Nevertheless, *A.mauritiana* seems to be a distinct species due to its rhombic or elliptic, almost glabrous leaves as compared with *A.aspera* s.str. Its reproductive characters are the same as in *A.aspera* s.str. The leaf characters are quite similar to those of *A.acuminata*, but the morphometry of the reproductive characters is different. Further studies are needed to explore the morphology, phylogenetic relationships and ecology of *A.mauritiana*.

Townsend (1994) reported A.asperavar.velutina (Hook. & Arn.) C.C.Towns. (≡ *A.velutina* Hook. & Arn.) from Réunion. This robust species with villous leaves occurs only in the Pacific. The plants from the Mascarenes resembling *A.velutina* seem to belong to a different species whose name may be *A.borbonica* Willd. in Roem. & Schult., Syst. Veg., ed. 15 bis 5: 549 (1819) ≡ A.asperavar.borbonica (Willd. ex Schult.) C.C.Towns., Kew Bull. 29(3): 474 (1974). The holotype of *A.borbonica* (“Insula Borbon” [Réunion]) kept at B-W 05-005-010 is represented by a small fragment showing villous leaves and an inflorescence collected in a premature stage. This species is also very poorly known, and no other material except for the type of *A.borbonica* is seen from the Mascarenes.

##### Nomenclatural notes.

Moquin-Tandon (1849) based his *A.mauritiana* on two specimens from Mauritius without exact label information (seemingly collected by P. Commerson during his residence on Mauritius in 1768–1773: Frodin 2001), which were kept in the Paris Museum of Natural History and the personal herbarium of Labillardière. Townsend (1994) designated a specimen at G-DC as lectotype. This specimen belongs to the collection from Mauritius, which was previously kept in Paris but obtained by A.P. de Candolle for his private herbarium by exchange in 1821 (cf. de Candolle 1862). Although this specimen is part (a duplicate) of the collection which was used for the original description, it was not cited in the protologue and therefore has no precedence over cited syntypes in lectotype designation. A specimen at P, which was examined by Moquin-Tandon and is accompanied with his analytical drawing, is designated here as a new lectotype in agreement with Art. 9.12.

##### Distribution.

**Mauritius**: [without exact location and date] *herb*. *Moquin-Tandon s.n*. (FI-WEBB154894); Agaléga, North Island, 19 Jul 1976, *D.R. Stoddart 7237* (US03544411);

**Réunion** (French Overseas Department): Rivière des Galets, Jul 1851, *Boivin s.n.* (MAU0017619).

##### General distribution.

The species seems to be endemic to the Mascarenes (Mauritius and Réunion), but recently collected in Agaléga. Cavaco (1954) cited some records from Madagascar, but all material seen belongs to other species.

#### ﻿ Achyranthes porphyrostachya

Taxon classificationPlantaeCaryophyllalesAmaranthaceae

Wall. ex Moq. in DC., Prodr. 13(2): 316 (1849), as “porphyristachya”.

DE4B1FA5-7159-58A4-AC95-6483FFC31949

≡ Achyranthesasperavar.porphyrostachya (Wall.) Hook.f., Fl. Brit. India 4(12): 730 (1885).
= Achyranthesrobusta C.H.Wright, Fl. Cap. (Harvey) 5(1.2): 428 (1901). Lectotype (designated here): South Africa, [KwaZulu-]Natal, near Durban, 100 ft, [without date] [*Wood*] *7202* (K000243729!).
= Achyranthesasperavar.procera Fiori, Giorn. Bot. Ital., n.s., 19: 437 (1912). Holotype: [Ethiopia, Tigray Region] in vallibus juxta fruticeta 4–8 pedalis prope Adoa [Adwa], 5 November 1838, *Schimper 1234* [Iter Abyssinicum, ser. 2 (1842)] (FI000824!, isotypes BM!, BR0000008356987!, FI-WEBB154842!, K!, L1673915, M0241518!).
= Achyranthesaspera [var.argentea] f.suffruticosa Fiori, Giorn. Bot. Ital., n.s., 19: 438 (1912). Lectotype (designated here): Eritrea, [Dek’emhare] Hamasen, Ghinda – Baresa, 960 m a.s.l., 26 January 1909, A. Fiori 52 (FT001025!; isolectotypes FT001026!, FI!).
= Achyranthesasperaf.robustiformis Suess., Mitt. Bot. Staatssamml. München 1: 70 (1951). Lectotype (designated by Townsend 1985): Tanganyika area [Tanzania], Ngorongoro crater, 5000 ft, 9 April 1941, *P.R.O. Bally 2273* (K!).
– Achyranthesaspera auct. in herb. div.
– Achyranthesasperavar.pubescens auct.: Townsend (1985, 1993b, 1988, 2000), Audru et al. (1994), Boulos (1995), Miller (1996); Wood (1997), Germishuizen and Meyer (2003), Mapaura et al. (2004), Setshogo (2005), Gibreel and Darbyshire (2015), Odorico et al. (2022), Hassan et al. (2023).
– Achyranthesasperaf.fruticosa auct. in herb. div.
– Achyranthesasperavar.late-ovata auct. in herb. K.
– Achyranthesasperavar.pinguispicata Clarke, nom. nud. in herb. K.

##### Lectotype (designated here).

Myanmar. “Tong Dong” [“towards the Hills” = on the way from Sagaing to Shan Hills], 23 November 1826, *N. Wallich in Herb. Wallich 6925/841* (K000848076!; possible isolectotypes G-DC [image!], E00317548!). Other syntype: Myanmar. “Tong Dong” [Thone Taung], 24 November 1826, *N. Wallich in Herb. Wallich 6925/841* (K001126247!).

##### Description.

**Figure 14.**
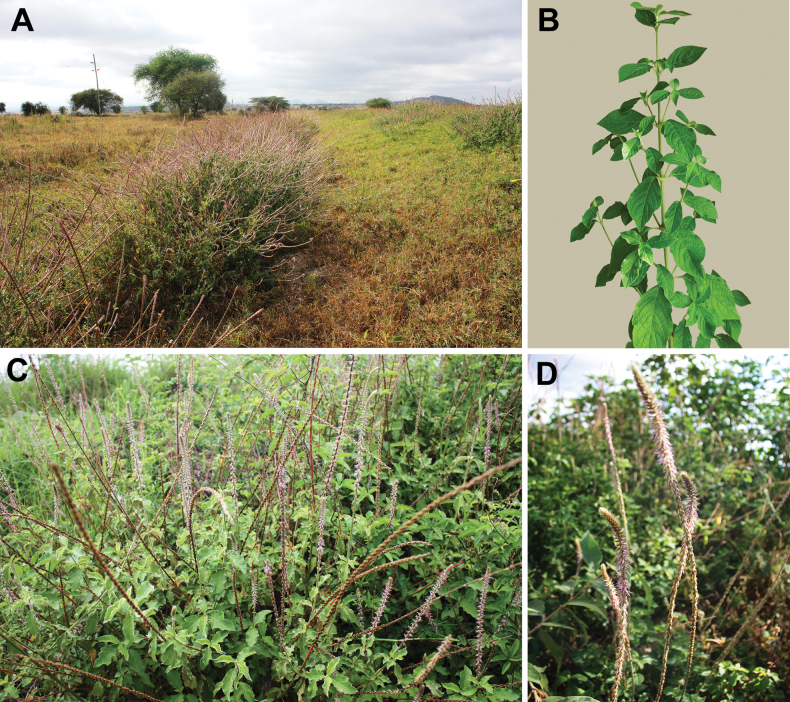
*Achyranthesporphyrostachya***A** population in the open semi-disturbed habitat in Arusha Region, Tanzania **B** an indoor cultivated plant in vegetative stage (grown from seeds from Manyara Region, Tanzania) **C** plant in blooming stage (Kilimanjaro Region, Tanzania) **D** fragment of the inflorescences (Kilimanjaro Region, Tanzania). Photographer: A. Sukhorukov.

(Fig. 14). Subshrub to 2.5(3.0) m tall, stout, not rooting at the nodes; young stem round or indistinctly four-angled; leaf petioles 10–20 mm long, blades 40–140 × 40–80 mm long, ovate (rarely oblong) and entire, dark green above and gray below, both sides hirsute especially below (some individuals probably growing under more humid conditions as well as cultivated indoors may be sparsely pubescent resembling *A.acuminata*); inflorescence long, up to 120 cm, stout, bract 3.5–4.0 mm long; bracteoles 3.0–3.3 mm long; perianth (5.0–5.5)6.0–7.5 mm long, green or pale outside and rose or pink inside, tip often violet; stamens 5 with pink filaments, anthers 0.9–1.1 mm long oblong; pseudostaminodes 1.5–3.5 mm long, white or pink, fimbriate or not; style with stigma (2.5)3.0–4.0 mm long (slightly less than perianth or equalling the perianth); fruit (without style) (2.0)2.3–2.7 mm long.

##### Taxonomic notes.

The variety Achyranthesfruticosavar.pubescens Moq. was described from East Mexico (Moquin-Tandon 1849), and the data about its native status in the Old World, including African references (Wunderlin and Hansen 2011), are erroneous. Two further combinations under *A.aspera* were made: A.asperavar.pubescens (Moq.) M.Gómez, Noc. Bot. Sist.: 58 (1893), and the isonymic A.asperavar.pubescens (Moq.) C.C.Towns., Kew Bull. 29: 473 (1974). The plants growing in Central America have different reproductive characters compared with *A.porphyrostachya* (e.g., Sánchez-Del Pino et al. 2013, as A.asperavar.pubescens). This varietal name cannot be applied to the plants in the Old World, although it was widely used for the perennial African and Arabian populations of *Achyranthes*. A lectotype of A.fruticosavar.pubescens was selected by Townsend (1974) [Tampico de Tamaulipas, 1827, *Berlandier 79-104-105* (G00236794 – image!], and a later lectotypification with the same specimen undertaken by Hassan et al. (2023) is superfluous.

A plant of *A.porphyrostachya* cultivated by APS indoors (seeds originating from Manyara Region, Tanzania) has much more glabrous leaves (Fig. 14B) compared to the voucher specimen located in MW.

##### Nomenclatural notes.

The original spelling “porphyristachya” (Moquin-Tandon 1849) is a correctable error under Art. 60.10. The species name was established on the basis of Wallich’s collections from present-day Myanmar. Moquin-Tandon studied specimens in the personal herbarium of Candolle, which were indicated as “v. s. in h. DC.” in the protologue, and other specimens with the Wallich distribution number, which was cited in the protologue (“Wall.! list n. 6925”). The original material of *Achyranthesporphyrostachya* was collected for and subsequently distributed on behalf of the Honourable East India Company (E.I.C.).

A.P. de Candolle obtained a large set of Wallich specimens (34 packages: Krieger 2023) in 1830, when his son Alphonse visited Wallich in London and negotiated herbarium distribution and taxonomic treatments for himself and his father (Candolle and Radcliffe-Smith 1981). The specimens of *Achyranthes* were dispatched to Candolle prior to their regular distribution from the E.I.C., which was ready in 1832 (Wallich 1832). Labels for the specimens received in 1830 by Candolle had been copied and numbered separately, and were renumbered for the regular distribution later (cf. a note on the double numeration in Compositae: Wallich 1831). On the labels of *A.porphyrostachya*, the Candolle number is 841 (K001126247, G00688936, G00688937), whereas its corresponding Wallich number is 6925 (E00317548, K001126247, K000848076).

The labels of *Wallich 6925* say it is from “Tong Dong 1826”, or Thone Taung Village, Mandalay Region, Myanmar. The Candolle labels are more detailed and informative, and apparently preserve the original field information which was generalised when distribution numbers were formed. Two specimens at G-DC (G00688936, G00688937) have identical labels reading “towards the Hills, 23 Nov. 1826”, indicating the route between the Irawadi River at Sagaing, from which Wallich proceeded towards the Shan Hills on 22 November, being the next day on the way to the Hills “through villages, rice and cotton fields” (Krieger 2023: 178). One specimen at Kew, which also bears a regular distribution label, was originally labelled as “road to Tong Dong” and “Tong Dong, 24 Nov.”, seemingly indicating the arrival at the hilly area of Thone Taung (Candolle and Radcliffe-Smith 1981). This specimen was kept in the Wallich herbarium until its transfer to the Linnean Society in 1857 (Candolle and Radcliffe-Smith 1981), and therefore was not seen by Moquin-Tandon. Two more specimens examined (E00317548, K000848076) bear the standard lithographed distribution labels without further detailed information. The Kew specimen (K000848076) belongs to the set acquired by J.D. Hooker from the possessions of the E.I.C., which was examined in entirety by Moquin-Tandon.

The original material of *A.porphyrostachya* includes two specimens in the personal herbarium of Candolle, which belong to the Wallich collection and bear the species name on original labels with Candolle numbers (examined by Moquin-Tandon according to the protologue), and one specimen in the personal herbarium of Hooker, which was labelled with a lithographed distribution label and annotated by Moquin-Tandon. The latter specimen was directly indicated in the protologue by its distribution number and therefore has precedence in lectotypification. This specimen is formally designated as lectotype here. The two specimens at G-DC and a specimen at E are similar in their appearance and may be treated as isolectotypes.

Achyranthesasperaf.suffruticosa Fiori as lectotypified here is a more glabrous form of *A.porphyrostachya*. Another specimen of the original material (Hamasen: Ghinda, 960 m a.s.l., 26 January 1909, *A. Fiori 954*, FT001027! as A.asperavar.argenteaf.suffruticosa) is referable to *A.annua*.

##### Habitat.

Various disturbed areas, especially near roadsides, in disturbed savanna communities and bush thickets, mostly in the arid and semi-arid regions of Africa, S and SW Asia at elevations of (0)500–2700 m a.s.l. Common in the open ± disturbed highland vegetation types in Tanzania (APS, pers. obs.). A common appearance of *A.aspera* in the mountain steppe-like vegetation in Rwanda, as reported by Mildbraed (1911), probably also belongs to *A.porphyrostachya*. The species is not eaten by livestock according to herbarium collectors.

##### Distribution.

**Figure 15.**
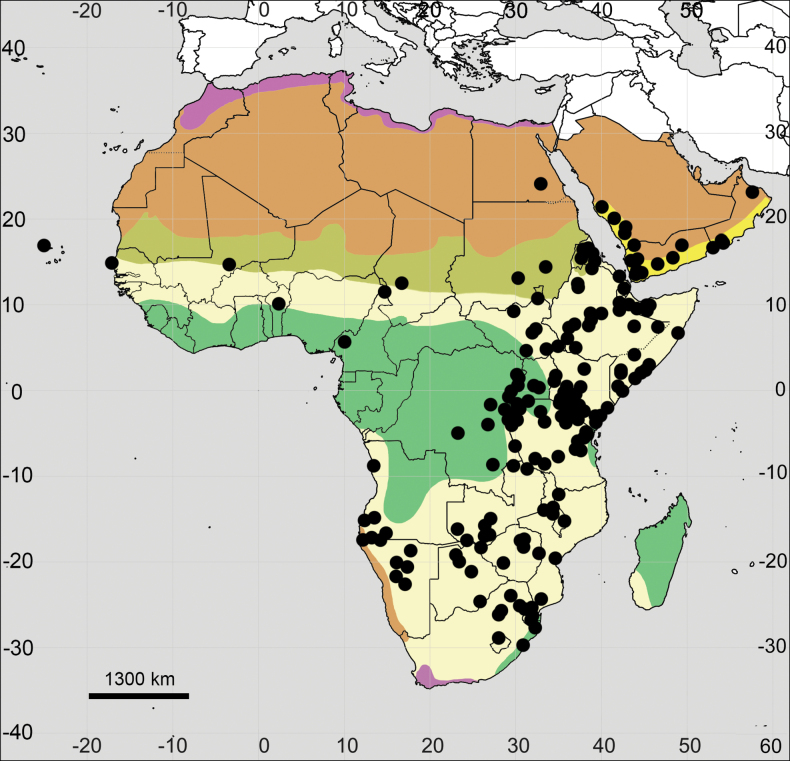
Distribution map of *Achyranthesporphyrostachya*.

(Fig. 15; see also Appendix 1). **Africa**: Angola, Benin, Botswana, Burundi, Cameroon, Cape Verde, Chad, Djibouti, DR Congo, Egypt, Eritrea, Eswatini, Ethiopia, Lesotho, Malawi, Mali, Mozambique, Namibia, Rwanda, Senegal, Somalia, South Africa, South Sudan, Sudan, Tanzania, Uganda, Zambia, Zimbabwe.

**Arabian Peninsula**: Oman, Saudi Arabia, Yemen.

##### General distribution.

Africa, South-West Asia and Indian subcontinent (see also full list of specimens from Asia in Appendix 1).

#### ﻿ Achyranthes seychellensis

Taxon classificationPlantaeCaryophyllalesAmaranthaceae

Sukhor. sp. nov.

8F918270-9A1D-596C-A19D-F5D6BC236333

urn:lsid:ipni.org:names:77353796-1

##### Holotype.

Seychelles, Farquhar Atoll, coconut plantation, 18 August 1984, *G. Ogureeva 608* (MW0586306!, isotype LE00019370!).

##### Description.

**Figure 16.**
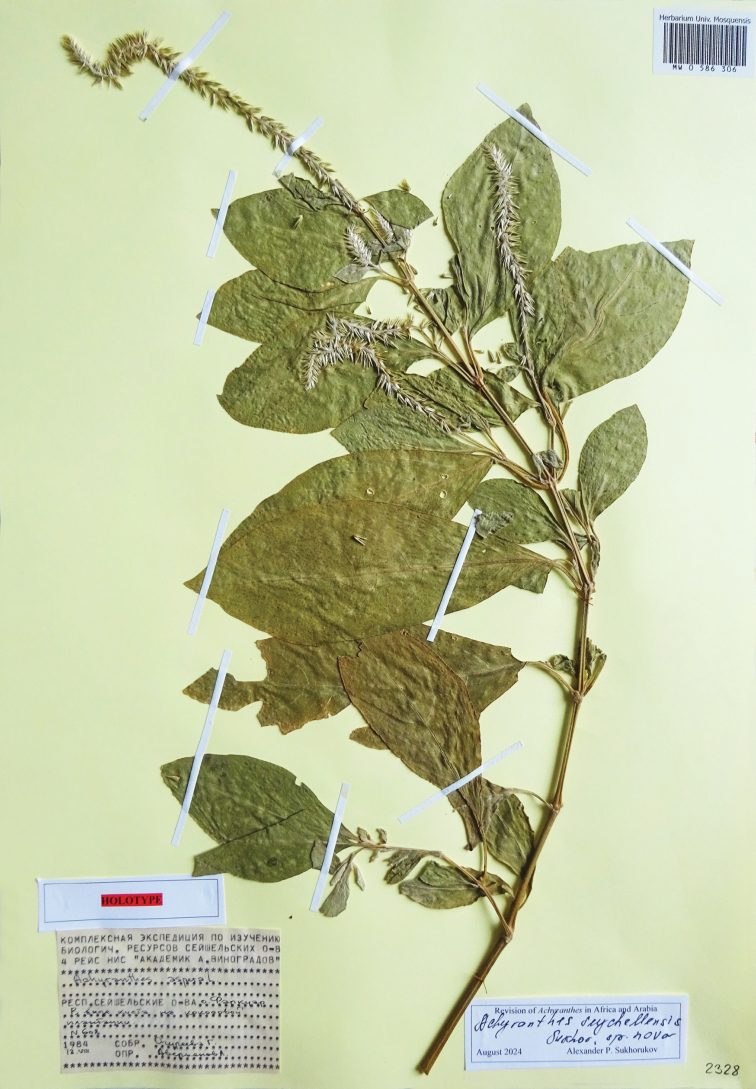
Holotype of *Achyranthesseychellensis* Sukhor., sp. nov. (Seychelles, Farquhar Atoll, coconut plantation, 18 Aug 1984, *G. Ogureeva 608*, MW0586306).

(Fig. 16). Annual herb up to 1.5 m tall; stem stout, basally ± roundish, four-angled in the inflorescence; leaf petioles 10–25 mm long, blades 80–120 × 30–60 mm, cuneate, tip shortly acuminate, green above and pale green below, slightly pubescent on veins; inflorescence up to 25 cm long with hirsute axis; bract 3.5–4.0 mm long; bracteoles 3.2–4.0 mm long; perianth 4.5–5.5 mm long, ± equal, deep rose or purple inside; stamens 5, anthers 0.75–1.0 mm long; pseudostaminodes 1.25–2.0 mm long, brownish or white when dry, fimbriate; style with stigma 2.2–2.7 mm long; fruit (without style) ± 2.0 mm long.

##### Habitat.

Calcareous and rocky areas at elevations of 0–200 m a.s.l. It can be a common weed in coconut plantations.

##### Flowering at fruiting.

Around the year.

##### Additional specimens seen.

**Seychelles**: Sèche Island, 23 Jan 1938, *Ves 6149* (K); St. Pierre Island, Oct 1960, *C.J. Pigeott s.n.* (K); Aride Island, Feb 1983, *F. Friedmann 4469* (P00804851) & Apr 1987, *F. Friedmann 5455* (P00804847); Bird Island, 28 Sep 1952, *E.S. Brown s.n*. (BM); Cousin Island, 25 Jan 1970, *F.R. Fosberg 52173* (K); Fouquet Island, 5 Apr 1976, *D.R. Stoddart 7148* (K); Farquhar Atoll (South), 2 Oct 1967, *Gwynne & Wood 1189* (K); Farquhar Atoll (North), 19 Sep 1968, *D.R. Stoddart 1376* (K); Aride Island, Jul 1975, *Warman 23* (K); Rémire [Eagle] Island, 25 Oct 1976, *S.A. Robertson 2319* (K); Bird Island, Mar 1976, *D.R. Stoddart 7079* (K); Denis Island, 10 Dec 1977, *D.R. Stoddart 8086* (K); Platte Island, 26 Feb 1980, *S.A. Robertson 3021* (K); Poivre Island, 6 Aug 1984, *L. Averyanov & al. s.n*. (LE, MHA); Farquhar Atoll, 16 Aug 1984, *L. Averyanov & al. 580* (LE).

**Mauritius**: Chagos Archipelago, Diego Garcia Atoll, East Island, 26 Jul 1967, *D.R. Stoddart 875* (K); Diego Garcia, near north coast, 30 Mar 1971, *A.M. Hutson 15* (BM); Sea Cow Island, 21 Feb 1975, *M.J.D. Hirons 50* (K).

##### Relationship.

Friedmann (1994) reported three varieties of *A.aspera*: var.aspera, var.velutina, and var.fruticosa from the Seychelles. The specimens seen were identified as var.aspera or var.velutina. From *A.aspera*, the new species differs by longer leaf blades (80–120 × 30–60 mm vs. 15–110 × 20–80 mm), and longer anthers (0.75–1.0 mm vs. 0.4–0.6 mm) and styles (2.2–2.7 mm vs. 1.0–1.1 mm). The true *A.velutina* Hook. & Arn. from the Pacific (Samoa, Tahiti, Tuamotu etc.) is a hirsute shrub with longer (ca. 7 mm long) perianths, but some other characters, i.e. length of anthers and styles are similar. Several specimens from the Aldabra Islands identified as ‘var.fruticosa’ represent plant fragments with a woody base and need further evalution.

##### General distribution.

Endemic to the Seychelles and Chagos Archipelago.

#### ﻿ Achyranthes sicula

Taxon classificationPlantaeCaryophyllalesAmaranthaceae

(L.) All., Auct. Syn. Meth. Stirp. Hort. Regii Taur.: 41 (1773).

78FB6FBF-7B28-5011-8B98-B9DA6A69ADA5

≡ Achyranthesasperavar.sicula L., Sp. Pl. 1: 204 (1753).
≡ Achyranthesargentea Lam., Encycl. 1(2): 545 (1785), nom. illeg. superfl.
≡ Cadelariasicula (L.) Raf., Fl. Tellur. 3: 39 (1837).
≡ Cadelariaargentea Raf., Autik. Bot.: 154 (1840), nom. illeg. superfl.
≡ Achyranthesasperavar.argentea Eggers, Vidensk. Meddel. Naturhist. Foren. Kjøbenhavn 1876: 140 (1877), nom. illeg. superfl.; Boiss., Fl. Orient. 4: 994 (1879), isonym; C.B.Clarke in Th.-Dyer, Fl. Trop. Afr. 6(1): 63 (1909), isonym.
– Achyranthesaspera [var.sicula] f.albida Maire & Weiller, Fl. Afr. Nord. 8: 219 (1962), nom. inval. descr. gall.

##### Lectotype (designated by Townsend 1994).

“Amaranthus radice perpetua” in Herb. Boccone (P).

##### Description.

**Figure 17.**
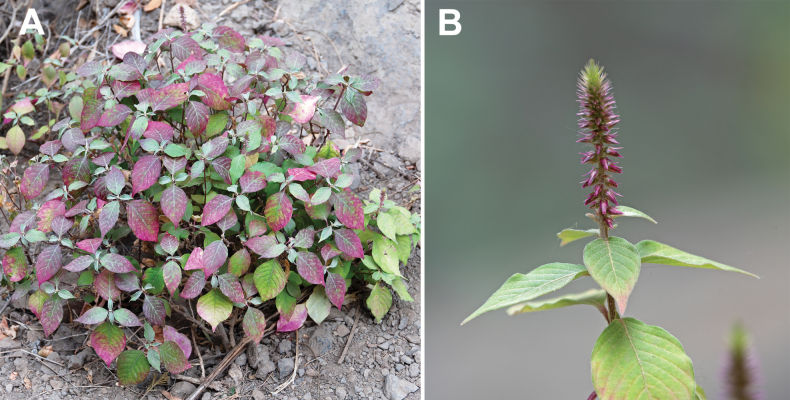
*Achyranthessicula***A** plant in vegetative stage (Santa Cruz de Tenerife, Canary Islands) **B** close-up of the inflorescence (Santa Cruz de Tenerife, Canary Islands). Photographer: F. Bednár.

(Fig. 17). Annual (probably also short-lived perennial), 30–200 cm tall; stem stout, four-angled; leaves shortly petiolate (petioles 5.0–15.0 mm long), blades 25–100 × 15–40 mm, ovate, base broadly cuneate or truncate, tip attenuate, bicolored, green above and white silvery below; inflorescence up to 30 cm long; bract 3.0–4.0 mm long; bracteoles 3.0–4.0 mm long, slightly reflexed, green or usually pink; perianth (3.5)4.0–5.0 mm long, segments ± equal, white, pinkish or green and then turning gray; stamens 5 with white or mauve filaments, anthers 0.5–0.8 mm long; pseudostaminodes 0.5–1.2 mm long, fimbriate, white or pink, style with stigma (1.2)1.5–1.8 mm long; fruit (without style) 1.7–2.0 mm long.

##### Taxonomic note.

Only a limited number of specimens were collected with subterranean parts, and the species seems to be annual despite literature references where it is considered as a perennial or suffruticose herb (e.g., Maire 1962, as *A.aspera*; Zohary 1966, as var.sicula).

##### Nomenclatural notes.

Linnaeus (1753) validly published Achyranthesasperavar.sicula without a description or diagnosis but with a citation of “Amaranthus siculus spicatus. *Bocc. sic. 16. t. 9. Pluk. phyt. 260. f. 2*”. This pre-Linnaean polynomial is not diagnostic because its only descriptive word, “spicatus”, is part of the polynomials similarly cited under another variety of this species, var.indica. For this reason, a validating description of the Linnaean variety is one of the descriptions provided by Boccone (1674) and Plukenet (1691), which were referred to in the protologue. Both descriptions are also accompanied by illustrations, directly cited by Linnaeus (1753), which are part of the original material of the variety. Another part is the specimens associated with the descriptions, namely the herbarium collection of L. Plukenet at BM (Herb. Sloane!) and that of P. Boccone at P. Townsend (1994: 21) accepted Boccone (1674) as a source for the validating description and designated the corresponding specimen “Amaranthus radice perpetua” in Herb. Boccone at P. Although Townsend (1994) made it clear that he designated a herbarium specimen at P, Iamonico (2014) doubted his citation and designated another specimen in Herb. Linnaeus (287.1) at LINN as a new lectotype. The latter specimen is not linked to the validating description of the variety (which is not Linnaean), and therefore Iamonico’s choice is not effective. The lectotype designated by Townsend is technically correct and should be accepted.

When publishing his *A.argentea*, Lamarck (1785) included a full and direct reference to the pre-Linnaean polynomial in its protologue, which was the sole basis of *A.aspera*var.sicula. Under Art. 52.3, Lamarck’s species name is an illegitimate replacement of *A.sicula* (L.) All. The lectotype of *A.argentea* designated by Raus (2022), a specimen from cultivation in the Botanical Garden in Paris and kept at SEV, therefore has no standing.

##### Habitat.

Calcareous soils, rocks, disturbed areas at elevations of 0–1500 m a.s.l.

##### Distribution.

**Figure 18.**
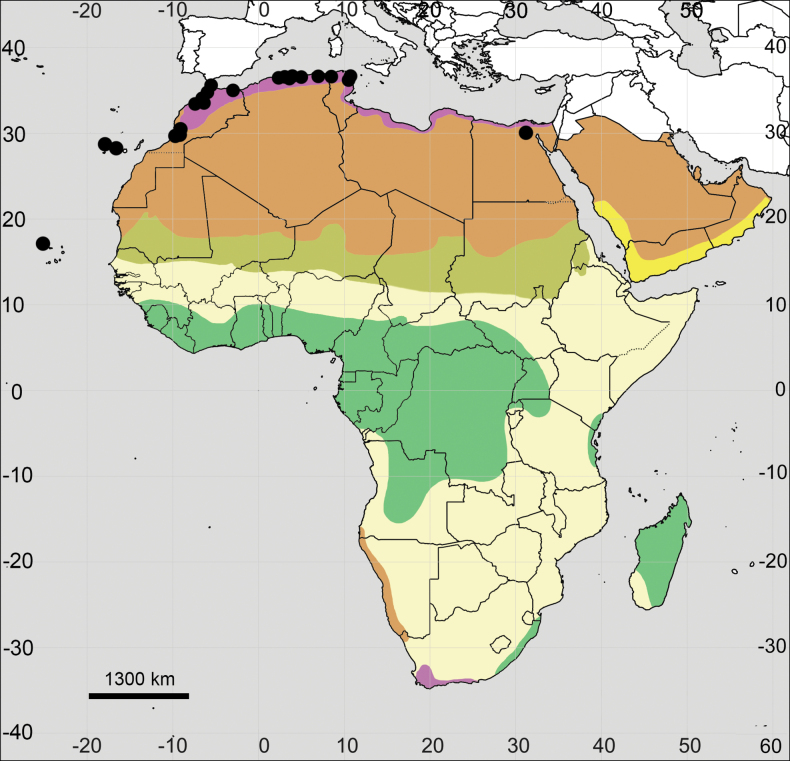
Distribution map of *Achyranthessicula*.

(Fig. 18; see also Appendix 1). **Africa**: Algeria, Cape Verde, Egypt, Morocco, Spain (Canary Islands), Tunisia, and probably Libya (no specimens seen).

##### General distribution.

Mediterranean basin: Algeria, Egypt, Malta, Morocco, Tunisia, British Overseas Territory [Gibraltar], Greece (very rare: Raus 2022), Israel/Palestine, Lebanon, Portugal (Azores and Madeira), Syria, southern part of Italy, Spain. The records of *A.aspera* from Jordan (Taifour and El-Oqlah 2017) probably belong to *A.sicula*.

###### ﻿Notes on other species growing in the regions under study

Two other *Achyranthes* s.str. in Africa have been accepted because of their unique characters: *A.fasciculata* (e.g., Townsend 1985) and *A.talbotii* (Cavaco 1962; Townsend and Darbyshire 2004). The first species is a mountainous scandent shrub with fasciculate inflorescences. *Achyranthestalbotii* is a perennial rheophytic herb frequently rooting at lower nodes, with lanceolate or narrowly oblong leaves. Below we provide currently known localities and distribution maps for both species.

#### ﻿ Achyranthes fasciculata

Taxon classificationPlantaeCaryophyllalesAmaranthaceae

(Suess.) C.C.Towns., Kew Bull. 34(3): 424 (1980).

8B77D03B-00BB-56F5-B085-321BAF8D7103

≡ Pandiakafasciculata Suess., Kew Bull. 4: 477 (1949).

##### Holotype.

Tanganyika Territory [Tanzania, Manyara Region], Mbulu distr., SE slopes of Mt. Hanang, Nangwa, 8000 ft, 6 February 1946, *P.J. Greenway 7620* (K000243725!, isotype BM!).

##### Habitat.

Upland grassland and mist forests at elevatios 1800–2800 m a.s.l.

##### Distribution.

**Figure 19.**
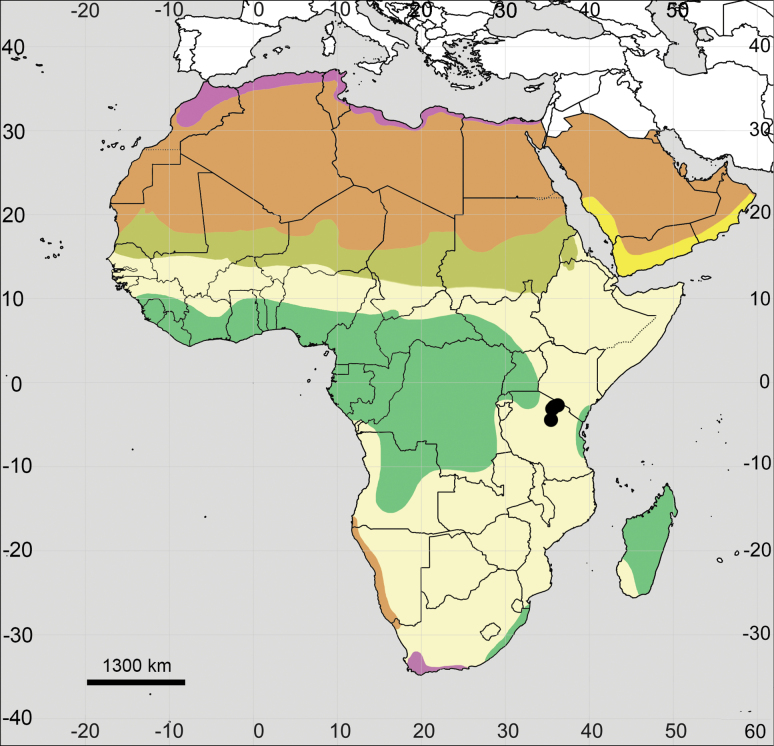
Distribution map of *Achyranthesfasciculata*.

(Fig. 19). **Tanzania** (selected): Ngorongoro crater, 8000 ft, 12 Sep 1932, *B.D. Burtt 4308* (K); Ngorongoro rest camp, 6000 ft, 3 Apr 1941, *P.R.O. Balley 2232* (K); Ngorongoro crater, 7400 ft, 13 Jul 1966, *P.J. Greenway & K. Kanuri 1256662* (BR0000013708887, K); Empakaai crater, 2150 m a.s.l., 10 Aug 1972, *G.W. Frame 27* (BR0000013708870, K); Masai distr., Ngorongoro crater, 2350 m a.s.l., 15 Sep 1977, *J. Raynal 19052* (K, WAG01404450); Arusha Region, Monduli distr., Ketumbeine Forest Reserve, 2330 m a.s.l., 2 Apr 2000, *R.E. Gereau & al. 6428* (CM, MO).

##### General distribution.

Only known from Tanzania. Records from Kenya are also possible.

#### ﻿ Achyranthes talbotii

Taxon classificationPlantaeCaryophyllalesAmaranthaceae

Hutch. & Dalziel, Fl. W. Tr. Afr.: 127 (1927).

362ED53F-AA15-5012-9057-6F82362F9968

##### Holotype.

Nigeria, [probably Osun State] Oran district, 1911–1912, *P.A. Talbot s.n*. (K000243719!).

##### Habitat.

Forests and along streams, often on rocky or volcanic substrates at elevations of 0–1000 m a.s.l. as in *J.P.M. Brenan 9497* (in many herbaria), who adds: “Lava rocks by cataracts above water level, frequent; perennial herb with woody base, ± cespitose stem, purplish-green; leaves papery, dull green ± purple tinged; inflorescence erect, pale green”. *M. Cheek et al. 10401* (K000051090 & WAG1404466) also added: “Lowland evergreen forest around series of waterfalls in river gorge; rheophyte erect herb 15–45 cm tall, rooting in rock crevices and on the surface”.

##### Distribution.

**Figure 20.**
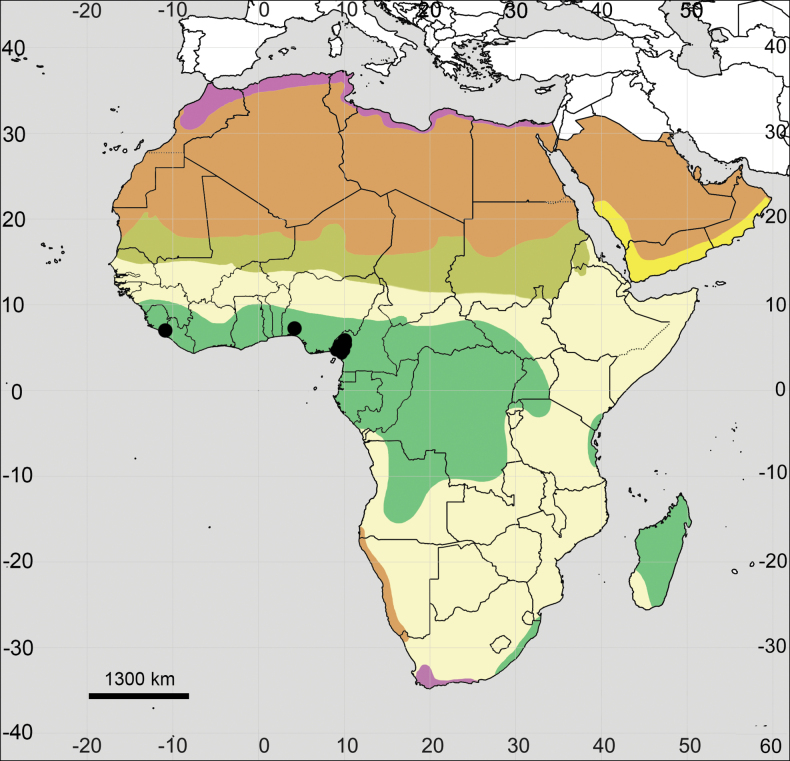
Distribution map of *Achyranthestalbotii*.

Note. Two additional records kept at YA (bank of Nkam River, near Sake, 3 km southwest of Nkondjok, 4°77'N, 10°17'E, 07 Jan 1972, *R. Letouzey 11163*; Kombon at the bank of Kombon River, 4°59'N, 9°26'E, 23 Mar 2011, *F. Kuetegue 316*) and cited by Kuetegue et al. (2019) were also added to the Fig. 20.

(Fig. 20). **Cameroon**: [West Region] Toké, 20 Mar 1948, *J.P.M. Brenan 9497* (BM, BR0000013708900, K000025614, M, P06651712); [Littoral Region] Loum, 20 Dec 1957, *H.C.D. de Wit 392* (WAG0185720); [Littoral Region] 35 km E of Yabassi, 9 Jan 1972, *R. Letouzey 10938* (K, P06651714, YA); Southwest Region, Mt. Cameroon, 350 m a.s.l., 20 Oct 1993, *M. Cheek & al. 5013* (BR0000005004102, K000518867, WAG0255392); Southwest Region, Mokoko, 280 m a.s.l., 26 May 1994, *D.W. Thomas 10167* (K000875887); Southwest Region, Mount Cameroon, Bomana, 400 m a.s.l., 20 Oct 1993, *N. Ndam 742* (K000518868); Kupe-Muanenguba Division, Baseng, 750 m a.s.l., 16 Dec 1999, *M. Cheek & al. 10401* (K000051090, WAG1404466); [Southwest Region] Kumba to Loum, 26 Jan 1986, *H. Breyne 5062* (BR0000013709051, YA); Northwest Region, Bamenda, 620 m a.s.l., 14 Nov 2000, *B.J. Pollard 483* (K001422626); Southwest Region, Mbu River, 27 Nov 2000, *M. Cheek & al. 10635* (K001422627, P00940016); Southwest Region, Nyandong, 400 m a.s.l., 27 Mar 2003, *M. Cheek & al. 11466* (K001518827).

**Liberia**: 30 miles W of Bomi Hills, bank of Lofa River, 12 Nov 1969, *J.W.A. Jansen 1486* (BR0000013833961, K);

**Nigeria**: see holotype.

##### General distribution.

Tropical West Africa. Probably present at least in the countries located between Liberia and Nigeria (Benin, Ghana, Ivory Coast, and Togo) with a similar climate and vegetation. A specimen from Nigeria (Ogoja Prov., Ikom distr., 17 Dec 1950, *R.W.J. Keay 26284*, K000243718!) with wider oblong leaves was noted by Suessenguth as Achyranthestalbotiivar.ogojensis Suess. A similar specimen was also collected in Cameroon (Mount Cameroon, 1830 m a.s.l., 4 Feb 1962, *F.J. Breteler & al. 202*, K000025642!) with a short annotation: “Herb up to 1 m high, stems obscurely quadrangular, green, above the nodes dark red, leaves dark green, paler beneath, frequent”. The taxonomic status of such plants has not been evaluated so far (records of both specimens are not mapped here).

##### Note.

A report of *A.talbotii* from Madagascar and Europa Islands (Cavaco 1962) is unlikely because of different habitats (open grassy vegetation) and a location very remote from the West Tropical Africa. According to Jean Hivert (pers. comm.), the plants from the French Overseas territories belong to a distinct species.

###### ﻿Nomina incertae sedis vel excludenda

Many names in *Achyranthes* have not been resolved yet owing to the absence of their type material in major herbaria. Some authentic specimens of unresolved names may be dispersed through many herbaria, e.g. those of Burman f. (Wijnands 1992), that makes them difficult to trace when the type material was not recognised and remains unavailable electronically. Among species described from Africa, little is known about *A.frumentacea* Burm.f., *A.pedicellata* Lopr., *A.rubrolutea* Lopr., *A.viridis* Lopr., and *A.winteri* Schinz. They are shortly discussed below.

####### *Achyranthesfrumentacea* Burm.f., Fl. Ind. [N.L. Burman] Prodr. Fl. Cap.: 7 (1768).

The protologue in “Prodromus Florae Capensis” is very short and incomplete (Burman 1768), and the most informative phrase is “spicis gracilibus frumentaceis” [spikes subtile, grain-like]. The frumentaceous spikes may refer to the small grain-like diaspores that are present, e.g., in *Achyranthesannua*, a reinstated species also known in South Africa (present paper). Nevertheless, this assumption cannot be confirmed at present, and there are no specimens named as *A.frumentacea* in G-PREL (Nicolas Fumeaux, pers. comm.), the major Burman collection in Europe, as well as in Bibliothèque de l’Institut de France (Sabrina Castandet-Le Bris, pers. comm.). This forgotten name was only mentioned in van Hall (1830) who doubted that the plant described is referable to *Achyranthes*.

####### *Achyranthespedicellata* Lopr., Bot. Jahrb. Syst. 27(1–2): 56 (1899).

Lopriore (1899) described this Central African species in the following way: subshrubby; leaves short (up to 3 cm long); bracteoles hairy; flowers very shortly pedicellate; perianth hairy. The description does not match any *Achyranthes* in a traditional sense. Baker and Clarke (1909) and Cavaco (1962) noted that this name may belong to *Pandiaka*, although Klopper et al. (2006) left it under *Achyranthes* as insufficiently known species.

####### *Achyranthesrubrolutea* Lopr., Bot. Jahrb. Syst. 27(1–2): 57 (1899). [“*rubro-lutea*”]

This species has been transferred to the genus *Pandiaka* (Townsend 1980a, as *P.rubrolutea* (Lopr.) C.C.Towns.) based on the syntypes seen at Z. Therefore, we exclude this species from *Achyranthes*.

####### *Achyranthesviridis* Lopr., Bot. Jahrb. Syst. 27(1–2): 56 (1899).

This species described by Lopriore (1899) from Tanzania is characterized by short (up to 2.5 cm) internodes and capitate inflorescences up to 1 cm long consisting of densely arranged flowers with glabrous perianth segments. Cavaco (1962) thought that *A.viridis* more likely belongs to A.asperavar.sicula, but the short internodes and capitate inflorescences are not characteristic of any *Achyranthes* and may refer to *Alternanthera*, although Klopper et al. (2006) left it under *Achyranthes* as insufficiently known species.

*Achyrantheswinteri* Schinz ex Peter, Repert. Spec. Nov. Regni Veg., Beiheft 40(2,3): 240 (1938), nom. inval. Schinz (in Peter 1938) briefly described this new species (in German) as having ovate leaves and solitary flowers with fimbriae. Such characters are not informative, and the species is absent in a diagnostic key provided by Peter (1938). The key includes *Achyranthes* in a traditional sense as well as the species with a pubescent perianth (not belonging to *Achyranthes* s.str.) and *Centrostachysaquatica*. Klopper et al. (2006) consider it as an insufficiently known species, probably based on Townsend (1985) who noted that the original specimens cited in the protologue (Peter 1938) were probably destroyed in Berlin during the WW2.

## ﻿Discussion

### ﻿Species richness and geographical pattern of *Achyranthes* (excluding *Achyropsis*) in Africa and Arabia

The specific diversity of *Achyranthes* in Africa is not as small as previously thought. Instead of 3–4 species reported, we accept 10 species that are morphologically well-distinguished (*Achyropsis* is not included in this number, but if this genus is synonymized with *Achyranthes*, the number of species will increase to 16). Almost all species have large ranges covering many regions of the continent except *A.fasciculata* that seems to be restricted to Tanzania (with possible records in Kenya) and *A.talbotii* Hutch. & Dalziel that possesses a relatively small range in West Tropical Africa (Cameroon, Liberia, and Nigeria). *Achyranthesmauritiana* and *A.seychellensis* are restricted to the islands of the Western Indian Ocean.

The optimal ecological conditions in some parts of Africa and Arabia enabled the evolution of *Achyranthes*, and its spiny bracteoles attaching the seed-containing diaspore to animals or humans promoted ± long-distance epizoochorous dispersal (Bullock and Primack 1977). Additionally, the diaspores can also be dispersed by water (nautochory: Müller-Schneider 1983), e.g. in riverine areas for *A.acuminata*. Each species in the former *A.aspera* aggregate, despite extensive distribution, has different geographical patterns and ecology.

#### ﻿Mediterranean zone (mauve color in the distribution maps)

It stretches along the Mediterranean coast in North Africa (Algeria, Egypt, Libya, Morocco, and Tunisia) as well as a small strip near the Cape Peninsula in South Africa. Only *Achyranthessicula* is found in this small zone in North Africa, where it grows on rocky substrates, in woodlands and sclerophyllous environments, or as a weedy plant with summer dormancy. Based on the general distribution of this species along the Mediterranean Sea (Tutin 1964; Zohary 1966; Jalas and Suominen 1980) with radiations into Macaronesia, it is supposed that *A.sicula* is a true subtropical species native to the Mediterranean basin. *Achyranthesannua* reaches the Mediterranean zone, but it seems to be rare in North Africa based on its scattered records and is more frequently found in the Cape Peninsula. No specimens of *A.aspera* s.str. were found from North Africa in the herbaria visited despite some recent reports (Greuter et al. 1984; Iamonico 2015; Iamonico and El Mokni in Raab-Straube and Raus 2018), with the exception of an old and unconfirmed record from Egypt, which is more likely to have been collected in the southern part of the country.

#### ﻿Deserts (brown color in the distribution maps)

The Desert zone lies between the Mediterranean and Sahel zones in North Africa and covers almost all parts of the Arabian Peninsula, except small coastal strips. The largest Sahara Desert (including North Saharan woodlands) as well as Namib Desert in south western Africa belong here too. Scattered records of drought-adapted species (e.g., *A.aspera* s.str., *A.porphyrostachya*) are known from this zone, and they are confined to irrigated places or the Nile River valley, e.g. a single old record of *A.porphyrostachya* from Aswan town (K! also cited by Boulos (1995) as A.asperavarpubescens). In general, no *Achyranthes* species are common in the desert zone.

#### ﻿The Sahel zone (olive-green color in the distribution maps)

This zone with the hot semi-arid climate and mainly flat topography is suitable for drought-adapted *Achyranthes* species such as *A.annua* and *A.porphyrostachya*. Being a shade-loving annual species, *A.annua* is usually found in *Vachellia* bushland or as a weed under the canopy during the wet season; otherwise, it is collected at higher elevation (e.g. the Marrah Mountains, Sudan). Records of the subshrubby *A.porphyrostachya* are more frequent in the eastern part of the Sahel zone in open and semi-shaded disturbed plant communities. *Achyranthesabyssinica* is confined to the eastern part of the Sahel, growing in the mountains at elevations of 1400–2500 m a.s.l., where it has its northern distribution limit.

#### ﻿Grassland and savanna (pale color in the distribution maps)

Various types of grassland and savanna are widespread in eastern and southern Africa with low rainfall and longer dry seasons (Huntley 1982). This zone incorporates all species of the former *A.aspera* aggregate, and they are mostly growing at elevations between 1000–2200 m a.s.l. under different ecological conditions (*A.abyssinica* in various transitional landscapes; *A.acuminata* in shady wet places, mainly near streams; *A.annua* in *Vachellia*–*Commiphora* shrublands; *A.porphyrostachya* in various secondary areas). *Achyranthesaspera* s.str. is a common weed below 500 m a.s.l. (e.g., in the coastal regions of Kenya and Tanzania).

The Sahel, grasslands and savannas are more or less arid zones of Africa where the vegetation is influenced by rainfall. For example, field observations near Balangida Lake (~ 1940 m a.s.l., Manyara Region, Tanzania) show that seed germination of *A.annua* starts in the beginning of the rain season (March), with the plants completing their life cycle by mid-June, before the dry season.

#### ﻿Rainforest zone (jade green color in the distribution maps)

It includes areas of the Atlantic Equatorial coastal forests and Congolian lowland forests with no or very short dry season. Remarkably, only *A.acuminata* is widespread in this zone; all other, mostly drought-adapted species (*A.abyssinica*, *A.porphyrostachya*) are absent or grow at margins of rainforests and savannas (e.g., *A.abyssinica* and *A.porphyrostachya* reaching the western branches of the East African Rift). There are a few records of *A.aspera* s.str. in this area, although we expect it should more frequently occur as a weed.

#### ﻿Arabian Peninsula (brown color for deserts and yellow color for shrublands)

Remarkably, all *Achyranthes* (*A.abyssinica*, *A.annua*, *A.aspera*, *A.porphyrostachya*) were collected near the coastlines and in the mountains in the southernmost part of the peninsula which is characterized by milder climatic conditions connected with seasonal rainfalls and diverse topography (Fleitmann and Matter 2009). The inland territories include extremely dry sandy plains with very low precipitation (Almazroui et al. 2012). Among these species, *A.aspera* seems to always grow in ruderal habitats.

### ﻿Further perspectives for revealing the diversity of the genus in the World

We have shown that Africa has many more species of *Achyranthes* than was previously believed. In the rest of the World, the exact species number is yet to be evaluated. The Pacific contains several shrubby endangered endemics as those from the Hawaiian Archipelago (John 1979), Norfolk Islands (two species: De Lange and Murray 2001) and the extinct *A.mangarevica* Suess. from the Mangareva Island (Gambier Islands, French Polynesia: Florence 2004). Australia, Asia and the Americas lack a recent revision of the genus.

Besides the *A.aspera* aggregate revised here, the *A.bidentata* group that is distributed across the Asian tropics and subtropics should be reviewed. This species group is represented by rhizomatous herbs.

## ﻿Conclusion

This first in-depth morphological study of *Achyranthes* detected a remarkable taxonomic diversity of the genus in Africa and Arabia, with new nomenclatural changes, and elaborated ecological and chorological information. The systematics of *Achyranthes* has not been fully understood so far, and both morphological and phylogenetic studies are needed to advance knowledge about the diversification and evolution of this genus worldwide. The African continent represents a major centre of the specific diversity of *Achyranthes*.

## Supplementary Material

XML Treatment for Achyranthes abyssinica

XML Treatment for Achyranthes acuminata

XML Treatment for Achyranthes annua

XML Treatment for Achyranthes aspera

XML Treatment for Achyranthes mauritiana

XML Treatment for Achyranthes porphyrostachya

XML Treatment for Achyranthes seychellensis

XML Treatment for Achyranthes sicula

XML Treatment for Achyranthes fasciculata

XML Treatment for Achyranthes talbotii

## ﻿Additional information

### Author ORCIDs

Alexander P. Sukhorukov https://orcid.org/0000-0003-2220-826X

Maria Kushunina https://orcid.org/0000-0003-2541-6494

Maya V. Nilova https://orcid.org/0000-0001-5340-5915

Cláudia Baider https://orcid.org/0000-0002-2203-2076

Alexander N. Sennikov https://orcid.org/0000-0001-6664-7657

### Data availability

All of the data that support the findings of this study are available in the main text.

## ﻿Appendix 1

**Records of *Achyranthesabyssinica* , *A.acuminata* , *A.annua* , *A.aspera* s.str., *A.porphyrostachya* , and *A.sicula* from Africa and Arabia based on the examined herbarium specimens and identifiable online images. Records of *A.mauritiana* and *A.seychellensis* are given in the main text due to the limited number of collections.**

***Achyranthesabyssinica* Nees**

**Africa**

**Angola**: [Lunda Norte Prov.] Bumba, Mar 1879, *F. Welwitsch 6552* (K);

**Burundi** (new records; selected): 7 km of Muramvya, 2200 m a.s.l., 31 Mar 1956, *J. Symoens 2362* (BR0000005352647); [Western Prov.] Bugarama, 2200 m a.s.l., 7 Jan 1966, *J. Lewalle 216* (BR0000013837167); [Bujumbura Rural Prov.] Ijenda, 16 Mar 1966, *J. Lewalle 526* (BR0000013837174); Muramwya Prov., Teza, 2200 m a.s.l., 9 Apr 1979, *M. Reekmans 7782* (BR0000013708597); Muramwya Prov., Teza, 2000 m a.s..l, 15 Feb 1981, *M. Reekmans 9605* (K, WAG1404354); [Bubanza Prov.] Bubanza town, 2 May 1981, 2000 m a.s.l., *M. Reekmans 10047* (K, WAG1404353);

**Cameroon** (new records; selected): [Northwest Region] Bamenda, 8000 ft, 18 Jan 1928, *F.W.H. Migeod 18128* (BM, K, M); [Northwest Region] Ndu, 6400 ft, 5 Jan 1951, *R.W.J. Keay & T.A. Russell 28439* (K); [Southwest Region] Buea, Dec 1952, *G.K. Akpabla s.n*. (K); [West Region] Djuttitsa, 1800 m a.s.l., 16 Jul 1955, *A. Saxer 192* (K); [Adamawa Region] 10 km S of Ngaoundéré, Oct 1960, *F.J. Breteler 804* (BR0000013834197, K); [West Region] Dschang city, 1300 m a.s.l., 6 Dec 1965, *A. Meurillon 203* (BR0000013709020, K); Adamawa Region, Hosséré Sillé, 20 Oct 1967, *H. Jacques-Félix 8742* (K); Mt Hosséré Vokré, 30 Oct 1967, *H. Jacques-Félix 8480* (BR0000013834135); Mt. Kupe, 20 Jan 1972, *A.J.M. Leeuwenberg 9254* (K); Northwest Region, Nkambé, 4 Nov 1974, 2130 m a.s.l., *R. Letouzey 13143* (K); Northwest Region, Nso, 22 Sep 2005, *L.J.G. van der Maesen 59* (K); Southwest Region, Nyassosso, 19 Jan 1996, *M. Etuge 1597* (K);

**DR Congo**: see (iso)lectotype of Achyranthesasperaf.rubella; [North Kivu Prov.] Rutshuru, 24 Dec 1936, *J. Ghesquière 3527* (BR0000016287532, K); [Lualaba Prov.] Lubudi, 1937, *D. Cabu 133* (BR000001378832); [Haut-Katanga Prov.] Lukafu, Jun 1939, *P. Quarré 5472* (BR0000013836931); [South Kivu Prov.] Mulungu, 20 Feb 1940, *F.L. Hendrickx 1391* (K); [Tanganyika Prov.] Marungu Highlands, 2300 m a.s.l., Apr 1944, *L. Dubois 1207* (BM, BR0000013707958, K); Upemba NP, Apr 1948, *G.F. de Witte 3706* (K); [South Kivu Prov.] Tshibinda, 27 Feb 1951, *R. Pierlot 55* (BR0000013836597); [South Kivu Prov.] Kalonge, 2000 m a.s.l., 14 Jul 1954, *H.A. Osmaston 3307* (BM); [North Kivu Prov.] Sake town, 6 Aug 1957, *R. Devred 3878* (BR0000013836177, K, W0333212); [North Kivu Prov.] Rumangabo, 16 Feb 1958, *A. Leonard 632* (BR0000013836573); [South Kivu Prov.] Birava, 5 May 1958, A. *Meurillon 551* (BR0000013835774); [South Kivu Prov.] Kilimbwe, 16 Nov 1977, *T. Yamada 177* (K); North Kivu Prov., Rutshuru, Virunga NP, Jun 2009, *J. Mangambu Mokoso 2054* (BR0000016287709);

**Eritrea** (new records; selected): Bel’ta [Mt.] 1800 m a.s.l., 4 Feb 1893, A. *Terracciano & A. Pappi 952* (FT); [Anseba Region] Gekeb [Subregion], 7 Feb 1893, A. *Terracciano & A. Pappi 1900* (FT); [Debub Region] Urug valley, 22 Mar 1893, *A. Pappi 3337* (FT); [Maekel Region] Az-Nefas, 2 Feb 1902, *A. Pappi 3593* (BR0000016286566, FT, W0333250); [Maekel Region] Ad Rassi, 28 Apr 1902, *A. Pappi 4969* (FT); [Debub Region] Sala Daro, 7 Oct 1902, *A. Pappi 2322* (FT); [Northern Red Sea Region] Comaile valley, 4 Mar 1903, *A. Pappi 5923* (FT); Adi Ugri [Mendefera town], 12 Oct 1903, *A. Pappi 64* (FT); nr Asmara, 7600 ft, 22 Aug 1954, *J.W. Colville 6* (FT, K); nr Asmara, 20 Sep 1956, *C.F. Hemming 1050* (BM, K);

**Ethiopia** (selected): see neotype; Amhara Region, Dembiya, 26 Sep 1909, *G. Caramelli 2221* (FT); Addis Ababa, Dec 1911, *E.A. Mearns 22* (BR0000016286559); [Amhara Region] Gondar, 3 Jun 1915, *R.E. Massey 42* (K); [nr Tana Lake] Zege Peninsula, 14 Feb 1937, *R. Pichi-Sermolli 1442* (FT); Qounzela [Consuela] town, 23 Feb 1937, *R. Pichi-Sermolli 1444* (FT); [Amhara Region] nr Debre Tabor vill., 12 Mar 1937, *R. Pichi-Sermolli 1439* (FT); [Afar Region] Afrera [Lake], 13 Nov 1937, *A. Vatova 571* (FT); [Amhara Region] Dessie, 10 Aug 1946, *G.P. Hall s.n*. (K); nr Tana Lake, 9 Sep 1953, *anonymus 39* (K); Addis Ababa, 8000 ft, 26 Nov 1954, *H.F. Mooney 6400* (FT, K); French Somalia [Somali Region], Gode, 800 m a.s.l., Mar 1956, *E. Chedeville 1548* (FT); [Somali Region] Mulu town, Sep 1956, *H.R.D. Sanford 65* (FT); [Oromia Region] Dembidolo, 6300 ft, 2 Mar 1957, *H.F. Mooney 6860* (K); [Amhara Region] Wof Washa Forest, 9000 ft, 24 Mar 1957, *H.F. Mooney 6995* (K); [Amhara Region] Gojjam, Choqa Mt., 10000 ft, 6 Aug 1957, *I.M. Evans 472* (K); 35 km W of Addis Ababa, 2400 m a.s.l., 23 Sep 1957, *H. Smeda 566* (FT); [Oromia Region] Alamaya, 4 Apr 1958, *Agriculture College Team B-3* (K); [Oromia Region] between Metu & Gore, 5000 ft, 6 Apr 1958, *F. Piffard 114* (K); [nr Addis Ababa city] Entoto [Mt.], 2500 m a.s.l., 1960, *K. Hildebrandt 45* (WU); [Amhara Region] nr Kobo, 27 Aug 1961, *W. Burger 708* (K); nr Addis Ababa, 2600 m a.s.l., 11 Oct 1963, *W.J.J.O. de Wilde & B.E.E. de Wilde-Duyfjes 8212* (K); 25 km E of Lekemti, 2000 m a.s.l., 1 Jul 1965, *W.J.J.O de Wilde & B.E.E. de Wilde-Duyfjes 7179* (BR0000013834418); 8 km N of Addis Ababa, 11 Oct 1965, *W.J.J.O de Wilde & B.E.E. de Wilde-Duyfjes 8217* (BR0000013834364); [Oromia Region] nr Kulubi, 2400 m a.s.l., 5 Dec 1968, *J.J.F.E. de Wilde 4140* (BR0000013834449); [Sidama Region] Dara, 9000 ft, 1968, *P.M. Mulwany 87* (K); [Oromia Region] 8 km S of Jimma, 8 Nov 1970, *I. Friis & al. 179* (K); [Oromia Region] Mai Gudo Mt., 2400 m a.s.l., 3 Dec 1972, *K. Vollesen 1547* (K); 7 km on road Worata–Debre Tabor, 31 Dec 1972, *C.J.P. Seegeler 3043* (BR0000016286535, WAG1404368); Haberge, Alemaya, nr Bari vill., 2000 m a.s.l., 31 Aug 1976, *P.C.N. Jansen 7051* (BR0000013834357); [Oromia Region] E of Negele town, 2000 m a.s.l., Nov 1976, *D.R. Chaffey 1102* (K); [Amhara Region] Mekdela, 10500 ft, 11 Oct 1995, *V. Purchon & G. Belachew 37* (K);

**Guinea** (new record): Simandou Range, May 2009. *P.K. Haba 590* (K);

**Kenya** (selected): see type of Achyranthesasperaf.latifolia; Taita Hills, 10000 ft, 1898, J.*W. Gregory s.n.* (BM); [Nyeri County], Nyeri, 18 Dec 1921, *R.E. Fries & Th.C.E. Fries 84* (K); Chyulu Hills, 5200 ft, 24 Apr 1938, *P.R.O. Bally 7970* (K); [Nakuru County] Molo, 28 Jan 1947, *A. Bogdan 171* (K); [Rift Valley Region] Mau Forest, 24 Jan 1948, *P.R.P. Bally 89959* (K); Nakuru County, Eastern Mau Forest Reserve, 2300 m a.s.l., 4 Sep 1949, *R.A. Maas Geesteranus 6113* (BR0000013834616, L1673908); Mt. Elgon, Nov 1949, *Tweedie 802* (M); Furroli Mt., 20 Sep 1952, *J.B. Gillett 13954* (M); [Rift Valley Prov.] Mau Forest, Oct 1961, *O. Kerfoot 2921* (FT); nr Nairobi, 7000 ft, 15 Jun 1963, *J. Stewart 720* (K); Rift Valley Prov., Nakuru County, 15 Nov 1967, *B.E. Perdue & S.P. Kibuwa 9061* (BR0000013834555); Narok distr., 8 Jul 1972, *P.J. Greenway & K. Kanuri 15021* (K, M); Nyanza Prov., Kisii distr., between Ikoba & Ogembo, 1750 m a.s.l., 29 Oct 1974, *D. Vuyk & F.J. Breteler 41* (W0333203); [Narok County] 9 km NE of Entasekera, 6700 ft, 28 Sep 1977, *C. Fayad 201* (K); [Samburu County] between Barsaloi & Maralal, 2000 m a.s.l., 22 Nov 1977, *S. Carter & B. Stannard 613* (K);

**Madagascar** (new record): Antananarivo Prov., 15 km NW of Antananarivo city, 11 Sep 1980, *M.A. Fischer & al. 85* (WU);

**Malawi** (new records; selected): [Southern Region] Cholo Mt., 1200 m a.s..l., 21 Sep 1946, *L.J. Brass 17708* (K); [Northern Region] Mughese Forest Reserve, 6000 ft, Sep 1953, *E.G. Chapman 1637* (BM); Chisongole forest, 4500 ft, 25 Aug 1956, *E.I. Newman & T.C. Whitmore 576* (BR0000013709082); [Central Region] Dedza Mts., 16 Apr 1961, *J. Chapman 1236* (K); Central Region, Dedza Mts., 1780 m a.s.l., 1 Apr 1970, *R.K. Brummitt 9571* (K); Northern Prov., Businande, 6500 ft, 28 Dec 1975, *E. Phillips 757* (K);

**Nigeria** (new records): [Taraba State] Kamatan Forest Reserve, 5 Dec 1968, *B.O. Daramola 62482* (K); [Taraba State] Chappal Waddi, 7000–7500 ft, 19 Nov 1969, *J.K. Jackson & al. 1985* (K);

**Rwanda** (new records): [Western Prov.] Shangugu, 1929, *J. Claessens s.n*. (BR0000016287402); Shangugu, Astrida to Bukavu, 12 Feb 1959, *G. Troupin* 11295 (BR0000013708429); Kibungo [Eastern] Prov., Akagera NP, 17 Apr 1969, *G. Bouxin & M. Radoux 224* (BR0000013708214, K); [Western Prov.] Cyangugu, 23 Jul 1974, *P. Aquier 3362* (BR0000013708160); Visoke [Bisoke] Mount, 2700 m a.s.l., 9 Feb 1975, *W.G. D’Arcy 7687* (K);

**Somalia** (new records): Somaliland, Golis Range, Jun 1895, *E.L. Phillips s.n.* (K); [Sahil Region] Berbera, 1905, *G.W. Bury s.n*. (BM);

**South Sudan** (new records): Katire to Itibol, 6350 ft, 17 Dec 1935, *anonymous 1637* (BM, K); Imatong Mts., 8000 ft, 10 Feb 1936, *H.N. Johnston 1484* (BR0000016286511, K);

**Sudan** (new records): Darfur Region, Jebel Marrah, Mar 1921, *H. Lynes 62* (BM); Darfur Region, between Jebel Uo & Nyuringya, 2200–2300 m a.s.l., 4 Jan 1934, *J.E. Dandy 128* (BM, BR0000013834272); Jebel Marrah, 8 Sep 1964, *G.E. Wickens 2425* (K); Central Darfur, 120 km E of Zalingei, Jebel Marrah Mts., 1650 m a.s.l., 20 Jan 1965, *W.J.J.O de Wilde & al. 5437* (BR0000013834296);

**Tanzania** (new records): Mt. Kilimanjaro, Aug 1893, *G. Volkens 678* (K); [Njombe Region] Lupembe, Mar–Apr 1931, *H.J. Schlieben 633 & 369* (BM, BR0000016286733); Uluguru Mts., 900 m a.s.l., 28 Mar 1933, *H.J. Schlieben 3688* (BM, M); Kilimanjaro, 2200 m a.s.l., 14 Feb 1934, *H.J. Schlieben 4766* (BR0000016286665, M); [Morogoro Region] Morogoro town, 4500 ft, Dec 1934, *E. Bruce 219* (BR0000016286634); [Mbeya Region] Mbeya, 1 May 1935, *H.E. Emson 392* (K); [Kigoma Region] Pasagulu Mt., 4800 ft, 8 Aug 1959, *R.M. Harley 9212* (BR0000013835026); [Kagera Region] Ngara, 5000 ft, 29 Dec 1960, *R. Tanner 5558* (K); Ngorongoro crater, 2450 m a.s.l., Mar 1968, *R. Ravazzano s.n*. (FT); Ngurdoto crater, 1900–2000 m a.s.l., 21 Mar 1968, *R. Ravazzano 28* (FT); [Tanga Region] Lushoto distr., 10 Mar 1969, *S. Shabani 332* (BR0000016286719, K); [Arusha Region] Karatu town, 1750 m a.s.l., 15 Sep 1977, *J. Raynal 19077* (BR0000016286696); Iringa Region, Mufindi, 18 Mar 1986, *S. Bidgood & P. Keeley 302* (K); [Kilimanjaro Region] Chambogo, 6 Jul 1987, *F.R. Kisena 532* (K); Arusha Region. nr Meru NP, -3.314720, 36.880373, ca. 2000 m a.s.l., 16 Jun 2021, *A.P. Sukhorukov 7* (MW, W); Arusha Region, nr Arusha town, Moshono, -3.390028, 36.716360, 16 Jun 2021, A.P. Sukhorukov 5 (MW); Arusha Region, nr Arusha town, Sasi, -3.379505, 36.751847, 1 Dec 2021, *A.P. Sukhorukov s.n.* (MW);

**Uganda** (new records; selected): [Northern Region] Mt. Rom, 7300 ft, 1936, *W.J. Eggeling 2385* (BR0000016286573); Western Region, Kigezi distr., 22 Apr 1950, *H.C. Dawkins 573* (BR0000016286597, K, M); Kigezi distr. [now Western Region], 12 Jan 1933, *H.M. Gardner 356* (BR0000013834487); Western Prov., 14 Nov 1954, *H. Stauffer s.n.* (BR0000013834470, K); Elgon Mt., 2750 m a.s.l., 6 Jan 1996, *K. Wesche 681* (K); Western Region, Kisoro distr., nr Mgalimnga NP, 1700 m a.s.l., 20 Jun 2005, *B. Rose s.n.* (W0333285).

**Arabia**

**Oman** (new record): Dhofar Governorate, Ayn Razat Spring, 24 Sep 1991, *H.D.V. Prendergast s.n.* (K);

**Saudi Arabia** (new records): Asir Prov., nr Al Habala, 8 Mar 1981, *D. Hillcoat 128* (BM); Al-Baha Region, 230 km S of Taif, 2000 m a.s.l., 14 Apr 1988, *A.A. Fayed 1420* (M);

**Yemen** (new records): [Dhale Governorate] Ash Shu’ayb distr., 2 Jun 1931, *C. Rathjens s.n.* (BM); Jabal Jihaf Mt., 7500 ft, 4 Oct 1937, *H. Scott & E.B. Britton 126* (BM); Yarim, 9300 ft, 21 Sep 1972, *J.R. Wood 55* (BM); [Dhamar Governorate] Dhamar town, 2420 m a.s.l., 30 Sep 1981, *D. Podlech 36061* (M, MSB122471); nr Sana’a, 2450 m as.l., 30 Sep 1981, *D. Podlech 36039* (M); between Sana’a & Manakha, Mar 1988, *Y. Ghyoot s.n*. (BR0000023108011).

***Achyranthesacuminata* E.Mey. ex Sonder**

**Africa**

**Angola** (new records): [Cuanza Norte Prov.] Golungo Alto, May 1856, *F. Welwitsch* 6544 (BM, K); [Cuanza Norte Prov.] Cazango, Jul 1911, *J. Gossweiler 5148* (BM); nr Luanda, 750 m a.s.l., Nov 1946, *J. Gossweiler 13670* (K); Cuando Cubango Prov., Cuito River, 1059 m a.s.l., 11 Jun 2015, *N.P. Barker & al. 282* (K); Uige Prov., nr Camancoco vill., 857 m a.s.l., 19 Jul 2015, *T. Lautenschläger 40a* (DR048064);

**Benin** (new record): [Plateau Dept.] Adja-Ouèrè, 12 Dec 1901, *G. Le Testu 189* (BM, BR0000013834043);

**Botswana** (new records): [Ngamiland distr.] Maun, 12 Jun 1994, *D.T. Cole 853* (K); Northern Botswana, Selinda Reserve, 30 Mar 2005, *A. Heath & R. Heath 985* (K);

**Burundi** (new records): Bujumbura Rural, Ijenda, 18 May 1969, *J. Lewalle s.n*. (K); Bubanza Territory, 1500 m a.s.l., 11 May 1971, *M. Reekmans 646* (BR0000013837235); Muramvya Prov., 2000 m a.s.l., 18 Jun 1971, *J. Lewalle 5988* (BR0000013837204); [Cankuzo Prov.] Kigamba, 3 Oct 1974, *P. Auquier 4368* (BR0000013837556); [Muramwya Prov.] Teza, 2200 m a.s.l., 15 May 1988, *J. Saintenoy-Simon 120* (BR0000013835798);

**Cameroon** (new records): [Centre Region] Ndelle, [without date] *F. Welwitsch 6544* (BM, K); [Southwest Region, nr Barombi Lake] Johann Albrechtshöhe, 1896, *Staudt 586* (K000243720); Mount Cameroon, 3500 ft, 30 Mar 1952, *C.D. Adams* 6741 (K); Kumba distr., Meninger vill. to Beimiko in Southern Bekundu, 21 Feb 1956, *A. Binuyo & B.O. Deramola 35553* (K); [Central Region] Nanga Eboko town, 13 Mar 1959, *R. Letouzey 1569* (BR0000013834173, K); East Region, Doumé, 12 Nov 1960, *F.J. Breteler 675* (BR0000013834180, FT, K, M, WAG1404293); Far North Region, 10 km W of Fort Foureau, Chari River, 28 Sep 1964, *W.J.J.O de Wilde & B.E.E. de Wilde-Duyfjes 3507* (WAG1404290); [Adamawa Region] Meiganga town, 23 Nov 1964, *G. Amshoff 4007* (BR0000013834166); Tello River, 27 Nov 1964, *W.J.J.O de Wilde & al. 4321* (BR0000013837594); [West Region] Dschang town, 1300 m a.s.l., 6 Dec 1965, *A. Meurillon 206* (BR0000013709037); Yaoundé, Mount Fébé, 1050 m a.s.l, 26 Feb 1977, *J. Lowe 3255* (K); [Adamawa Region] Ranch de Ngaoundaba, 8 Nov 1984, *S. Lisowski 1557* (BR0000013834081); Southwest Region, Mount Kupe, 900 m a.s.l., 7 Dec 1993, *M. Cheek & O. Ebwekoh 5636* (K000051107, WAG1404282); Southwest Region, Nyassosso, 800 m a.s.l., 15 Jan 1995, *M. Cheek & al. 6023* (K000086513); Southwest Region, Mount Kupe, 1000 m a.s.l., 30 Jan 1995, *S. Williams & al. 136* (K000093768, WAG1404132); Southwest Region, Mount Kupe, 7 Sep 1996, *M. Etuge & al. 2681* (K000051092); West Region, Mezam, Bali Ngemba FR, 1400 m a.s.l., 9 Nov 2000, *M. Cheek & al. 10474* (K000746136, K000746128, WAG1404308);

**Central African Rep**. (new records): [Mambéré-Kadéï Pref.] nr Bania, 26 Nov 1965, *A.J.M. Leeuwenberg 7042* (BR0000013834241, K, WAG0185573); [Mambéré Pref.] nr Carnot, 6 Dec 1965, *A.J.M. Leeuwenberg 7227* (WAG0185574); [Mbomou Pref.] Toki, Tondomazouma & Ngalakpa vill., 1 Jan 1970, *A. Morel 262* (P01195352);

**Comoros** (new records): Moroni, May 1830, *Boivin s.n*. (P00196461); Anjouan Island, 1875, *J.M. Hildebrandt 1677* (BM);

**Congo Rep**. (new record): [Niari Region] Bilala, 21 Jun 1989, *R. Dechamps* 13076 (BR0000013834227);

**DR Congo** (new records; selected): Stanley Pool [Pool Malebo nr Kinshasa], 23 Jul 1888, *F. Hens 28* (BR0000013835606, BM, L1673924, WU); [Kongo Central Prov.] Boma, Jul 1895, *A. Dewèvre 133* (BR0000013835446); [Kongo Central Prov.] Kisantu, 1900, *G. Justin 1019* (BR0000013813835590); [Kwilu Prov.] Kolokoso, 16 Jun 1906, *A. Sapin s.n*. (BR0000013835682); [Kongo Central Prov.] Kitobola, 26 Aug 1910, *De Cock 261* (BR0000013835583); Équateur Prov., Zambi, 2 Aug 1913, *Bequaert 549* (BR0000013835439); [Lomami Prov.] Penge, 14 Feb 1914, *Bequaert 2473* (BR0000013837822); Bankana [nr Kinshasa], Jun 1915, *H. Vanderyst 5480* (BR0000013835613); [Bas-Congo Prov.] Ganda-Sundi, 350 m a.s.l., 25 Jul 1919, *V. Goossens 1086* (BR0000013835545); [Kongo Central Prov.] Pangu, Jun 1921, *H. Vanderyst 9554* (BR0000013835699); [Kongo Central Prov.] Matadi, 6 Oct 1930, *H. Vanderyst 25999* (BR0000013835903); [Kongo Central Prov.] Kiduma, Jul 1932, *H. Vanderyst 32223* (BR0000013835620); [Lualaba Prov.] Kapanga, Aug 1933, *F. Overlaet 705* (BR0000013837785); [Haut-Uélé Prov.] Bangadi River, 29 Jan 1937, *A.M. de Graer 917* (BR0000013837846); [Kasai Central Prov.] Tshimbulu, 21 Apr 1937, *F. Matagne 311* (BR0000013835651); [North Kivu Prov.] Rutshuru, 1937, *J. Lebrun 8872* (K); [North Kivu Prov.] Rutshuru, 25 Feb 1938, *J. Ghesquière 6023* (BR0000013836399); [Tshopo Prov.] Yangambi, 470 m a.s.l., 9 Aug 1938, *J. Louis 10753* (BR0000016287112, K); [Tshuapa Prov.] Lofoli vill., Jul 1939, *P. Quarré 5717* (BR0000013837433); [Tshopo Prov.] nr Lileko, 28 Feb 1940, *R. Ofermain 208* (K); [Haut-Uélé Prov.] Nambiongo vill., Apr 1940, *J.W.G. Wyld 689* (BM); [Orientale Prov.] Nioka, 12 Jul 1945, *R. Germain 3968* (BR0000013836047); [Equateur Prov.] Boyeka, 19 Nov 1946, *J. Leonard 1030* (BR0000013837419, K); [Haut-Lomami Prov.] Kaniama, 870 m a.s.l., Aug 1947, *W. Mullenders 1224* (BR0000013837815); [Ituri Prov.] Mt. Irumu, Jun 1949, *R. Germain 5189* (BR0000013836054, FT); South Kivu Prov., Kabare, 11 Mar 1951, *R. Pierlot 90* (K); Orientale Prov., Garamba NP, Mar 1952, *G. Troupin 403* (BR0000013837853, K); N’kolo, 26 Aug 1953, *R. Devred 1356* (BR0000013835576); Haut-Katanga Prov., Zawa Mt., 25 Jun 1954, *J. Delvaux 563* (BR0000013837860); [Ituri Prov.] Djugu [Territory], 15 Mar 1957, *A. Froment 91* (BR0000013836030, K); [North Kivu Prov.] Walikale town, Jul 1957, *R. Gutzwiller 1268* (BR0000013836450, M); [North Kivu Prov.] Mutongo, 1 May 1958, *R. Gutzwiller 2650* (BR0000013835910); [Kongo Central Prov.] Lemba to Mayumbe, 27 Aug 1966, *H. Breyne 257* (BR0000013835521); [South Kivu Prov.] Lwiro, 1700 m a.s.l., 29 Dec 1971, *J. Lambinon & G. Troupin 2061* (BR0000013837426, FT, K); [Kongo Central Prov.] Banana [town], 3 Jul 1974, *L. Pauwels 5233* (BR0000013835491, WAG1404154); Virunga NP, 16 Sep 1974, *J.-P. d’Huart 58* (BR0000013836146); [South Kivu Prov.] Kilimbwe, 16 Nov 1977, *T. Yamada 171* (K); [South Kivu Prov.] Lubimbi, 9 May 1978, J. *van Gysel 20* (BR0000013836719); [Tshopo Prov.] Kisangani, 21 Dec 1978, *J. Lejoly 4490* (BR0000013835729); Bas-Uélé Prov., Bambesa Terr., Dindila vill., 30 Jan 1988, *F. Szafranski 1391* (BR000001383535743); [Kiwu Prov.] Kikwit, 7 Nov 1990, *B. Masens 46* (BR0000013835736); [Ituri Prov.] 60 km N of Mambasa, 1000 m a.s.l., mid-1990, *H. Terashima 628* (BR0000013835989);

**Equatorial Guinea** (new records): Fernando Po [Bioko Island], 1860, *anonymous s.n*. (K); [Bioko Island] Musola, 10 Jan 1947, *E. Guinea 1147* (MA-01-00698962); Annobón Island, 890 ft, 25 Jul 1959, *F. Melville 192* (BM, K); Bioko [Island], 770 m a.s.l., 13 Feb 1989, *J. Fernández Casas 11582* (BR0000013837570, K); [Rio Muni Region] Temelon, 9 Nov 2000, *R. Pérez Viso 4139* (K);

**Ethiopia** (new records): [Amhara Region] Tana Lake, 14 Nov 1937, *R. Pichi-Sermolli 1437* (FT, K); [Southern Nations, Nationalities, and Peoples Region] Sagan [Segen town], 28 Jul 1939, *R. Corradi 3214* (FT); Gamu-Gofa Region, Between Soda & Arba Minch, 1240 m a.s.l., 28 Sep 1975, *P.C.M. Jansen 3777* (BR000000565821);

**Gabon** (new records): [Ngouni Prov.] Doussala, 100 m a.s.l., 23 Sep 2000, *H.P. Bourobou Bourobou & al. 452* (WAG0199843); Ogooué-Maritime Prov., Loango NP, 19 Mar 2006, D.*J. Harris & al. 8704* (E00217301, WAG1404159);

**Ghana** (new records): nr Kibi, Birim River, 2370 ft, 16 Dec 1952, *J.K. Morton 8137* (K); [Eastern Regio] Asiakwa to Pusupusu, 19 Dec 1952, *J.K. Morton 8218* (K);

**Guinea** (new records): [Kindia Region] Kolante, 10 Nov 1938, *J. Chillou 1035* (BR0000016286443); [Nzérékoré Region] Simandou Range, 1065 m a.s.l., 29 Nov 2008, *P.K. Haba & al. 483* (K000460820);

**Ivory Coast** (new records): [Bélier Region] between Toumodi & Yamoussoukro, 7 Jan 1954, *L. Aké Assi 7260* (UCJ000581); [Gbêkê Region] Beoumi, 15 Dec 1955, *L. Aké Assi 3515* (UCJ000582);

**Kenya** (new records): [Rift Valley Prov.] Narok distr., Mara River, 5200 ft, 3 Apr 1961, *P.E. Glover & al. 399* (BR0000013835842, K); [Nyanza Prov.] North Kavirondo distr., Kakamega forest, 1500 m a.s.l., 21 Mar 1977, *S.S. Hooper & C.C. Townsend 1508* (K); [Taita-Taveta County] Ngangao Forest, 1750 m a.s.l., *M. Wakanene 782* (W0333202); Makueni distr., Kibwezi Forest Research, 980 m a.s.l., 21 Nov 2010, *Q. Luke & P. Luke 14895* (K);

**Lesotho** (new record): Leribe [Hlotse], 1914, *Dieterlen 986* (K, BM);

**Madagascar** (new records): nr Antatanarivo, Oct 1880, *J.M. Hildebrandt 4108* (BM, K, LE, M, W0333222); [Anosy Region] Berenty Reserve, Oct 1985, *S. O’Conner 108* (K); Tulear Prov., nr Betioky, Beza Mahafaly Reserve, 13 May 1987, *P. Phillipson s.n*. (BR0000013835408, K, WAG1040296); Antsiranana Region, Befalafa, 400 m a.s.l., 13 Jun 1998, *L. Gautier & al. 3351* (K, WAG1404398); Finarantsoa Prov., Ranomafana NP, 20 Nov 1998, *F. Almeda 8211* (K);

**Malawi** (new records): [Southern Region] Mulanje, 1919, *C. Shinn s.n.* (BM); [Southern Region, Thyolo distr.] Cholo Mountain, 1300 m a.s.l., 20 Sep 1946, *L.J. Brass 17661* (BM, BR0000013837730, K); [Southern Region] Blantyre, 4 Oct 1960, *J. Chapman 936* (K); Central Region, Dedza distr., 12 Jul 1961, *J. Chapman 1427* (K); Central Region, Lifidzi Breeding Center, 21 May 1985, *I.H. Patel & W. Nachamba 2196* (BR0000013835194); Southern Region, Soche Mt., 2 Aug 1985, *A.J. Salubeni & W. Nachamba 4271* (K); Central Region, Lilongwe Nature Sanctuary, 25 Jun 1987, *A.J. Salubeni & E.J. Tawakeli 4964* (K);

**Mayotte** (French Overseas Department; new record): Grande-Terre. 26 May 2005, *F. Barthelat & al. 1491* (K, P00631148);

**Mozambique** (new records): nr Sena, Zambezi River, Oct 1883, *Kirk s.n*. (K); [Nampula Prov.] Marenga, Mar 1892, *Menyharth 1179* (WU); [Manica Prov.] Garuso forest, Apr 1935, *H.B. Gilliland 1805* (BM, K); [Maputo Prov.] Salamanga [town], 7 Jun 1948, *A.F. Gomes de Souza 3736* (FT, K, M); [Manica Prov.] 10 miles N of Vanduzi, 1900 ft, 27 Apr 1962, *N.C. Chase 7694* (BM, K); Cabo Delgado Prov., 20 km NE of Ancuabe, 18 Aug 1983, *E.M.C. Groenendijk 623* (BR0000013835262); Zambezia Prov., Chiperone Mt., 29 Nove 2006, *H. Patel & J. Bayliss 7179* (K000545142); Zambezia Prov., Mabu Mt., 18 Oct 2008, *Mphamba & al. 81* (K); [Sofala Prov.] Muanza distr., Gorongosa NP, 112 m a.s.l., 4 May 2013, *B. Wursten 703* (BR0000027318294V); Manica Prov., Moribane Forest Reserve, 26 Jun 2015, *M. Cheek & al. 17900* (K);

**Namibia** (new records): [Otjozondjupo Region] Waterberg, 5 Feb 1939, *O.H. Volk 1054* (M); [Kavango East Prov.] nr Rundu, 10 May 1939, *O.H. Volk 1960* (M); [Kunene Region] nr Etanga, 4 Apr 1957, *B. de Winter & O.A. Leistner 5439* (K, M); [Omusati Region] Ruacana Falls, 29 Apr 1962, *T.F. Kotze 1714* (K, M); Windhoek, 26 Mar 1963, *R. Seydel 2217* (M);

**Nigeria** (new records): Oban distr., 1911–1912, *P.A. Talbot 2311* (BM, K); nr Ibadan, 600 ft, Oct 1936, *R. Newberry & al. 139* (K); Ondo Prov., Akure division, 5 Jan 1948, *J.P.M. Brenan & E.W. Jones 8726* (BM, K, M0241526); [Taraba State] Kamatan Forest Reserve, 5 Dec 1968, *B.O. Daramola 62483* (K); [Cross River State] Nyaje to Mkpot, 5 Mar 1973, *Latilo & Oguntayo 70501* (K);

**Rwanda** (new records): [Southern Prov.] Kabgayi, 1800 m a.s.l., 14 Mar 1970, *G. Bouxin & M. Radoux 1550* (BR0000013837518); [Southern Prov.] Butare town, 19 Jul 1974, *P. Auquier 3244* (BR0000013837457); [Western Prov.] Bweyeye, 17 Aug 1974, *P. Auquier 3814* (BR0000013837471); [Northern Prov.] nr Ruhengeri, 1850 m a.s.l., 9 Oct 1974, *P. Auquier 4438* (BR0000013837495, WAG1404304); nr Kigali, 13 Oct 1974, *P. Auquier 4560* (BR0000013837501); Kigali, 1500 m a.s.l., 11 Aug 1984, *J. Lejoly 220* (BR0000013837068);

**São Tomé & Príncipe** (new records): São Tomé, 1885, *A. Moller 684* (BM); Guadalupe, 17 Jul 1956, *T. Monod 11893* (BM); [Principe Island] Santo Antonio, 17 Jul 1990, *V. Sequeira 7* (K);

**Sierra Leone** (new record): Loma Mountains, 29 Dec 1963, *J.K. Morton 355* (K);

**Somalia** (new records): [Middle Shebella Region] Balad town, 28 Dec 1919, *G. Scassellati 13* (FT); [Lower Shabelle Region] 25 km S of Merca town, Gungi vill., 12 Sep 1975, *R. Ravazzano 1166* (FT);

**South Africa**: see type of *A.acuminata*; nr Durban, 14 May 1895, *J. Wood 7203* (K); Vet River [Vetrivier] [without date] *Burke s.n*. (K); [North-West Prov.] Hakboslaagte, 28 Feb 1943, H. *Klinges 1667* (M); [KwaZulu Natal Prov.] Hawaan Forest, 100 ft, 16 Mar 1966, *E.J. Moll 3115* (K); [KwaZulu-Natal Prov.] Mtubatuba, 12 May 1970, *R.G. Stray 9777* (K); [KwaZulu-Natal Prov.] Ubombo, 15 Feb 1976, *J.P.M. Brenan 14275* (K); Transvaal Prov., Silverton, 16 Feb 1979, *A. Balsinhas 3379* (K); [Eastern Cape Prov.] nr Alice town, 1800 ft, 16 Mar 1985, *P.B. Phillipson 1053* (K); nr Durban, 29.8612277°S, 30.979105555°E, *E. Douwes* (Fig. 5B);

**Tanzania** (new records): [Zanzibar] Pemba [Island], Ngezi Forest, [without date] *G.H. Vaughan 552* (K); [Kilimanjaro Region] Marangu, 1560 m a.s.l., Nov 1893, *G. Volkens 1390* (K); [Mbeya Region] Kyimbila distr., 22 Jun 1911, *A. Stolz 778* (K, M, L1673914); [Morogoro Region] Mchombe, 28 Sep 1931, *anonymous 357* (FT); [Kilimanjaro Region] Lyamungo, 21 Aug 1932, *P.J. Greenway 3065* (K); Tanga Region, Pangani, 20 Jul 1955, *R. Tanner 1974* (BR0000013834876, K); [Morogoro Region] nr Mgeta vill., 980 m a.s.l., 17 Jul 1958, *A. Gilli 155* (W0333233) together with *A.porphyrostachya*; [Kigoma Region] Kabesi, 4 Sep 1958, 4500 ft, *J. Newbould & T.G. Jefford 2323* (K); Zanzibar [Unguja Island] Marahubi, 9 Jul 1963, *H.G. Faulkner 3209 & 3258* (BR0000013837716, K); Arusha Region, Tengeru, 10 Mar 1968, *R. Ravazzano 16* (FT); [Tanga Region] Lushoto distr., 19 Apr 1968, *S.A. Renvoize 1592* (K); [Morogoro Region] Morogoro town, 1800 ft, 19 Jul 1969, *B.J. Harris 2971* (K); Kibiti [distr.] 150 km on Dar–Kilwa road, 19 Jun 1970, *A.N. Minjas & M.D.I. Raya 1900* (BR0000013835064); Lushoto distr., Buhurula, 10 Jul 1970, *Shabani 581* (BR0000013835057); Masai distr., nr Engaruka vill., 3600 ft, 11 Feb 1971, *H.M. Richards & S. Arasululu 26645* (BR0000013834944, K); [Morogoro Region] Makanga camp, nr Shiri River, 16 May 1986, *anonymous 1747* (K); Tanga Region, East Usambara Mts., Kwamkoro FR, 930 m a.s.l., 27 Oct 1986, *S.T. Iversen & al. 86220* (K); Coast Region, [Rufiji distr.] Mchungu forest, 4 Aug 1990, *anonymous 1203* (K); Tanga Region, Gendagenda Forest, 200 m a.s.l., 31 Jul 1991, *Frontier Tanzania team 2377* (K); [Lindi Region] Litipo forest, 25 Jul 1993, *L. Mwasumbi & P. Clarke 3621* (K); Kagera, Bukba distr., Mabuye vill., 1166 m a.s.l., 29 Apr 2001, *L. Festo & al. 1460* (K); Kilimanjaro Region, nr Machame vill., -3.219119, 37.222290, ca. 2000 m a.s.l., 15 Jun 2021, *A.P. Sukhorukov s.n*. (MW);

**Togo** (new record): [Kara Region] Baga, 19 Dec 1985, *Koumantega s.n.* (TOGO01602);

**Uganda** (new records): Ruwenzori Expedition, 1893–1894, *G.F. Scott Elliot 7248* (K); [Central Region] Mulange [Hill], 4000 ft, Sep 1920, *R.A. Dummer 4480* (K); nr Kampala, Sep 1931, *anonymous 2210* (K); Budongo, Nov 1935, *W.J. Eggeling 2286* (BM); [Central Region] Wabusana, 28 Jul 1956, *L. Brown 2252* (K); Rabongo, Kibale NP, 4800 ft, 26 Jun 1957, *L. Brown 66* (BR0000013834463); Kampala, 2 Sep 1960, *R. Kendall & J. Richardson 60* (K); [Mpigi distr.] Mpanga forest, 2 Sep 1974, *A.B. Katende 2316 & 2318* (K); [Bwindi NP] Rushaga [sector], 5 May 1992, *A.B. Cunningham & Mihanda 4045* (K); Kabalaga [Murchison] NP, 5 Oct 1992, *D. Sheil 1393* (K); Murchinson Falls NP, Rabongo forest, 7 May 1993, *D. Sheil 1495* (K);

**Zambia** (new records): Southern Prov., Machipapa, 9 Mar 1962, *W.L. Astle 1458* (K); [Copperbelt Prov.] Kataba, 3 Jul 1963, D.B. Fanshawe 7901 (K); [Copperbelt Prov.] Kitwe, 4 Feb 1964, *J.M. Mutimushi 576* (K); [Lusaka Prov.] Luangwa Game Reserve, 7 May 1965, *B.L. Mitchell 2889* (K); North Luangwa NP, 22 Apr 1994, *P.P. Smith 518* (K);

**Zimbabwe** (new records): [Manicaland Prov.] Chirinda Forest, 3700–4000 ft, 1906, *C.F.M. Swynnerton 1510* (BM); [Manicaland Prov.] Chiringa Forest, 3600 ft, Jun 1967, *B. Goldsmith 77* (BR0000013837747, K); Victoria Falls, Jul 1908, *Schwarz 13405* (BOL0291974).

***Achyranthesannua* Dinter**

**Africa**

**Angola** (new records): [Lunda Norte Prov.] nr Bumba, [without date, probably late 19^th^ century] *F. Welwitsch 6573* (BM); [Cuanza Norte Prov.] Golungo Alto [town], [without date, probably mid-19^th^ century], *F. Welwitsch 6547* (BM – right-handed specimens); nr Kunere River between Kiteve and Humbe, 3 Jun 1900, *H. Baum 961* (K0002437271, M); Huila Prov., Hungueria, 1300 m a.s.l., 10 Jun 1937, *A.W. Exell & F.A. Mendonça 2448* (BM); [Cunene Prov.] nr Humbe, 1100 m a.s.l., 10 Jun 1937, *A.W. Exell & F.A. Mendonça 2889* (BM); Huila Prov., Lubango, 2220 m a.s.l., 22 Apr 1960, *E.J. Mendes 3817* (BR0000013835088, M, P05047982); Huila Prov., Sá da Bandeira [Lubango], 4 May 1965, *C. Henriques 371* (K); [Namibe Prov.] Bruco, 14 May 1965, *C. Henriques 412* (BM); [Cunene Prov.] Xangongo to Mujombe vill., 18 Mar 1989, J.*M. Daniel & al. s.n.* (FT);

**Benin** (new records): [Borgou Dept.] Kalalé, Sep 1987, *M. Kreis 98* (BR000000547056); [Borgou Dept.] Sakabansi [town], 5 Nov 1987, *J. Lejoly 527* (BR0000005470266); [Borgou Dept.] Kalalé town, 15 Oct 1988, *J. Lejoly 146* (BR000000547046); Zou Dept., Savalou, 19 Nov 1998, *L.J.G. van der Maesen 6511* (WAG1404341); Atakora Dept., Kouandé, 22 Oct 2001, *A. Akoegninou & al. 5601* (WAG1404334); [Borgou Dept.] Tchaourou, 22 Sep 2005, *P. Houngnon 7169* (WAG1404338); Ailbori Dept., Banikoara, 26 Sep 2007, *K. Jurisch 141* (FR-0009269);

**Botswana** (new records; selected): [Kgatleng distr.] Mochudi, 1914, *C.C. Harbor 6599* (BOL0291967, K); Ngamiland [North-West distr.], Mwakupan vill., nr Ngami Lake, 7 Mar 1969, *R.J. de Hoogh 126* (WAG1404397); [Ghanzi distr.] Ghanzi pan, 11 Mar 1970, *M.C. Cole 8798* (K); Moremi Wildlife Reserve, Okavango swamps, 3 Mar 1972, *H. Biegel & G. Russell 3841* (K, M); [Ngami distr.] nr Maun, 7 Apr 1975, *P.A. Smith 1340* (K); nr Gaborone, 31 Mar 1977, *O.J. Hansen 3102* (K); Kalahari, 23°05'S, 20°46'E, 21 Mar 1980, *C. Skarpe 422* (K); Ngamiland distr., Aba Hills, 1110 m a.s.l., 20 Mar 1987, *D.G. Long & D.A.H. Rae 392* (K); Selinda Reserve, 26 Mar 2005, *A. Heath & R. Heath 975* (K);

**Burkina Faso** (new records; selected): nr Ouagadougou, 6 Nov 1976, *L. Aké Assi 13492* (UCJ000583); [Balé Prov.] Boromo, 13 Nov 1980, *J. Lejoly 12* (BR0000013833992); Ouagadougou, 31 Aug 1982, *J. Lejoly 209* (BR0000013834005); [Oubritenga Prov.] Gampela, 14 Sep 1982, *J. Lejoly 498* (BR0000013834012); Tapoa Prov., Maadaga, 12 Sep 1991, *R. Martin 241* (FR-0010160); Tapoa Prov., Diapaga to Tansarga, 14 Sep 1991, *K. Hahn 517* (FR-0010164); Boulgou Prov., 7 km NW of Tenkodogo, 7 Nov 1991, *U. Kéré 434* (FR-0010165); [Kompienga Prov.], Pama, 28 Oct 1993, *M. Ataholo 329* (FR-0017535); Seno Prov., Djigo, 23 Sep 1994, *K. Küppers 1302* (FR-0010171); Oudalan Prov., Menegou vill., 250 m a.s.l., 21 Sep 1996, *J.E. Madsen 5766* (WAG1404197); Oudalan Prov., Kissi, 7 Sep 1999, *J. Müller 337-5* (FR-0010173); Kossi, Nauna, 2 Oct 2004, *B. Bako & J. Boussim 386* (K); Yatenga [Prov.], N of Ouahigouya, 16 Aug 2005, *L.J.G. van der Maesen & al. 8277* (WAG0203842); Tapoa Prov., Kaabougou, 26 Sep 2007, *A. Zwarg 45* (FR-0025046);

**Burundi** (new records): Bururi Prov., 1850 m a.s.l., 2 Jun 1979, *M. Reekmans 8151* (L3709558); Bubanza town, 1150 m a.s.l., 3 Jun 1980, *M. Reekmans 9279* (BR0000013708610, K);

**Cameroon** (new records): [Northwest Region] Bamenda, 14 Jan 1928, *F.W.H. Migeod 302* (K, M); [Northwest Region] Bambui, 1 Jan 1951, *R.W.J. Keay 28355* (K000025623); [Northwest Region] Bamenda, 1 Jan 1951, *R.W.J. Keay 28335* (M); [Far North Prov.] 15 km N of Mokolo town, 18 Sep 1964, *M. Binolong 144* (K); [Far North Prov.] 25 km WNW of Fort Foureau [Kousséri], 28 Sep 1964, *R. Letouzey 7101* (K); [Far North Prov.] nr Guirvidig, 10 Oct 1964, *R. Letouzey 7247* (K); West Region, Bafou, 2 Dec 1969, *anonymous 1732* (K); Mogode town, 1000 m a.s.l., 8 Oct 1972, *A.J.M. Leeuwenberg & W.E.K. van Beek 10414* (BR0000013834104, WAG1404365); [Adamawa Region] Léré, Oct 1996, *C. Milan 6* (BR0000013834074); Southwest Region, Kodmin vill., 1400 m a.s.l., 20 Jan 1998, *Ghogue & al. 68* (K000051118); Northwest Region, Mantoum vill., 1560 m a.s.l., 4 Oct 2001, *B.J. Polard 609* (K000875617); [Northwest Region] Mezam, 11 Apr 2004, *M. Etuge & al. 5367* (K);

**Cape Verde** (new records): Santiago Island, 8 km of Praia, 190 m.s.l., 29 Nov 1955, *L.A. Grandvaux Barbosa 5749* (BM);

**Central African Republic** (new record): 6 km N of Gounda town, 10 Feb 1982, *J.M. Fay 3667* (K);

**Chad** (new records): [Batha Region] Ouadi-Rimé, 10 Sep 1962, *H. Gillet s.n.* (P00958623); [Tandjilé Ouest Dept.] road Kélo to Pala, 3 Sep 1984, *S. Lisowski 377* (BR0000016286498); N’Djamena, 23 Dec 1984, *S. Lisowski 1652* (BR0000016286504);

**Djibouti** (new records): [Arta Region] Ras Korali, 9 Mar 1893, *A. Terracciano 10* (FT); French Somalia [Tadjourah Region], Day vill., 1400 m a.s.l., 4 Jun 1953, *E. Chedeville 327 & 360* (FT); French Somalia [Randa distr. [Randa [vill.], 900 m a.s.l., 9 Jan 1956, *E. Chedeville 1039* (FT); ibidem, May 1959, *E. Chedeville 1340* (FT);

**DR Congo** (new records; selected): [South Kivu Prov.] Uvira, 1908, *J. Rouling s.n*. (BR0000016287488); [nr Kinshasa] Bankana, Jun 1915, *P.H. Vanderyst & P.J. Lambrette 5493* (BR0000016287006); [South Kivu Prov.] Panzi vill., 1925, *E. Vanderyst 17207* (BR0000016287044); [Haut-Katanga Prov.] Karavia [nr Lubumbashi town] Jun 1929, *P. Quarré 1761* (BR0000013708009); [Haut-Katanga Prov.] Katuba, Jun 1933, *P. Quarré 3223* (BR0000013708016); [Bas-Uélé Prov.] Bambesa territory, Apr 1934, *H. Brédo 1220* (BR0000016287105); [Haut-Katanga Prov.] Elizabethville [Lubumbashi], 1937, *Salésiens 318* (BR0000013708122); [Haut-Katanga Prov.] Kasenga town, May 1939, *H. Brédo 2916* (BR0000013836764); Haut-Uélé Prov., N’ganga [Ganga], 14 Dec 1944, *Tecno 88* (BR0000013836092); [Kasai-Occidental Prov.] Mwango, 6 Jun 1947, *G. Kevers 129* (BR0000016287051); [Lualaba Prov.] Upemba NP, Apr 1948, *G.F. de Witte 3706* (L1695061); [Haut-Uélé Prov.] Faradje, 19 Dec 1949, *J. Costermans 30* (BR0000016287167); [Tanganyika Prov.] nr Manono, Kiala, Jul 1957, *A. Thiebaud 665-39* (BR0000013835705); [North Kivu Prov.] Rutshuru, 10 Dec 1958, *G. Troupin 8928* (BR0000013836672); South Kivu, Katana, 1959, *Cambridge Congo Expedition 11* (BM); Haut-Katanga Prov., Kasenga Territory, Kaselele, 890 m a.s.l., 19 May 1960, *M. Streel 847* (BR0000013837877); South Kivu Prov., Kabare Territory, 2300–2400 m a.s.l., 30 Dec 1969, *H. Ern 25* (BR0000013836344); [Haut-Katanga Prov.] Likasi, 13 Jul 1977, *R. Wechuysen 385* (BR0000013708993); Madimba Territory, 18 Jul 1981, *Nkunga 6488* (BR0000016286986);

**Egypt**: see lectotype of A.argenteavar.viridescens; [Halaib Triangle] Gebel [Gabal] Elba, 18 Mar 1928, *Khattab 6305* (K); [Halaib Triangle] Gebel [Gabal] Elba, 24 Jan 1933, *J.R. Shabetai 12* (K);

**Eritrea** (new records; selected): [Northern Red Sea Region] Dahlak Island, Mar 1892, A. *Terracciano 792* (FT); [Northern Red Sea Region] Saati vill., 8 Mar 1893, *V. Ragazzi & A. Pappi s.n.* (FT); [Northern Red Sea Region] Damas valley, 14 Apr 1893, *A. Pappi 4106* (FT); Dogali vill. [nr Massaua town], 2 Mar 1902, *A. Terracciano & A. Pappi 18* (FT); [Debub Region] Mai Daro [vill.] 25 Sep 1902, *A. Pappi 3041* (FT); Keren town, Nov 1902, *A. Tellini 930* (FT); [Northern Red Sea Region] Sabar Guma, 1903, *A. Tellini 1566* (FT); [Dek’emhare] Hamasen, Ghinda, 960 m a.s.l., 14 Jan 1909, *A. Fiori s.n.* (FT001027); [Northern Red Sea Region] Samhar, 10 Apr 1910, *A. Pappi 8654* (FT); [Anseba Region] Karkabet town, 1915, *I. Baldrati 1137* (FT); [Northern Red Sea Region] between Massaua & Ghinda, 26 Mar 1938, *Bologna 22* (FT); [Northern Red Sea Region] Archico [Arkiko], 17 Jan 1952, *H.B. Gilliland & W. Stower 4094* (K); Senafi to Asmara, 16 Augt 1973, *G. Aweke & M.G. Gilbert 673* (WAG1404374); Asmera town, 2350 m a.s.l., 5 May 1988, *O. Ryding 1177* (K);

**Eswatini** (new records): Mbabane, 23 Mar 1956, *R.H. Compton* 25825 (K); -26.235145°S, 31.178688°, 17 May 2018, *K. Braun*; image available at: https://www.gbif.org/ru/occurrence/4901375172;

**Ethiopia** (new records): [Semien Gondar Zone] nr Matemma [Metema], 1865, *G. Schweinfurth 646* (BM); [South West Region, Telo distr.] Kolla, 1863–1868, *Schimper 188* (BM);] Oromia Region] Neghelli town, 1937, *G. Cufodontis 250* (FT); [Sidamo Region] Ruscello di el Dire, 15 May 1939, *R. Corradi 3323* (K); [Southern Nations, Nationalities, and People’s Region] Asile, 30 Jul 1939, *R. Corradi 3349 & 3350* (FT); [Somali Region, Dollo Zone] Bulleh Sirrauw, 21 Nov 1944, *P. Glover & H. Gilliland 377* (BM); [Oromia Region] Agheremariam [Bule Hora], 1 Dec 1952, *J.B. Gillett 14524* (M); [Oromia Region] nr Jimma town, 11 Feb 1956, *R.B. Stewart s.n.* (FT, K); [Oromia Region] Adami Tullu town, Sep 1956, *H.R.D. Sanford 8* (FT); [Oromia Region] nr Dembidolo town, 6300 ft, 2 Mar 1957, *H.F. Mooney 6860* (FT); [Oromia Region] 25 km E of Lekemti, 2000 m a.s.l., 1 Jul 1965, *W.J.J.O de Wilde & B.E.E. de Wilde-Duyfjes 7179* (WAG1404384); 60 km S of Addis Ababa, 2800 m a.s.l., 30 Oct 1965, *W.J.J.O de Wilde & B.E.E. de Wilde-Duyfjes 8621* (BR0000013834395); [Afar Region] 50 km W of Awash station, 800 m a.s.l., 8 Apr 1966, *W.J.J.O de Wilde & B.E.E. de Wilde-Duyfjes 10678* (BR0000013834371, K, WAG1404376); [Harari Region] nr Harar, ca. 1700 m a.s.l., 18 Mar 1968, *E. Westphal & J.M.C. Westphal-Stevels 3506* (BR0000013834319, WAG0185680); Blue Nile Gorge, 1700 m a.s.l., 25 Sep 1969, *C. Parker 339* (K); Kaffa Prov., 8 km S of Jimma, 1700 m a.s.l., 8 Nov 1970, *I. Friis & al. 179* (BR000001384333); 7 km on road Worata – Debre Tabor, 31 Dec 1972, *C.J.P. Seegeler 3043* (WAG1404368); [Afar Region] nr Awash, 900 m a.s.l., 26 Sep 1976, *L. Boulos 9360* (FT); Shewa Prov., Omo River, 900 m a.s.l., 11 Oct 1984, *R.E. Gereau 1231* (K);

**Gambia** (new record): Yundum town, 29 Oct 1974, *P.J. Terry 3192* (K);

**Ghana** (new records): [Bono Region] between Band & Menji vill., 23 Dec 1953, *J.K. Morton 25242* (K); Larabanga [town], 12 Oct 1959, *R. Rose Innes 1414* (K);

**Guinea** (new records): nr Conakry, Jun 1913, *Ch. D’Allerizette s.n*. (L1673910); [Mamou Region] Pita, 17 Oct 1956, *J.G. Adam 12548* (P00695162);

**Guinea-Bisau** (new record): [Gabu Region] Madina do Boé, 2 Feb 1962, *J. Alves Pereira 2948* (MA778959);

**Kenya** (new records): [Kisumu County] Muhoroni, 26 Sep 1950, *Haggie & Turbo 7988* (K); 10 km SE of Meru, Giaki Farm, 3700 m a.s.l., 16 Aug 1958, *A. Bogdan 4628* (K); 15 km N of Kilifi, 17 Sep 1958, *J.C. Moomaw 888* (K); nr Maralal town, 7 Oct 1960, *D.R.M. Stewart 358* (K); Narok distr., Narosura, 6200 f, 16 Aug 1961, *P.E. Glover & al. 2427* (K); Enekidongo, 3000 ft, 16 Jun 1962, *P.E. Glover & al. 2831* (K); [Narok Region] Kipleleo, 21 Jun 1962, *P.E. Glover & Samuel 2875* (FT, K); [Rift Valley Prov.] nr Nakuru town, 6000 ft, 4 Aug 1967, *O.M. Mwangangi 70* (FT, K); Kwale distr., Shimba Hills, 1450 ft, 20 Feb 1968, *F. Magogo & P.E. Glover 153* (BR0000013834548, FT, K); Kitui distr., Uthekethe, nr Mutomo, 1050 m a.s.l., 12 Aug 1968, *Z.J. Kimani 40* (BR0000013834525, K); South Turkana, Loriu Plateau, 4000 ft, 2 Jun 1970, *B. Mathew 6583* (K); Nyanza Prov., Kisii distr., between Ikoba & Ogembo, 1750 m a.s.l., 29 Oct 1974, *D. Vuyk & F.J. Breteler 44* (BR0000013834562, K, WAG1404174); Masai distr., 23 km E of Oloitokitok, 10 Mar 1977, *S.S. Hoopeer & C.C. Townsend 1290* (K); N of Ngomeni, 720 m a.s.l., 25 May 1977, *J.B. Gillett 21087* (FT, K); Nanyuki distr., 18 km NE of Nanyuki, 1850 m a.s.l., 22 Oct 1977, *M.G. Gilbert 4881* (K); Nyanza Prov. [Kisii County], 9 km E of Kisii town, 23 Mar 1978, *A.C. Plaizier 1067* (WAG1404125); Kibwezi–Kitui road, 900 m a.s.l., 26 May 1979, *M.G. Gilbert 5408* (K); Nairobi to Magadi, 1400 m a.s.l., 17 May 1981, *M.G. Gilbert 6145* (K); [Rift Valley Prov.] Trans-Nzoia distr., 1800 m a.s.l., 1988, *G.M. Mungai 520* (K); Rift valley, Bogoria Lake, 1000 m a.s.l., 15 Aug 1988, *P. Pilsl 9156* (HPILSL 009156); Tana River distr., Tana River National Primate Reserve, 16 Mar 1998, *D. Luke & al. 464* (K);

**Lesotho** (new records): Leribe [Hlotse], [without date and collector] *41* (K); “Basutoland”, 1913, *Dieterlen s.n.* (K);

**Liberia** (new record): [Northern Nimba County] Yekepa vill., 14 Dec 1964, *J.G. Adam 20071* (M);

**Madagascar** (new records): Central Plateau, 1914, *H/T. Hodgkin & C.E. Stansfield 134* (K); [Prov. of Toamasina] Périnet [Andasibé], 14 Dec 1985, *A.J.M. Leeuwenberg 13810* (BR0000013835316);

**Malawi** (new records; selected): [Northern Region] Karonga, 28 Jul 1952, *Williams s.n*. (BM); Northern Prov., Chilumba, 22 Apr 1969, *J. Pawek 2285* (K); Nyika Plateau, 7500 ft, 30 Aug 1969, *W.M. Fitzpatrick 77* (BM); Southern Region, Lengwe Game Reserve, 9 Mar 1970, *R.K. Brummitt & A.J. Hall-Martin 8978* (K); [Northern Region] Mzuzu town, 4500 ft, 2 Jul 1974, *J. Pawek 8781* (BR0000013835187, WAG1404184); Northern Region, Chikangawa, 5900 ft, 14 Jul 1978, *E. Phillips 3556* (K, WAG1404389); Zomba distr., Zomba Plateau road, 8 Aug 1978, *H.G. Msiska 51* (BOL0291976); Southern Region, Zomba distr., 24 Jul 1984, *K. Kaunda & E.J. Tawakali 93* (K);

**Mali** (new record): [Tombouktou Region] Timbuktu, 27 Aug 1927, *O. Hagerup 284* (BR0000013833947, K);

**Mauritania** (new records): [Gorgol Region] Kaedi, 1972, *A. Naegele 188* (K); [Gorgol Region] Rinndiao, 16 Oct 1972, *A. Naegele 160* (K); Sixth Region, Hassei Ould Babouh, 20 Nov 1975, *D. Dupont 126* (BR0000016286351); Assaba Region, Kankossa distr., El Awje, 9 Sep 2008, *J.-N. Labat & al. 3970* (P00577631);

**Mozambique** (new records): [Nampula Prov.] Boroma River, Marenga, Apr 1892, *Menyharth 666* (WU); Cabo Delgado, 9 Apr 1964, *A.R. da Torre & Paiva 11811* (BR0000013837754, WAG1404328); Tete Prov., Cabora Bassa, 21 Apr 1972, *A. Pereira & M.F. Correia 2192* (WAG1404244); [Manica Prov.] nr Chimoio town, 710 m a.s.l., 22 Oct 2013, *B. Wursten 814* (BR0000027322529V); Sofala Prov., Gorongosa NP, 17 May 2015, *B. Wursten 15160* (BR0000027330548V);

**Namibia** (selected): see neotype of *Achyranthesannua*; [Otjozondjupa Region] Omuramba, Otjenga, 14 Mar 1939, *O.H. Volk 1405* (M-0293179); Kavango-East Region] nr Rundu, 8 May 1939, *O.H. Volk 1871* (M); [Khomas Region] Khomas Highland, 18 May 1939, *G. Gassner 194* (M); Okavango-Nord, 28 Jun 1939, *O.H. Volk 2356* (M); [Hardap Region] Rehoboth, 10 Apr 1949, *K.G. Strey 2534* (K); [Otjozondjupa Region] Grootfontein, 30 Mar 1953, *H. Klinges 2863* (M); [Oshikoto Region] Tsumeb, 29 Mar 1955, *B. de Winter 2990* (K, M); [Hardap Region] nr Kalkrand, 10 May 1955, *B. de Winter 3497* (K. M); [Kavango West Region] nr Tondoro, 5 Mar 1956, *B. de Winter & W. Marais 4998* (M); [Otjozondjupa Region] Erichsfelde Farm, 13 Mar 1956, *O.H. Volk 11826 & 11828* (BR0000016286863, M); [Hardap Region] Haribes, 9 Apr 1956, *O.H. Volk 12296* (M); [Kunene Region] Kaokoveld, 27 Mar 1957, *B. de Winter & O.A*. *Leistner 5192* (K, M); [Kunene Region] nr Etanga, 7 Apr 1957, *B. de Winter & O.A. Leistner 5436* (K, M); [Erongo Region] Karibib, 1150 m a.s.l., 17 May 1957, *R. Seydel 1143* (Z-000033456); [Hardap Region] Bergland, 1575 m a.s.l., 10 May 1961, *R. Seydel 2858* (L1673883, M, WAG1404405); Windhoek distr., Farm Voigtland, 10 Mar 1963, *H. Leippert 4351* (FT); Windhoek, 12 Mar 1963, *R. Seydel 3424* (BR0000016286870, K, M); [Kunene Region] Etosha Pan, 8 miles N of Namutoni, 18 May 1963, *B. Nordenstam 2688* (M); [Hardap Region] Maltahöhe, 1969, *O.H. Volk 6421* (M); [Ohangwena Region] Oshandi, 19 Mar 1973, *R.J. Rodin 9110* (K, M); [Otjozondjupa Region] Okahandja, 28 Apr 1976, *G. Woortman 2116* (K, M); [Kunene Region] N of Outjo, 12 Mar 1997, *G. Germishuizen 9710* (WAG1404427);

**Niger** (new records): [Tilaberi Region] Toukounous vill., Jul–Aug 1967, *B. Bartha 31-10* (K, M, MSB122467); [Agadez Region] 30 km NW of Agadez town, 520 m a.s.l., 19 Sep 1977, *P. van der Veken 12727* (BR0000013834067); NP of West Niger, Mékrou, 2 Dec 1986, *E. Robbrecht & N. Leman 3467* (BR0000016286474); Niamey, 28 Sep 1987, *N. Leman 181* (BR0000016286467); [Agadez Region] nr Sekiret, 24 Sep 1988, *E. Schulz s.n.* (GZU000274163); Nianey Prov., 22 km N of Niamey, 20 Sep 1989, *K. Küppers 9* (FR-0010167);

**Nigeria** (new records): Sokoto [State], May 1921, *B. Moiser 14* (K); [Bauchi State] Nabardo, 21 Sep 1921, *H.V. Lely 620* (K); [Taraba State] Gembu, 1620 m a.s.l., 29 Jan 1958, *F.N. Hepper 1815* (K); [Benue State] Buruku vill., 9 Oct 1961, *J. Olorunfemi 55048* (WAG1404188); [Benue State] Buruku vill., 9 Oct 1964, *J. Olorunfemi 55048* (K); [Kano State] Gaya, Sep 1979, *Sharland 658* (K); Borno State, nr Gajiganna, 4 Sep 1992, *K. Neumann 1280* (FR-0010175); Borno State, Maiduguri to Monguno, 2 Oct 1994, *B. Zach 20* (FR-0010161); Borno State, Lake Tilla, 22 Oct 1994, *U. Salzmann 58* (FR-0010177);

**Réunion** (French Overseas Department; new record): Dos d’Ane, Mar 1974, *F. Friedmann 2294* (K);

**Rwanda** (new records): [Eastern Prov.] nr Biharagu, 1400 m a.s.l., 12 Jun 1958, *G. Troupin 7900* (K); [nr Gisenyi town] Rubona [Peninsula], 1700 m a.s.l., 12 Sep 1958, *G. Michel 5637* (BR0000013708306, K); Kibungo Prov., Akagera NP, 19 May 1969, *G. Bouxin & M. Radoux 442* (BR0000013708221, FT); [Southern Prov.] Muhura, 26 Jun 1972, *L. van Puyvelde 29* (BR0000013708504); [Southern Prov.] Butare town, 19 Jul 1974, *P. Auquier 3268* (WAG1404394); [Western Prov.] Bugarama, 1000 m a.s.l., 13 Jun 1975, *B. Runyinya 99* (BR0000013837891);

**Senegal** (new records; selected): Kaolack town, 1950–1951, *R.P. Berhaut 490* (BR0000016286405); [Louga Region] nr Dahra, 17 Sep 1961, *J.A. Raynal 7582* (BR0000016272811); [Casamance Region] Kabrousse, 4 Nov 1978, *C. Vanden Berghen 2969* (BR0000016286382); [Ziguinchor Region] Badiouré, 16 Nov 1983, *C. Vanden Berghen 6111* (BR0000013833893); [Basse-Casamance Region] Badiouré, 18 Nov 1983, *C. Vanden Berghen 6183* (BR0000013833909); Guires Lake, 12 Nov 1984, *P. Bamps 7623* (BR0000016286368); [Casamance Region] Abéné vill., 2 Nov 1987, *C. Vanden Berghen 8039* (BR0000016286399); Vindou-Tingoli area, 20 m a.s.l, Sep 1989, *J.E. Lawesson 5367* (K); [Basse-Casamance Region] Fegroum, 8 Nov 1990, *C. Vanden Berghen 9293* (BR0000013833916, WAG1404191); Sine Saloum Prov., Delta du Saloum NP, Sep 1991, *A.M. Lykke & al. 503* (K); [Basse-Casamance Region] Abéné, 18 Oct 1995, *C. Vanden Berghen 10103* (BR00000162286436); Bandia Reserve, 25 Sep 1996, *A. Druart 29* (BR0000016286429); Diaoulé vill., 19 Sep 2005, *E. Faye 54* (BR0000016286375);

**Sierra Leone**: see type of f.annulosasubf.angustifolia; Loma Mts., 29 Dec 1963, *J.K. Morton 357* (WAG14042000); [Southern Prov.] Njala, 19 Jan 1964, *J.K. Morton 654* (WAG1404202); Tingi Mts., 15 Dec 1965, *J.K. Morton & D. Gledhill 3122* (K);

**Somalia** (new record): [Lower Jubba Region] 27 km of Badadda [Bad Adda] town, Ola Uager, 28 Aug 1959, *G. Moggi & R. Ravazzano 1722* (K, MA-01-00516272);

**South Africa** (new records): [Eastern Cape Prov.] Uitenhage, 1847, *R.C. Alexander s.n*. (BM); [KwaZulu-Natal Prov.] Karkloof, 1875–1880, *A. Rehmann 7405* (Z-000033307); [Mpumalanga Prov.] Barberton, 2800 ft, 1890, *E.E. Galpin 920* (K); [Eastern Cape Prov.] Komgha, Mar 1891, *H.G. Flanagan 733* (BM); [Western Cape Prov.] Wynberg, 9 May 1892, *B. Schlechter 735* (BR0000013835309, FI, WU); Grahamstown [Makhanda] town, May 1893, *B. Schlechter 2634* (BM, BR0000013835385, K, WU); nr Capetown, 1895, *H. Bolus 2913* (K); nr Pretoria, 24 Mar 1906, *R. Leendertz 799* (K); [Eastern Cape Prov.] Centane, Mar 1911, *A. Pegler 1499* (BM, K); [Western Cape Prov.] Knysna, Apr 1912, *W.G. Worsdell s.n*. (K); [Limpopo Prov.] Tzaneen, 30 Oct 1913, *A.O.D. Inogg 9548* (K); Western Cape, Kirstenbosch, Apr 1917, *M.M. Page s.n.* (BOL0222316); [Eastern Cape Prov.] Krom River, Mar 1922, *H.G. Fourcade 2136* (BOL73890); Johannesburg, 9 Mar 1924, *C.E. Moss 9302* (BM); [Western Cape Prov.] Wynberg Hill, 2 Jun 1931, *T.M. Salter 795* (BM, K); [KwaZulu-Natal Prov.] Durban, Sep 1933, *A. Meebold 13054* (M); Pretoria, 5 Apr 1934, *A.O.D. Mogg 14934* (BR0000013708764); KwaZulu-Natal Prov., Ixopo, Mar 1935, *N.M. Otto 19A* (M); KwaZulu-Natal Prov., Durban, Apr 1936, *H.M.L. Forbes 1281* (BOL0291980); [Northern Cape Prov.] Rietfontein, 25 Nov 1939, *anonymous s.n.* (M); [Gauteng Prov.] Baviaanspoort, 15 Apr 1946, *W. Giess 15828* (M); [Eastern Cape Prov.] Stutterheim, 11 Dec 1948, *A.P.H. Acocks 9461* (K); Rehoboth distr., 19 Apr 1949, *R.G. Strey 2534* (BOL0291970); [Western Cape Prov.] Claremont, Feb 1950, *Pillans 10034* (BR0000013835378); [Limpopo Prov.] Letaba [Municipality], 18 Aug 1958, *G.C. Scheepers 164* (BM, M); nr Pretoria, 11 Apr 1962, *L. Bernardi 9058* (LE); KwaZulu-Natal Prov., Oribi Gorge, 10 Oct 1963, *J. Lanjouw & B. Lanjouw-Raven 450* (U1052435); Free State Prov., Farm Mequatlingsnek, 1600 m a.s.l. Apr 1972, *M. Jacobs 8530* (K, L1695059); Cape Town, 2 Oct 1972, *E. Esterhuizen s.n*. (BOL0222314); Transvaal Prov., Nylsvley NR, 27 Mar 1976, *A.J.M. Leeuwenberg 11003* (BR0000016286894); [Eastern Cape Prov.] Grahamstown [Makhanda town], 1 Mar 1978, *R.D.A. Bayliss 8584* (M); KwaZulu-Natal Prov., Nkandla Forest, 4 Feb 1982, *J. Lambinon & M. Reekmans 257* (BR0000016286917);

**South Sudan** (new record): [Upper Nile State] Boing, 11 Oct 1951, *M.I. Sherif 2886* (K);

**Sudan** (new records): Erkowit [Ar Kaweit], 3600 ft, [without date] *anonymous 24* (K); [White Nile State] Mt Arashkol, 9 Oct 1839, *Kotschy 148* (BR0000013835835, BR0000016286528, HBG503187, M0241531, M0241532, WAG1404426, WU); Central Darfur, Zalingei, Oct 1921, *H. Lynes s.n*. (K); Erkowit [Ar Kaweit], 1928, *Maffey 7* (K); [Al Qadarif State] Al Gadaref distr., 5 Sep 1951, *B. Beohir 314* (K); North Kordofan State, Bara distr., 23 Sep 1962, *G.E. Wickens 519* (K); Central Darfur, 120 km E of Zalingei, Jebel Marrah Mts., 1650 m a.s.l., 20 Jan 1965, *W.J.J.O de Wilde & al. 5437* (BR0000013834296);

**Tanzania** (new records; selected): [Mwanza Region] Mwanza, 1935, *R.H. Davis 33* (K); Shinyanga, 3800 ft, 29 May 1952, *J.R. Welch 178* (K); Malagarasi [River], 5 Aug 1952, *G. Michel 3601* (BR0000013835828); [Mara Region] Ikoma, 30 Feb 1953, *R. Tanner 1849* (K); [Shinyanga Region] Shinyanga town, 3500 ft, 29 May 1953, *J.R. Welch 178* (M); [Ruvuma Region] Songea distr., 1956, *E. Milne-Redhead & P. Taylor 9621* (BR0000016286641, K); between Lembeni & Same, 3000 ft, Jan 1957, *P.R.O. Bally s.n*. (K); [Kagera Region] Kirushya vill., 5500 ft, 5 Apr 1960, *R.E.S. Tanner 4827* (BR0000013834760, WAG1404267); Mbeya distr., Igawa, 4000 ft, 1 Apr 1962, *R. Polhill & S. Paulo 1964* (BR0000013835002, K); [Rift Valley Region] Lake Manyara NP, 3 Apr 1962, *H. Dingle 191* (K); [Kilimanjaro Region] Kilimanjaro Mt., 1524 m a.s.l., 25 Feb 1968, *R. Ravazzano 5* (FT); [Iringa Region] Mdonya River, 24 Apr 1970, *P.J. Greenway & K. Kanuri 14414* (M); [Dodoma Region] Mtera, 600 m a.s.l., 5 May 1971, *B. Mhoro 1106* (K, WAG1404264); Dodoma Region, Ikowa Reservoire, 20 May 1975, *I. Backéus 1311* (BR0000013834982, K); [Tanga Region] Lushoto, 17 Mar 1979, *J. Grabner 244* (W0333295); [Singida Region] Mgela vill., 1340 m a.s.l., 14 May 1983, *Kisena & Ruffo 86* (K, WAG1404206); Tanga Region, Lushoto distr., West Usambara Mts., Mazumbai Field Station, 1520 m a.s.l., 17 Jan 1985, *S.T. Iversen 85013* (K); Iringa Region, Iringa distr., Ruaha NP, 850 m a.s.l., 17 May 1987, *J. Lovett & al. 2166* (K); Iringa Region, Mufindi distr., 1830 m a.s.l., 20 May 1987, *J. Lovett 2188* (K); Tanga Region, Handeni distr., Gendagenda Forest, 7 Sep 1991, *anonymous s.n.* (K); Kagera, Bakuba Rural distr., W of Minziro vill., 1130 m a.s.l., 20 Mar 2001, *L. Festo & J. Francis 1061* (K); Arusha Region, nr Longido township, 1430 m a.s.l., 15 Jun 2012, *C.J. Kajombo & C. Makyao 7686* (WAG1404203); Manyara Region, Mt. Hanang area, nr Gidewari vill., -4.357316, 35.373525, 14 Jun 2022, *A.P. Sukhorukov s.n*. (MW, W);

**Togo** (new records): [without exact location and date] *J.F. Brunel 6708* (BR0000013834029); [Savanes Region] Mandouri, 19 Nov 1983, *anonymous 7875* (TOGO01604);

**Uganda**: see type of A.asperaf.annulosa; Morungole Mt., 7500 ft, 11 Nov 1939, *A.S. Thomas 3261* (K); Moroto town, 4600 ft, Sep 1956, *Wilson 256* (K); West Buganda distr., Mpanga Forest, 2 Sep 1974, *A.B. Katende 2317* (K);

**Zambia** (new records; selected): [Central Prov.] Mumbwa, 1911, *Macaulay 687* (K) together with *A.porphyrostachya*; [Southern Prov.] Mazabuka [town], 1934, *J.D. Martin 603* (BR0000013708740, K); [Copperbelt Prov.] Mufulira, 23 May 1948, *A.W. Cruse 350* (K); [Southern Prov.] Mapanza, 3300 ft, 4 Apr 1953, *E.A. Robinson 154* (K); Lusaka, 4000 ft, 22 May 1955, *E.B. Best 101* (BR0000013835132, K); [Southern Prov.] Mazabuka [town], 28 Mar 1963, *H.J. van Rensburg 1848* (BR0000013835163); [Copperbelt Prov.] Kitwe, 16 Apr 1963, *D.B. Fanshawe 7772* (K); [Luapula Prov.] Chibembe River, 2000 ft, 22 Mar 1967, *S.D. Prince 410* (K); [Northern Prov.] nr Mbala, 25 Apr 1968, *M. Richards 23249* (K); Luangwa Valley, Apr 1971, *N.O.G. Abel 247* (BR0000016286764); [Northern Prov.] Mbala, 29 May 1978, *M. Sanane* 1199 (BR0000013835156); [Central Prov.] Kabwe town, 1250 m a.s.l., 21 Apr 1998, *B. Leteinturier & al. 156* (BR0000013835118);

**Zimbabwe** (new records; selected): Mazowe distr., Mar 1906, *F. Eyles 298* (BM); [Manicaland Prov.] Chirinda, 22 May 1906, *C.F.M. Swinnerton 512* (K); Salisbury [Harare], 17 Jul 1913, *anonymous s.n.* (K); [Manucaland Prov.] Umtali [Mutare], Odrani River valley, 1914, *A.G. Teague 81* (BOL0291973); [Matabeleland South Prov.] Matopos [Matobo] distr., 14 Apr 1931, *anonymous 3965* (BM); [Manicaland Prov.] nr Nuza Mt., 3850 ft, 15 Jun 1934, *H.B. Gilliland 344* (BM); Bvumba [Mts.], 5300 ft, 21 Apr 1950, *N.C. Chase 2151* (BM); [nr Bulawayo town] Matobo, 4000 ft, May 1959, *O.B. Miller 5934* (M); [Midlands Prov.] 18°29'S, 29°00'E, 17 Feb 1967, *M.C. Cole 246* (K); [Masvingo Prov.] Nuanetsi, 1500 ft, 20 Jan 1972, *J.I. Barnes 146* (K); [Matabeleland South Prov.] Matobo distr., 14 Apr 1973, *G. Norrgrann 326* (BR0000016286856); [Masvingo Prov.] Makoholi Exp. Station, 13 Sep 1978, *E. Senderayi 205* (BR0000016286825); [Manicaland Prov.] Nyanga, 1650 m a.s.l., 6 Mar 1981, *D. Philcox & al. 8871* (BR0000016286832, K); [Matabeleland North Prov.] Hwange distr., 14 Apr 1999, *C. Wengler 165* (BR0000013835217).

**Arabia**

**Oman** (new records): [Planta culta] Cultivated voucher originated from Dhofar Governorate, Ayn Rizat Spring, *H.D.V. Prendergast s.n*. (K); Dhofar, Salalah, 21 Sep 2001, *M. Raffaelli & al. 43* (FT); Dhofar Region, between Salalah & Mirbat Pista, 11 Sep 2002, *M. Raffaelli & al. 1023* (FT); Dhofar Region, Salalah to Mirbat, 600 m a.s.l., 22 Sep 2002, *M. Raffaelli & al. 269* (FT);

**Saudi Arabia** (new records): Wadi Kharar, nr the foot of the Taif escarpment granite, 1500 ft, 10 Feb 1980, *I.S. Collenette 1768* (K); [Mecca Prov.] 30 km SW of Taif town, 1830–2050 m a.s.l., 30 Jan 1988, *A.A. Fayed 1343* (M); Al-Baha Region, SE of Baljurashi, 7 Apr 1988, *A.A. Fayed 1394* (M);

**Yemen** (new records): Socotra Island, Aug 1880, *B. Balfour 39* (BM, K, LE); Socotra, 1897, *Th. Bent s.n.* (K); North Yemen, Jebel al Abid, 27 Aug 1937, *C. Rathjens 104* (BM); 14 km NW of Sana’a, 26 Sep 1981, *D. Podlech 35827* (MSB122469).

***Achyranthesaspera* L.**

**Africa**

**Benin**: Zou Dept., Dassa, 4 Nov 1998, *V. Adjakidjè & al. 2197* (BR0000016286450, K, W0333254, WAG1404336);

**Cameroon**: Southwest Region, Kupe vill., 24 Jan 1995, *M. Cheek & al. 7151* (WAG0075654);

**Chad**: Hadjer-Lamis Region, Djimlilo vill., 13 Dec 1969, *F.N. Hepper 4184* (BR0000013834210, K);

**Comoros**: Moroni, 30 Jul 1981, *H. Doutrelepont 1049* (BR0000013709099);

**DR Congo**: [Orientale Prov.] Nioka, 27 Jan 1946, *A.S. Taton 18* (K); [South Kivu Prov.] Uvira town, 780 m a.s.l., Aug 1951, *Marlier 1327* (WAG1404151); Virunga NP, Lulimbi Camp, 19 Dec 1981, *J. Van Gisel s.n*. (BR000000905112);

**Egypt**: [without location and date; old specimen from 19^th^ century] *Fischer s.n*. (M) [Not shown on map due to unknown location];

**Europa Island** (French Southern and Antarctic Islands): Territory of a military camp, 10 Apr 2015, *J. Hivert & al. 1195* (P00956454);

**Ethiopia**: Omo-Bottego River, 11 Aug 1939, *R. Corradi 8594* (FT); [Afar Region] Amibara [distr.] Melka Sede, 1968–1969, *N. Longhitano s.n*. (FT, K);

**Ghana**: Legon [Accra], May 1985, *D.K. Abbiw 47250* (K, US03544302);

**Kenya**: nr Mombasa, Nov 1909, *E.A. Mearns s.n*. (BM); Kisumu, Feb 1915, *R.A. Dümmer s.n*. (BM, K); Mombasa town, 25 Aug 1981, *N. Tzvelev 424* (LE); Mombasa to Kilifi, road to Kaloleni vill., 16 Dec 1969, *J.M. Reitsma 440* (BR0000013834579, WAG1404166); Tana River distr., nr Tana River, 3 Mar 1977, *S.S. Hooper & C.C. Townsend 1163* (K); [Laikipia County] 18 km NE of Nanyuki town, 22 Oct 1977, *M.G. Gilbert 4881* (WAG1404402); Kilifi County, Kilifi, 23 Nov 1981, *V. de Meester-Manger Cats 210* (WAG1404209); Msambweni, 20 m a.s.l., 5 Aug 1982, *S.A. Robertson 3333* (K); [Nandi County] Kaimosi vill., 2 Nov 1984, *A. Hohl 106* (W0333211); Tana River distr., Tana Delta, 6 Aug 2010, *C. Leauthaud & al. 64* (K);

**Liberia**: [Maryland County] Cape Palmas, 17 Jun 1970, *J.G. Adam 25987* (P00695158);

**Madagascar**: Majunga [Mahajanga], 23 Apr 1912, *K. Afzelius s.n*. (K); [Androy Region] Ambovombe, 1931, *R. Decary s.n.* (BM); [Androy Region] Ambovombe, 9 Aug 1931, R. *Decary s.n.* (K); Nosy Be Island, 28 Jun 1983, *J. Frazier 661* (K); Ambahatra, 13 Jun 1998, *L. Gautier & al. 3352* (K); [Atsimo-Andrefana Region] Betioky to Ampanihy, 25 Aug 2003, *M. Andriamahay 603* (K); Menabe Region, Bekonazy, 5 Jun 2015, *N. Rakotonirina & al. 1079* (P01048115);

**Mauritius** (selected): [without exact location and date, early 19^th^ century] *Hilsenberg s.n*. (BR0000013835422); [without exact location] 1819–1821, *F. Cohaut s.n.* (BM); 1879, [without exact location] *Sieber 146* (K, M0241516); Rose Hill, Jan 1953, *E. Rochecouste 3* (MAU0017605); Rodrigues Island, Jul 1970, *Th. Cadet 2754* (K); Albion, 18 Feb 1972, *J. Guého s.n.* (K, MAU0017611); Rodrigues Island, 1 May 1982, *J. Guého 20374* (MAU0017618); Gunners Quoin, Jul 1982, *D. Bullock & S. North 20508* (MAU0017612); Round Island, 29 Jul 2003, *M.C. Johansson 23547* (MAU0017613); Flat Island, 16 Apr 2005, *K. Pynee & J.C. Sevathian 24534* (MAU0017615);

**Mayotte** (French Overseas Department): Dzoumogne, [without date] *anonymous 4* (P00290533); [without exat location] 1855, *Boivin s.n*. (FI-WEBB154851); Bandrele, 29 Nov 2000, *J.-N. Labat & F. Barthelat 3320* (K);

**Mozambique**: [Nampula Prov.] Zambesi River, Boroma, 1892, *Menyharth 1197* (WU); [Sofala Prov.] Nova Chupanga [probably Chemba distr.] 22 Jul 1933, *Lawrence 55* (K); Beira town, Nov 1939, *A. Scarpa s.n*. (FT); [Maputo] Villa Luisa, 17 May 1965, *G. Caldeira & A. Marques 405* (WAG1404239); Tete distr., Tete–Songo road, 7 Apr 1972, *J.M. de Aguiar Macodo 5257* (K); Nampula, 20 Jul 1979, *S. Macitela 25* (WAG1404231); Maputo, 26 Dec 1983, *D. Zunguse & al. 481* (BM);

**Namibia**: Windhoek Farm Finkenstein, 11 April 1966, *R. Seydel 4412* (LE);

**Réunion** (French Overseas Department): [without exact location, date and collector] *3* (MAU0017616); Saint-Leu, 3 Apr 2019, *E. Bidault & al. 4573* (P01085572);

**Rwanda**: nr Kigali, 1450 m a.s.l., 13 Oct 1974, *P. Auquier 4560* (WAG1404298);

**São Tomé and Príncipe**: São Tomé Island, Aug 1885, *A.F. Moller 684* (BR0000013833985);

**Senegal**: Dakar, Nov 1925, *L. Hauman s.n*. (BR0000013833879); Dakar, Oct 1950, *R.P. Berhaut 2093* (BR0000016286412);

**Seychelles**: Christmas Island, [without year] *J. Jackson s.n*. (BM); [without exact location] Sep 1871, *J. Horne 338* (K); Denis Island, 10 Mar 1962, *C. Jeffrey 1210* (K); Astove Atoll, 5 Mar 1968, *F.R. Fosberg 49707* (K); La Digue Island, 22 Dec 2005, *V. Bochkin & N. Bokal s.n*. (MHA);

**Somalia**: [Middle Juba Region] Bidi, 30 Jun 1913, *G. Paoli 448* (FT); [Bay Region] Burhakaba [town], 21 Sep 1929, *L. Senni 856* (FT); [Lower Shabelle Region] Merca town, 17 Nov 1935, *F. Bigi 108* (FT); [Lower Shabelle Region] South Qoryoley distr., Beynax Barre farm, 14 Jul 1989, *A.M.I. Barkhadle 276* (FT);

**South Africa**: [KwaZulu-Natal Prov.] nr Durban, Sep 1933, *A. Meebold 13051* (M);

**South Sudan**: [Central Equatoria state] Bahl al-Jabal, Terekeka [community], 7 Jul 1929, *N.D. Simpson 7267* (K); [Lakes Region] Shambe, 14 Jul 1929, *N.D. Simpson 7376* (BM);

**Tanzania** (selected): [Zanzibar] Pemba Island, [without date] *G.H. Vaughan 655* (K); Zanzibar, Sep 1873, *J.M. Hildebrandt 1150* (LE – right-hand specimen, M); [Tanga Region] Pangani, Jun 1890, *F. Stuhlmann 105* (M); [Tanga Region] Mashewa, 400 m a.s.l, Sep 1915, *A. Peter 13869* (B 10 0153181); [Mwanza Region] Mwanza town, 21 Oct 1925, *R.L. Davis 60* (K); [Lindi Region] Nachingwea, 1500 ft, 19 Apr 1955, *B. Anderson 1051* (BR0000013834777, K); Tanga Region, Boza, 3 Jul 1956, *R.E.S. Tanner 2972* (BR0000013834838, K); [Mara Region] Musoma town, 1 May 1959, *R.E.S. Tanner 4225* (K); Zanzibar [Unguja Island], Chukwani, 9 Apr 1961, *H.G. Faulkner 2805* (BR0000013835859, K); [Tanga Region] Korogwe town, 25 Nov 1962, *M.E. Archbold 14* (K); Tanga distr., Sawa, 14 May 1965, *H.G. Faulkner 3521* (BR0000013835071); [Kagera Region] Karagwe distr., Segora forest, 9 Feb 1968, *H. Faulkner 4073* (WAG1404277); Dodoma Region, Ikowa Dam, 900 m a.s.l., 29 Jul 1970, *M. Thulin & B. Mhoro 519* (K); Dar es Salaam, Oyster Bay, 11 Aug 1974, *J. Frazier 1067* (K); Tanga Region, Pangani town, 8 Jan 1993, *Limbach & Mshana 204* (M); [Tanga Region] Tanga town, 1 Sep 1995, *Waziri 152* (M); Zanzibar, Unguja Island, 22 Jun 2022, *A.P. Sukhorukov s.n*. (MW);

**Uganda**: West Nile distr., 10 Aug 1953, *R. Chancellor* 155 (K, M); 20 km W of Pakwach, 5 Oct 1974, *A.B. Katende 2338* (K);

**Zimbabwe**: [Masvingo Prov.?] Sabi, 800 ft, 14 Jun 1950, *H. Wild* 28079 (BM, M).

**Arabia**

**Oman**: Muscat, [without date] *Aucher-Eloy 9294* (P04559070); Bohar Farm, 20 Feb 1973, *C. Parker 158* (BM); Wadi Sahtan, 1400 m a.s.l., 4 Apr 1975, *J.P. Mandaville 6227* (BM); Dhofar, Salalah, 26 Sep 2015, *P. Escobar Garcia 1073* (W);

**Saudi Arabia**: see type of Achyranthesargenteavar.obovata; Fatme [probably Wadi Fatimah near Jeddah], [without date, 19^th^ century] *S. Fischer 95* (BR0000013457310);

**United Arab Emirates**: Emirate of Fujairah, Al Badiyah, 15 May 2020, *V.V. Byalt & M.V. Korshunov 2978* (LE01194673);

**Yemen**: Lahadj [Lahidj city], 26 Dec 1889, A. Deflers 180 (P04559065); Socotra Island, 21 Feb 1953, *G. Popov 99* (BM); Aden, Shaikh Uthman, Jan 1963, *J. Waring 185* (BM); Lahij Gov., Lahij town, 20 Mar 1988, *L. Boulos 17386* (BM).

**Achyranthesmauritiana Moq**. – see main text.

***Achyranthesporphyrostachya* Moq.**

**Africa**

**Angola**: Luanda, [without date, probably mid-19^th^ century] *F. Welwitsch 6530* (BM, BR0000013835101, K); [Namibe Prov.] Bero River, Aug 1859, *F. Welwitsch 6499* (BM, K); Benguela, Jul 1891, *Menyharth 12* (WU); Near the Kunere River between Kiteve and Humbe, 3 Jun 1900, *H. Baum 961* (BM – left-hand specimens, K0002437271, M); nr Cunene Revier, Ruacaná, 9 Jun 1937, *A.W. Exell & F.A. Mendonça 2798* (BM); Huila Prov., Lubango, 29 May 1974, *A. Borges & al. 409* (K);

**Benin**: without exact location and date, *G. le Testu 189* (BR0000013834050, left-handed specimen);

**Botswana**: N’Garni, 13 May 1930, *Vernay-Lang Kalahari Expedition 28743* (BM, M); [Central district] nr Mopipi, 850 m a.s.l., 17 Apr 1973, *G. Glanville 537* (BR0000013835286, K, M); Kwara floodplain, 1 May 1973, *P.A. Smith 561* (K); Gaborone, 3250 ft, 12 Feb 1974, *P.J. Mott 153* (K); Kwando, 1000 m a.s.l., 19 Apr 1977, *D.T. Williamson 127* (K); Ngamiland distr., N of Maun, 920 m a.s.l., 28 Mar 1987, *D.G. Long & D.A.H. Rae 444* (K);

**Burundi** (selected): [Bururi Prov.] nr Kitete [Gatete] vill., Nov 1922, *Elskens 137* (BM); Bujumbura, 850 m a.s.l., 28 Sep 1966, *J. Lewalle 1040* (BR0000013837181); Bujumbura, Sep 1967, *J. Lewalle 1040* (K, WAG1404281); [Bubanza Prov.] Rukoko, 20 Feb 1980, *J. Bouharmont 12955* (BR0000013837143); [Rumonge Prov.] Gitaza, 780 m a.s.l., 5 Aug 1980, *P. Ndabaneze 980* (BR0000013837228); Bujumbura, 5 May 1988, *J. Saintenoy-Simon 8* (BR0000013835781);

**Cameroon** (first records): [Far North Prov.] Waza Wild Life Reserve, 22 Jan 1966, *A.J.M. Leeuwenberg 7484* (K, WAG1404136); West Region, Baranka, 27 Apr 2005, *B. Tchiengue & al. 2147* (K);

**Cape Verde**: São Vicente Island, Monte Verde, *herb. Hookerianum 65* (K001134168);

**Chad**: [Hajer-Lamis Region] Djimtilo, 13 Dec 1969, *F.N. Hepper 4184* (BR0000013834210);

**Djubouti**: French Somalia, [Tajourah Region] Randa, 1000 m a.s.l., 29 Sep 1956, *E. Chedeville 1655* (FT);

**DR Congo**: [South Kivu Prov.] Luvungi, Dec 1907, *P. Aguzzi 56* (BR0000016287327); [South Kivu Prov.] Kabare [Territory] 25 Aug 1914, *J. Bequaert 5447* (BR0000016287389); Albert [Mwitanzige] Lake, Aug 1935, *H. Bredo 1505* (BR0000016287204); [Ituru Prov.] Kasenyi, Nov 1935, *H. Brédo 1793* (BR0000016287228); Katanda [Territory], Sep 1937, *J. Lebrun 7617* (K); [North Kivu Prov.] Ruindi, Oct 1937, *J. Lebrun 7997* (K); [Maniema Prov.] Kalulu vill., May 1939, *H. Brédo 2874* (BR0000013836740); [Lualaba Prov.] Kasumbi, Apr 1940, *P. Quarré 6297* (BR0000013837334); Virunga NP, Lake Edward, 19 Jun 1945, *R. Germain 3937* (BR0000013836375); Ruzizi Plain, Jan 1950, *R. Germain 5558* (BR0000013836382); [Haut-Katanga Prov.] Mitwaba [Territory], 17 Jun 1955, *J. Brynaert 395* (BR0000013708948); [South Kivu Prov.] Uvira town, 27 Aug 1955, *J.-J. Symoens 1340* (BR0000013836634); [South Kivu Prov.] Kalonge, 1060 m a.s.l., Oct 1956, *G.F. de Witte 13638* (K); [Maniema Prov.] Kalinga, 1050 m a.s.l., Nov 1956, *G.F. de Witte 13931* (K); nr Lake Albert, 5 Nov 1956, *G.F. de Witte 13790* (BR0000013836245); North Kivu Prov., Albert [Virunga] NP, Nov 1956, *G.F. de Witte 13901* (K); South Kivu Prov., Kalehe Territory, Kifunga, 1000 m a.s.l., Jan 1957, *G.F. de Witte 13990* (K); North Kivu Prov., Rutshuru town, Feb 1957, *G.F. de Witte 14173* (K); [South Kivu Prov.] Kahuzi, 20 Jul 1976, *L. Pauwels 5641* (BR0000013836580); [North Kivu Prov.] Beni Zone, Ishango, 28 Nov 1979, *H. Breyne 3859* (BR0000013835750);

**Egypt**: Aswan, nr Nile River, Jan 1904, *R. Muschler s.n*. (K);

**Eritrea** (selected): [Debub Region] Akrur, 1900 m a.s.l., 10 Mar 1892, *G. Schweintfurth & D. Riva 1025* (K); [Northern Red Sea Region] Nakfa town, 19 May 1892, *A. Terracciano & A. Pappi 913* (FT); [Debub Region] Lebca, 22 May 1892, *A. Terracciano & A. Pappi 1036* (FT); [Southern Rea Sea Region] Adarte vill., 26 Jan 1893, *A. Terracciano & A. Pappi 2762* (FT); [Northern Red Sea region] Agametta vill., 19 Mar 1893, *A. Pappi 3127* (FT); [Debub Region] Sala Dharo, 2500 m a.s.l., 7 Oct 1902, *A. Pappi 2310* (FT); nr Dwarba [town], 1900 m a.s.l., 8 Oct 1902, *A. Pappi* 504 (FT); [Anseba Region] Garassi, 13 Oct 1905, *A. Pappi 6508* (FT); [Gash-Barka Region] Algata, 450 m a.s.l., 31 Mar 1909, *A. Fiori 955* (FI); Habab, Oazat, 21 Apr 1909, *A. Pappi* 8388 (FT);

**Eswatini**: nr Mankaiana, 19 Mar 1959, *R.H. Compton 28663* (BM); Lubombo Region, 27 Apr 2024, *L. Loffler* (image available at: https://www.inaturalist.org/photos/375635369);

**Ethiopia** (selected): see type of A.asperavar.robusta; Amhara Region, Dembia, 8 Sep 1909, *Chiovenda 1951* (FT); [Rift Valley Prov.] Zuai Lake, 24 May 1937, *L. Senni 615* (FT); [Southern Nations, Nationalities, and Peoples Region] Bonga, Nov 1937, *D. Saccardo s.n*. (FT); [Oromia Region] Bishoftu [Debre Zeit] town, 1938, *P. Benedetto 57* (FT); Omo River basin, Rive del Caschei, 3 Jul 1939, *R. Conradi 3223* (K); [Oromia Region] Bishoftu town, 6000 ft, Oct 1951, *C. Curli 164* (BM); Idli valley, 45 km ESE of Harar, 1550 m a.s.l., 26 Jul 1961, *W. Burger & A. Getahun 371* (K); Shoa Prov., 17 km S of Addis Ababa, 6830 ft, 24 Nov 1961, *F.G. Meyer 7517* (K); [Oromia Region] 4 km S of Alem Tena town, 1720 m a.s.l., 13 Jul 1963, *E. Beals 262* (K); 8 km N of Addis Ababa, 2800 m a.s.l., 11 Oct 1965, *W.J.J.O de Wilde & B.E.E. de Wilde-Duyfjes 8217* (BR0000013834364); [Afar Region] 50 km W of Awash station, 800 m a.s.l., 8 Apr 1966, *W.J.J.O de Wilde & B.E.E. de Wilde-Duyfjes 10663* (BR0000013834388, WAG1404312); Harari Region, 20 km SE of Harar town, 5 Nov 1966, *W.J.J.O de Wilde & B.E.E. de Wilde-Duyfjes 9962* (WAG1404313); nr Alemaya, 2030 m a.s.l., 10 Jul 1967, *E. Westphal & M.C. Westphal-Stevels 509* (BR0000013834432); Shao Prov., E of Maki town, 5440 ft, 6 Jun 1968, *D. Hundessa 15* (K); Awash valley, 4 miles W of Metehara, 10 Aug 1968, *P.M. Headley 225* (K); NE shore of Lake Abiyata, 1550 m a.s.l., 15 Apr 1969, *J.J.F.E. de Wilde 4908* (BR0000013834425, WAG1404315); Extreme South West, Omo River valley, 1300 ft, 1 Jul 1969, *C.J. Carr 640* (BR0000013834326, K); [Harari Region] Fafan River, 1590 m a.s.l., 7 Jun 1972, *C.J.P. Seegeler 2305* (BR0000013837631, WAG1404320); [Oromia Region] Jimma, 20 Aug 1972, *C.J.P. Seegeler 2397* (BR0000013837648, WAG1404317); [Somali Region] 70 km from Dire Dawa town, 18 Oct 1972, *C.J.P. Seegeler 2700* (WAG1404316); [Oromia Region] Zwai Lake, Feb 1973, *O. Polunin 11594* (K); [Oromia Region] Debre Zeit town, 1800 m a.s.l., 6 Oct 1976, *L. Boulos 9725* (WAG1404318); [Somali Rergion] Sheekh distr., Bokh Reserve, 20 Nov 1979, *C.J. Hansen & al. 6456* (K); Omo NP, 3 Aug 2007, *C. Godefroid 83* (BR000001384340);

**Kenya** (selected): [Makueni County] Kibwezi town, 9 Sep 1906, *G. Scheffler s.n*. (AMD24172, BM, K, WAG1404333); Nairobi, 1915, *W.J. Dowson 288* (K); Nairobi distr., 14 Jul 1924, *J. MacDonald 769* (FT); nr Mariakani town, Mombasa, Feb 1930, *Thomas s.n.* (BR0000013834630); [Narok County?] Maasai Nature Reserve, 15 Sep 1947, *P.R.O. Bally 5361* (K); Nairobi, 1700 m a.s.l., 30 May 1949, *R.A. Maas Geesteranus 4857* (BR0000013834623, L1673905); Meru, Feb 1951, *A.D. Hancock 121* (K); [Baringo County] 10 miles E of Eldama Ravine, 5400 ft, 8 Feb 1952, *A. Bogdan 4459* (K); [Marsabit County] Mayale, 3700 ft, 21 Apr 1952, *J.B. Gillett 12868* (FT, K, M); [Baringo County] Hannington [Bogoria] Lake, 19 Jul 1953, *P.R.O. Bally 9036* (K); Narok distr., Narosura, 6200 f, 16 Aug 1961, *Glover & al. 2431* (BR0000013837693); [Narok County] Oltarakwai, 8000 ft, 18 Aug 1961, *Glover & al. 2525* (BR0000013834517, K); Meru Game Reserve, 16 Jun 1963, *S.G. Mattenge 86* (K); [Machakos County] Kutumani, 5300 ft, 23 May 1966, *P. Ndunda 37* (BR0000013834692); Naivasha distr., 6200 ft, 27 Aug 1966, *E. Polhill 192* (K); [Nakuru County] Nakuru Lake, 2200 m a.s.l., Oct 1966, *Duvignaud s.n.* (BR0000013834500); [Makueni County] N of Nunguni, 1890 m a.s.l., 10 Mar 1968, *O.M. Mwangangi 869* (K); Isiolo distr., 14 Apr 1971, *Z.J. Kimani 269* (BR0000013834531, K); [Kilifi County] nr Malindi, 20 Mar 1973, *G.W. Sangai 15585* (M); Nyeri distr., Nyeri–Kiganjo road, 1770 m a.s.l., 1 Jun 1974, *D.B. Faden & al. 673* (K); [Tana River County] Galana Ranch, 18 Jun 1975, *P.R.O. Bally 16885* (BR0000013834586, FT); Kalisia Hills, 6300 ft, 14 Aug 1977, *Ichikawa 745* (K); [Rift Valley Prov.] West Suk [West Pokot County], Sigor, 3000 ft, 30 Jul 1978, *E. Meyerhoff 91* (K); Coast Prov., nr Gotani vill., 30 Nov 1979, J.*M. Reitsma 374* (BR0000013834678); [Baringo County] Marigat [town], 14 Apr 1983, *A. Mather 9* (K);

**Lesotho**: Leribe [Hlotse], 1914, *Dieterlen 986* (BM);

**Malawi**: Ntandamula Mt. 30 May 1903, *W. Busse 2738* (BR0000013834869); Central Region, Lilongwe distr., 1240 m a.s.l., 21 Mar 1970, *R.K. Brummitt 9250* (K); Central Region, Mchinji distr., nr Namitete, 1130 m a.s.l., 30 Mar 1970, *R.K. Brummitt 8430* (K); Southern Region, nr Lake Chilwa, 640 m a.s.l., 1 Jun 1970, *R.K. Brummitt & C.H. Williams 11206* (K); Lifidzi Goat Breeding Centre, 18 May 1985, *A.J. Salubeni & al. 4203* (K); [Central Region] Salima distr., 19 May 1985, *I.H. Patel & W. Nachamba 2160* (W0333210); Lake Chilwa, 1989, *anonymous 2* (K);

**Mali**: [Mopti Region] Sarredina, 7 May 1952, *J.T. Davey 96* (K);

**Mozambique**: Lourenço Marques Prov., Bombay, Jun 1893, *F. Quintas 150* (BR0000013835248); East coast of Nyassa [Malawi] Lake, 22 Sep 1900, *W.P. Jonson 144* (K); [Gaza Prov.] Guijá, 6 Jun 1947, *J.J. Pedrogão 247* (BR0000013835279);

**Namibia**: Kaokoveld, Otyrtambi, 3 Mar 1885, *W. Belik 43* (WU); Amboland [Ovamboland], Sep 1886, *H. Schinz 7* (Z-000070709); [Otjozondjupo Region] Hamakari, 21 Jan 1939, *O.H. Volk 1182* (M); [Kunene Region] Omuramba, 6 Apr 1953, *H. Walter & E. Walter 240* (M); [Kunene Region] nr Outjo, 4 Apr 1955, *B. de Winter 3039* (K, M); Windhoek Bergland, 1400 m a.s.l., 26 Mar 1963, *R. Seydel 3467* (BR0000013835323); Windhoek Bergland, Finkenstein [Estate], 2000 m a.s.l., 11 Apr 1966, *R. Seydel 4412* (M); [Erongo Region] Otjimbojo, [without date] *H. Kinges 3383* (M);

**Rwanda** (selected): Kigali, 1600 m a.s.l., Feb 1933, *A. Becquet 411* (BR0000013708924); [nr Kigali] Gabiro, 1500 m a.s.l., 4 Feb 1942, *R. Germain 1133* (BR0000013837037); [Western Prov.] Rubona 22 Jul 1956, *G. Michel 4722* (BR0000013837051); [Northern Prov.] Byumba, 1400 m a.s.l., 4 Apr 1957, *G. Troupin 3115* (K); [Eastern Prov.] Kibungo, 1450 m a.s.l., 7 Apr 1958, *G. Troupin 6860* (BR0000013837112, K); Kibungo [Eastern Prov.] 1300 m a.s.l., 17 Sep 1969, *G. Bouxin & M. Rodoux 902* (FT); [Northern Prov.] Shyorongi, 15 Apr 1970, *G. Bouxin & M. Radoux* 1723 (BR0000013837020); [Western Prov.] Bugarama, 10 Aug 1974, *P. Auquier 3627* (BR0000013837006); Kigali, 5 Sep 1974, *J. Lambinon 1093* (BR0000013837884); [Eastern Prov.] Isle Mareba, 1300 m a.s.l., 22 Jul 1978, *G. Troupin 16189* (K); [Northern Prov.] Nyagahanga, 16 May 1982, *M. Aubroeck 5094* (BR0000013836993);

**Senegal**: [without location and date] *Sieber 57* (G00688993, K, M0241521); [without exact location] 1906, *L. Farmar 23* (BM); Dakar, 1948, *J.G. Adam s.n*. (K); Dakar, 1950–1951, *R.P. Berhaut 2094* (BR0000013833818); Cap Vert-Thiès Region, 8 Nov 1974, *D. Thoen 7322* (BR0000013833886);

**Somalia**: [Middle Shabelle Region] Mahaadei Uen [Mahaday Weyne], 1922, *R. Confalone 55* (FT); Jubba River, 1926, *P. Gorini 180* (FT); [Bakool Region] Xudur town, 17 Aug 1929, *L. Senni 658* (FT); Somaliland, Hargeisa, 4300 ft, 21 May 1932, *J.B. Gillett 3924* (FT, K); [Lower Shabelle Region] Merca town, May 1953, *T. Sacco & al. 56* (FT); [Jubaland State] Bardera, 30 Sep 1953, *P.R.O. Bally 9404* (K); Sagaleh Region, Migiurtinia, 23 Jan 1954, *G. Merla & al. s.n*. (FT); [Middle Shabelle Region] nr Mahaddei Uen [Mahaday Weyne] town, 13 Sep 1959, *G. Moggi & R. Ravazzano 2529* (FT); [Lower Shabelle Region] nr Danane, 21 Sep 1959, *G. Moggi & R. Bavazzano 2727* (FT, K); between Brava & Modun, 23 Sep 1959, *G. Moggi & R. Ravazzano 2783* (FT); [Lower Juba Region] Jubbada Hoose, Lake Salamo, 28 Oct 1971, *G. Moggi & R. Ravazzano s.n.* (FT); [Sahil Region] Sheikh town, 4600 ft, 6 Jun 1973, *J.R. Wood 137* (K); Mogadisho, 8 Sep 1975, *R. Ravazzano 1105* (FT); [Somaliland, Togdheer Region] Burao distr., 23 Dec 1977, *Kazmi & al. 126* (BR0000013834456, M); Central Rangelands, near sea coast, 5°57'N, 48°57'E, 28 May 1979, *J.B. Gillett & al. 22131* (K); [Sahil Region] Northern Rangelands, 9°59'N, 43°08'E, 1540 m a.s.l., 13 Jul 1981, *J.J. Beckett 1275* (K); [Lower Shabelle Region] Afgooye, 11 Aug 1981, *S. Pignatti s.n.* (FT); [Middle Shebelle Region] Mahaday Weyne vill., 28 Feb 1983, *R.J. Douthwaite 1* (K); [Lower Juba Region] nr Goobweyn vill., 15 Aug 1986, *C.F. Hemming & I. Deshmukh 187* (K); Middle Juba Region, 8 Sep 1986, *J. Madgwick 63* (K); [Lower Shabelle Region] Genale, Uebi Scebeli, 11 Nov 1986, *M. Tardelli 461* (FT); Shabeellaha Hoose Region, 47 km SW of Afgol [Afgooye], 60 m a.s.l., 11 Jun 1987, *V. Alstrup & A. Michelsen 146* (BR0000013837600, K);

**South Africa**: see type of *A.robusta*; [Mpumalanga Prov.] Lydenburg town, Dec 1894, *F. Wilms 1263* (AMD24173, BM, L1673919, M, WU); Johannesburg, Jan 1903, *Rand 1148* (BM); [Limpopo Prov.] nr Petersburg [Polokwane], 4000 ft, Feb 1904, *H. Bolus 11155* (BOL0291963); [North West Prov.] Rustenburg, 4500 ft, 13 Feb 1904, *O. Nation 168* (BOL0291962); [Mpumalanga Prov.] Nelspruit [Mbombela], Mar 1931, *Lieberberg 2363* (K); [Limpopo Prov.] Pietersburg, 28 Jun 1961, *Strey & Schlieben 8593* (M); Johannesburg, 14 Mar 1962, *M. Macnae 1471* (BOL0291956); KwaZulu-Natal Prov., Nsumu, 6 Apr 1966, *E.J. Moll 3149* (BR0000013835354); Transvaal Region, Johannesburg distr., 5300 ft, 29 Jul 1967, *J. Scheegers 1443* (M); Transvaal Prov., Silverton [nr Pretoria], 16 Feb 1979, A. *Balsinhas 3379* (BR0000013835293); Kruger NP, nr Crocodile Bridge, 24 Mar 2007, *O. Maurin 1393* (WAG1487727);

**South Sudan**: [Central Equatoria state] Bahl al-Jabal 22 Feb 1901, *anonymous 71* (K); Upper Nile State, Khor Daleib, 17 Jun 1929, *N.D. Simpson 7207* (BM, K); [Unity State] nr Bentiu, Nov 1936, *E.E. Evans-Pritchard 1* (K); [Eastern Equatoria State] nr Kapoeta, 30 Aug 1941, *Myers 13979* (K); [Jonglei State] Jongol’s Post, 30 Aug 1951, *M.I. Sherif 3993* (K); Ilemi Triangle, 1350 ft, 10 Aug 1969, *C.J. Carr 737* (BR0000013834258); 80 km N of Bor town, Djam-Djam, 440 m a.s.l., 19 Feb 1981, *J.M. Lock 41* (K);

**Sudan**: nr El Obeid town, Jul 1875, *J. Pfund 876* (K); Kordofan, Malbeis, 1879, *J. Pfund 286* (K); Khartoum, Dec 1902, *Broun 476* (K); El Gezira [Al Jzirah] State, Wad Medani town, 11 Nov 1965, *W.J.J.O de Wilde & al. 5731* (BR0000013834289, WAG1404410);

**Tanzania** (selected): [Kilimanjaro Region] Moshi distr., 9300 ft Oct 1927, *anonymous 788* (K); [Kagera Region] Bunazi vill., 1935, *H. Gillman 508* (K); [Katavi Region] Rukwa Lake, 3 May 1935, *Michelmore 1137* (K); [Shinyanga Region] Shinyanga town, Nov 1938, *anonymous 8155* (K); Rukwa valley, Kasanga Mbuga, Mar 1952, *W. Siame 132* (BM); [Mwanza Region] Mwanza, 22 Apr 1952, *R.E.S. Tanner 676* (K); [Tanga Region] Korogwe, 14 Jul 1952, *H.G. Faulkner 994* (BR0000013837709); nr Kwakuchinja, 1080 m a.s.l., 21 Jul 1956, *E. Milne-Redhead & P. Taylor 11192* (BR0000013835873, K); Tanga Region, Kigoma Region, Kibwesa town, Tanganyika Lake, 10 Jul 1958, *T.G. Jefford & al. 55* (BR0000013834999, K, WAG1404269); [Morogoro Region] nr Mgeta vill., 980 m a.s.l., 17 Jul 1958, *A. Gilli 155* (W0333233) together with *A.acuminata*; Lushoto distr., Mombo Forest Reserve, 1300 ft, 10 Sep 1960, *S.R. Semsei 3091* (K); [Tabora Region] Nzega distr., Webbere Plain, 1050 m a.s.l., 1961, *H.M. Richards 13472* (K); [Arusha Region] Olongogo, 14 Dec 1962, *J.B. Newbould 6384* (K); Tanga Region, Kideleko, 2000 ft, 29 Jun 1965, *M.E. Archbold 459* (K); Arusha Region, Arusha NP, 1371 m a.s.l., 17 Nov 1968, *H.M. Richards 23312* (K, M); [Iringa Region] Great Ruaha River, Msembe, 2700 ft, 20 Aug 1969, *P.J. Greenway & K. Kanuri 13781* (K); road from Longido to Engare Naibor, 1524 m a.s.l., 25 Mar 1970, *H.M. Richards 25680* (BR0000013837723, K); [Morogoro Region] Kilosa distr., 1700 ft, 25 Jun 1973, *P.J. Greenway & K. Kanuri 15235* (K); [Ngorongoro Conservation area] Ang’ata Kiti Plain, 30 Oct 1989, *S. Cuwa 3006* (K); Manyara Region, nr Babati vill., -4.053593, 35.775181, 29 Nov 2021, *A.P. Sukhorukov s.n.* (MW); Kilimanjaro Region, Moshi distr., way from Arusha town to Moshi vill., -3.335736, 37.284384, 15 Jun 2022, *A.P. Sukhorukov s.n*. (MW);

**Uganda** (selected): [Central Region] Kipayo vill., Aug 1914, *R. Dümmer 1006* (BM); 100 miles NW of Kampala, 4200 ft, Jun 1915, *E. Boernmueller 2681* (K); base of Debasien [Katam] Mount, Jan 1936, *W.J. Eggeling 2527* (K); Kasenyi, 27 May 1953, *D. van der Ben 472* (K); Tooro [sub-region], 25 Jan 1962, *J.P. Loveridge 459* (K); nr Kasanda, 10 Aug 1974, *A.B. Katende 2235* (K); Mt. Elgon, Sasa Trail, 3150 m a.s.l., 7 Jan 1997, *K. Wesche 739* (K); [Central Region] Mawokota County, Nkozi Hill, 20 Feb 2011, *S. Santini 393* (FT);

**Zambia**: [Western Prov.] Sesheke, 1910, *A.E. Gardner 486* (K); [Central Prov.] Mumbwa, 1911, *Macaulay 687* (K) together with *A.annua*; [Southern Prov.] Bambwe Forest, 1934, *J.D. Marin 643* (K); [Southern Prov.] Namwala distr., 1934–1935, *J.G. Read 36* (BM, K); [Western Prov.] Kalangola, Jul 1939, *H.J. Bredo 3168* (BR0000013708825); [Southern Prov.] Mapanza, 12 Mar 1953, 3500 ft, *E.A. Robinson 124* (BR0000013835149, K); Lusaka distr., 22 Jun 1956, A. *Angus 1348* (K); Tanganyika Lake, Casawa sand dunes, 1050 m a.s.l., 14 Apr 1957, *H.M. Richards 9219* (K); Northern Prov., Kampinda, 2950 ft, 25 Jul 1962, *P.J. Tyrer 113* (BM);

**Zimbabwe** (selected): Salisbury [Harare], 4800 ft, Mar 1906, *F. Eyles 299* (BOL0291961); Salisbury [Harare], 27 May 1914, *O. Craster 129* (K); Bulawayo distr., 1930, *E. Cheeseman 29* (BM); [Central Prov.] Concession town, Apr 1931, *R.W. Jack 3932* (BM); Salisbury [Harare] city, 27 Jul 1931, *H.B. Gilliland 85* (BM); Bulawayo town, Nov 1933, *A. Meebold 11801* (M); [Manicaland Prov.] Umtali [Mutare] city, 1956, *N.C. Chase 207* (BM); [Midlands Prov.] Gokwe [town], 23 Apr 1964, *M.G. Bingham 1292* (BR0000013835200, M); [Mashonaland East Prov.] Beatrice farm, 18 Apr 1972, *L.C. Leach 14885* (BR0000013835224); [Matabeleland North Prov.] Matetsi, 23 May 1975, *P. Gande 26* (K); [Mashonaland West Prov.] Great Dyke, 19 Feb 2000, *Th. Baudesson 154* (BR0000009048782, WAG1404330).

**Arabia**

**Oman**: nr Shurayjab, 6000 ft, 19 Mar 1972, *J.P. Mandaville 3619* (BM); nr Bani Habib vill., 5700 ft, 19 Mar 1972, *J.P. Mandaville 3640* (BM); 43 km North of Salalah, nr Aqabat al Hatab, 600 m a.s.l,, 21 Sep 1977, *A. Redcliffe-Smith 5126* (K); Dhofar, Jebel Qara, 180 m a.s.l., 23 Sep 1992, *S. Ghazanfar 2098* (BR0000023108004); Dhofar, Salalah to Thumrayt, 730 m a.s.l., 14 Sep 2002, *M. Raffaelli & al. 1196* (FT);

**Saudi Arabia**: nr Mekkah, 21 Nov 1857, *W. Schimper 944* (HBG503188, L1673920); 14 km E of Khamis Mushait town, 20 Oct 1969, *J.P. Mandaville 2522* (BM); Jeddah–Taif road, 3500 ft, 3 Feb 1980, *I.S. Collenette 1709* (K); Asir, 1984, *H.A. Fatih 35* (BM); Al-Bahah Region, 19 Feb 1987, *A.A. Fayed 1285* (M);

**Yemen**: Hadramaut, 15 May 1939, *H. Wissmann 3025* (BM); Kharaiba, Wadi Duan [Dawan], 26 Aug 1950, *K.M. Guichard 395* (BM); between Sana’a & Hodeidah, 13 Nov 1962, *G. Popov 20* (BM); T’aiz, Ibb road, 19 Nov 1971, *M. Brunt 2381* (BM); Jibla, 8 Jan 1974, *J.R.I. Wood 253* (BM); Sa’dah Prov., nr Furlanis camp, 22 May 1981, *P. Cuccuini 32-278* (FT); [Sanaa Governorate] 31 km ENE of Manacha [Manakhah], 5 Oct 1981, *anonymous 36453* (MSB122470); Ibb Governorate, nr Ibb, 2000 m a.s.l., 22 Jul 1983, *R. Spellenberg s.n*. (K); Dhala town, 1450 m a.s.l., 10 Jun 1987, *L. Boulos & al. 16717* (BM); Wadi Mursel, 2 km W of Hadaba Assufla vill., 14 Jan 1996, *M. Van Slageren & W. Saeed 189* (K); nr Sana’a, Wadi Dhar, 2500 m a.s.l., 9 Mar 1996, *P. Hein & E. von Raab-Straube 154 & 917* (W0333236, M); Al Mahrah Governorate, between Al Faydami & Hawf vill., 950–1150 m a.s.l., 23 Nov 1999, *P. Hein 6745* (E00540183, W0333225).

**Other Asian countries**

**India**: Madras [Chennai city] [without date, probably mid-19^th^ century, *anonymous s.n*.] (K); [Sate of Karnataka] Southern Maratha County & North Canara, 1879, *Schlagintweit s.n*. (BM); Kashmir Region (disputed area between India & Pakistan] Darar vill., 2 Sep 1887, *J.R. Drummond 26471* (K); [State of Karnataka] Hassan distr., 20 Sep 1971, *K.N. Gandhi 2058* (K); [State of Tamil Nadu] Dgarmapuri, 900 m a.s.l., 13 Nov 1979, *K.M. Matthew 24561* (K); [State of Karnataka] Mandya distr., 12 Feb 2014, *K.S. Rao & al. 139* (JSB-0278 – image!).

**Pakistan**: Punjab, [without date, mid-19^th^ century] *T. Thomson s.n*. (M); nr Karaha Lake [probably nr Sakesar], 3500 ft, 14 Sep 1902, *J.R. Drummond 14572* (K).

**Achyranthesseychellensis Sukhor**.

– see main text.

***Achyranthessicula* (L.) All.**

**Africa**

**Algeria** (selected): Algiers, Feb 1832, *W. Schimper s.n*. (L1673836); La Calle, 24 Jul 1843, *Durieu s.n.* (P04992333); Cherchell town, 1875, *E. Cosson s.n*. (K); El-Kettar, nr Dellys, 40 m a.s.l., 1877, *A. Meyer 1828* (FI, K); nr Algiers, Apr 1879, *C. Allard 2601* (K); Kouba, Jun 1879, *M. Gandoger s.n*. (BM); Bougie [Bejaia], May 1896, *E. Reverchon 84* (M, P0518080, WU); Guyotville [Ain Benian], 29 May 1919, *Ch. D’Alleizetti s.n*. (M, MA-01-00030124); [nr Algiers] Bouzaréah, 20 Apr 1953, *L. Delvosalle 3278* (BR0000023107991); Philippeville [Skikda town] 17 Aug 1954, *Doppelbaur 259* (M); between Algier & Bouira, 6 May 1971, *P.H. Davis 51963* (BM);

**Cape Verde**: Santo Antão Island, Agua das Caldeiras, 1430 m a.s.l., 23 Apr 2008, *J. Lambinon 77* (BR000000508604);

**Egypt**: Cairo, Nile valley, 1880, *G. Schweinfurth 229* (K); Cairo, 1886, *anonymous s.n*. (BR0000023108622);

**Morocco** (selected): Tanger, Apr 1871, *J. Ball s.n*. (K); Bou Regreg River, 1888, *anonymous s.n*. (K); Tanger, 25 Jun 1913, *C.-J. Pitard 1494* (K); Rabat, 21 Apr 1921, *E. Jahandiez s.n.* (RAB101204); Chaouia, 21 Apr 1924, *E. Jahandiez 167* (BM); Jebel Zem-Zem, 17 May 1930, *Pont Quer 174* (BM); Yassin, 25 Jun 1930, *Sennen & Mauricio 7700* (FI, RAB101207); Fedala [Mohammedia], Mar 1933, *anonymous 17* (K); Segangan, May 1934, *Sennen & Mauricio 9555* (BM); Douar Zyatene, 9 Jun 1956, *Bertault 157* (RAB101208); Oued Mellah [River], NE of Casablanca, 13 Apr 1972, *P.H. Davis 54423* (BM); Rabat, 19 May 1973, *J. Lewalle 7153* (BR0000023107984, M); Temara, 3 Mar 1976, *J. Lewalle 8195* (BR0000023108448, FI, RAB50567); Mehdia, 13 May 1978, *M. Naya s.n*. (BR0000024611862); Ain Harrouda, 5 Feb 1981, *D. Petit s.n*. (RAB64080); nr Rabat, 2 May 1987, *D. Podlech 43475* (M); Ladera NE of Jebel Imzi, 1150 m a.s.l., 16 Apr 2007, *T. Buira & J. Calvo 255* (MA-01-00758373, W0333274); Tiznit-Aït o Abderrahmane, 29.77347, -9.27243, 680 m a.s.l., 1 Feb 2021, *M. Chambouleyron s.n*. (CHAMB); Labaarir, 30.47088, -9.11964, 30 Dec 2021, *J.-F. Léger s.n.* (ECWP); Moullay Bousselham, Dlalha, 34.84363, -6.15838, 10 m a.s.l., 6 Oct 2023, *M. Chambouleyron s.n.* (ECWP);

**Spain** (Canary Islands; selected): Tenerife, Feb 1845, *E. Bourgeau 23* (BM, K005770144); La Palma, 400 m a.s.l., 13 Oct 1934, *M. Dinklage 3181* (BM, BR0000023108424, K005770160); Gran Canaria, 31 Jul 1940, *R.J. Andrews 29* (K005770154); Tenerife, Punte del Ancon, 26 Mar 1972, *J.L. de Sloover 72* (BR0000022706492); Hierro, 500 m a.s.l., 17 Mar 1973, *A.E. Aldridge 1317* (BM); Tenerife, Candelaria, 15 Mar 2004, *F. Verloove 5571* (BR0000012253821); Tenerife, 29 Nov 2008, *A. Seregin & I. Seregina s.n*. (MW10586318);

**Tunisia**: [Cape Bon] Sidi Rais, 24 Apr 1953, *Pottier-Alapetite s.n*. (MPU000626); [Nabeul Governorate] Hammamet, 9 Apr 1968, *H. Hertel 8221* (M); Ichkeul NP, 1978–1979, *J.M. Fay 932* (K).
